# Patterns of Oligonucleotide Sequences in Viral and Host Cell RNA Identify Mediators of the Host Innate Immune System

**DOI:** 10.1371/journal.pone.0005969

**Published:** 2009-06-18

**Authors:** Benjamin D. Greenbaum, Raul Rabadan, Arnold J. Levine

**Affiliations:** 1 Institute for Advanced Study, Princeton, New Jersey, United States of America; 2 Department of Biomedical Informatics, Center for Computational Biology and Bioinformatics, Columbia University College of Physicians and Surgeons, New York, New York, United States of America; National University of Ireland Galway, Ireland

## Abstract

The innate immune response provides a first line of defense against pathogens by targeting generic differential features that are present in foreign organisms but not in the host. These innate responses generate selection forces acting both in pathogens and hosts that further determine their co-evolution. Here we analyze the nucleic acid sequence fingerprints of these selection forces acting in parallel on both host innate immune genes and ssRNA viral genomes. We do this by identifying dinucleotide biases in the coding regions of innate immune response genes in plasmacytoid dendritic cells, and then use this signal to identify other significant host innate immune genes. The persistence of these biases in the orthologous groups of genes in humans and chickens is also examined. We then compare the significant motifs in highly expressed genes of the innate immune system to those in ssRNA viruses and study the evolution of these motifs in the H1N1 influenza genome. We argue that the significant under-represented motif pattern of CpG in an AU context - which is found in both the ssRNA viruses and innate genes, and has decreased throughout the history of H1N1 influenza replication in humans - is immunostimulatory and has been selected against during the co-evolution of viruses and host innate immune genes. This shows how differences in host immune biology can drive the evolution of viruses that jump into species with different immune priorities than the original host.

## Introduction

The innate immune system encompasses the non-specific response of an organism to broad classes of foreign pathogens [1]. At the cellular level, this process depends upon a cell's ability to recognize pathogenic material as “non-self” and react with an appropriate defense. In the past several years, a great deal of progress has been made in enumerating the different pattern recognition receptors (PRRs) that recognize sets of pathogen-associated molecular patterns (PAMPs) [2], [3]. Examples include the Toll-like receptors (TLRs), retinoic acid-inducible gene-I (RIG-I) like receptors, and Nod-like receptors (NLRs). The list may grow as experiments continue probing pattern receptor ligands both within and across species.

The ability of a pathogen to avoid or trigger recognition by the innate immune system will likely affect its survival. Therefore, advances in understanding immunostimulatory patterns cannot be separated from our understanding of pathogen evolution. In the evolution of RNA viruses, for instance, where the genetic mutation rate is orders of magnitude higher than in the host's genome, one would expect that a virus's evolutionary history strongly reflects its exposure to host recognition receptors. One should therefore be able to use an RNA virus replication time series to identify possible triggers of non-self material. If many different RNA viruses co-infecting the same host species have avoided certain sequence motifs for reasons that are not functional or structural, one may hypothesize that these patterns are PAMPs that the virus has evolved to avoid.

In parallel, the cells that are involved in the immune response might also avoid expressing genes that contain immunostimulatory motifs that can trigger cytokines toxic to the host as well as to the virus. The expression of immunostimulatory motifs in this situation could produce a confounding signal that can instigate an autoimmune reaction of potentially catastrophic consequences for the host, such as a cytokine storm. Hence, genes expressed in immune cells would avoid expressing these motifs and, consequently, one could potentially identify genes expressed by immune cells by analyzing the under-representation of these motifs. Therefore, the parallel search for similar under-represented motifs in viruses and their hosts can provide a method for identifying immunostimulatory motifs and the genes that encode them *in silico*. Finally, one can use the rapid time scale of viral evolution to search for empirical evidence of selection against these patterns as verification that they are in fact being avoided.

In this work we simultaneously analyze ssRNA virus evolution and the host innate immune response for evidence of biases that indicate parallel avoidance of nucleotide motifs that trigger an innate immune response. This study was inspired by our previous work, in which we noted that under-represented dinucleotide patterns in ssRNA viruses are often host specific and, moreover, have evolved in the genomes of many influenza virus strains, dating back to the pandemic 1918 H1N1 virus [4]. It is clear from that analysis that human influenza has been evolving in a manner that lowers CpG content in a way that lacks a straightforward explanation. In that paper we hypothesized that this change decreases recognition of the virus as non-self, even though there is no known innate immune receptor or other mechanism for recognizing CpG in ssRNA. There is known recognition of CpG in DNA (bound to by TLR9 [5]) and ssRNA (bound to by TLR 7 and 8 with possible context specificity [6]–[8]), along with experiments indicating that CpG in ssRNA may be immunostimulatory [9]. We believe this hypothesis, in part, due to observations in Ref. [4] comparing the dinucleotide distribution of coding sequence (CDS) regions in human and chicken mRNA. The human CDS regions have a lower CpG content than chickens, and we noted that some of the human genes with the lowest CpG content, such as type I interferons, are involved in innate immunity. We speculated in that work that the higher CpG signal in the 1918 virus could have contributed to its increased lethality via a high cytokine response and that a set of highly expressed innate immune genes may also avoid a CpG signal so as to avoid a deleterious positive feedback loop.

As expression is cell and tissue specific, the immune selection forces that act on RNAs in cells will be determined by the repertoire and quantity of innate immune recognition mechanisms that a cell type expresses. In this work we focus on RNA expression in plasmacytoid dendritic cells (pDCs). Discovered by O'Doherty, *et al.*, in 1994, pDCs are known to be the most efficient interferon producing cells [10], [11]. Despite comprising about 1% of peripheral blood mononuclear cells, pDCs are often the dominant producers of type I interferon in an antiviral response [12]–[14]. Therefore, if the innate immune response to viruses were to trigger harmful positive feedback via the production of cytokines, pDCs would be likely to highly express RNA involved in mediating this effect. These cells have already been implicated in autoimmune diseases such as systematic lupus erythematosus (SLE) and arthritis [15]–[17]. For instance, researchers have attempted to link SLE to sex-linked over-expression of TLR7, and, accordingly, to the over-sensitivity of the immune response to stimulatory ssRNA when pDCs engulf extra-cellular debris, such as may be generated following virus induced cell death [18]. The level of PRR expression could also change within immune cells during the anti-viral response, creating heightened sensitivity to an associated signal. While it is not clear that pDCs are the cell type involved in the recognition we are proposing, they are chosen here due to their favorable expression profile.

Clearly, there are many plausible scenarios where innate immune gene mRNA and ssRNA viruses may experience a common pressure to avoid triggering immunostimulatory signals as much as is possible throughout their evolutionary history. This pressure would become apparent at different time scales, given the comparatively rapid pace of viral evolution. For instance, if a DNA virus jumps from a species lacking TLR9 CpG sensitivity to a species expressing this receptor, then one would expect a change in CpG content as the virus evolves in its new host, possibly at a rapid rate if the initial virus had a very high CpG content. Likewise, innate immune genes, which are often expressed in large quantities only during an infection would avoid immunostimulatory signals so as to not create a positive feedback loop of cytokine autoimmune response when they are taken in by pDCs or other PRR expressing cells. Since this non-self signal is already low in the host and the rate of host genetic evolution is typically slower than in a virus, this will occur at a different rate.

Here we use public gene expression data generated by Iparraguirre, *et al.*, in mice [19] to identify significant nucleotide biases in highly expressed genes during an innate response. In these experiments, pDCs were stimulated with known influenza and CpG DNA agonists in order to trigger an innate immune response. Because this experiment examined pDC gene expression against the background of the whole mouse genome, one can measure how patterns in the expression data compare to those observed in the mouse genome as a whole, and better understand whether or not these patterns express a nucleic acid sequence signal that can be contrasted with the overall mouse CDS profile. Furthermore, we compare the under-represented motifs in innate immune expressed genes to those in primate ssRNA viruses.

In this work we examine the nucleotide signals of both innate immune genes CDS regions and ssRNA viruses to attempt to answer two questions. The first is whether innate immune genes contain a significant dinucleotide bias, such as that observed previously in ssRNA viruses. We test this by determining whether significant genes in mouse pDCs responding to influenza infection and CpG DNA have sequence biases. We then compare these biases across the genomes of humans and chickens since these species are more commonly associated with influenza. The second question, once this pattern is established, is whether or not the CDS regions of ssRNA viruses and the expressed innate immune genes share a similar pattern of over- and under-representation of nucleotide motifs, and thus, whether or not innate immune genes may have evolved to evade the same PRRs as ssRNA viruses so as to avoid a positive feedback loop. We verify this hypothesis over the 90 year time series of H1N1 influenza genome evolution in humans.

## Methods

The data used for this analysis was gathered from the following sources. The 47 primate viral genomes were taken from reference sequences in the NCBI (National Center for Biotechnology Information) Viral Genomes Resource (http://www.ncbi.nlm.nih.gov/genomes/VIRUSES/10239.html). These genomes were used for studies in which a single reference sequence represented each virus strain once, to allow for comparisons of classes of viruses. We use primate viruses, since we wish to filter out any species-specific effects that may result from the mouse model used for the gene expression data. For analysis of the evolution of influenza, genomes were taken from the Influenza Virus Resource at NCBI, (http://www.ncbi.nlm.nih.gov/genomes/FLU/FLU.html). This resource allowed us to examine a time series of multiple samples from several strains. We used 674 human H1N1, 24 human H2N2, 1499 human H3N2, 65 human H5N1, 170 influenza B, and 969 avian influenza nucleotide sequences from this resource in our previous work, but only the H1N1 data are directly employed here. The full lists of the ssRNA viral reference sequences and H1N1 viruses used in this analysis are available as Supplementary Tables S1 and S2.

For the expression profile of stimulated mouse (*Mus musculus*) pDC cells, the GCRMA normalized intensity data from Ref. [19] is used. However, the statistical analysis is performed separately in this work, since the questions asked are different than in the original experiment. The portion of that experiment used in this work consists of expression data for purified mouse pDC cells. These cells were stimulated with CpG DNA to trigger TLR9 and influenza RNA, presumably triggering TLR7. A control set was also prepared. The gene expression was studied by Iparraguirre, *et al.*, using mouse MOE430v2 Gene Chip Microarray (Affymatrix, Santa Clara, CA, USA). This data is publicly available from the NCBI Gene Expression Omnibus (http://www.ncbi.nlm.nih.gov/geo/) under the record number GSE7831. As in that work, we use the expression data generated 4 hours after stimulation to analyze the innate response. For subsequent analysis, the coding regions for all mouse genes were needed. All of these regions were taken from the NCBI Consensus CDS database (http://www.ncbi.nlm.nih.gov/projects/CCDS/CcdsBrowse.cgi). To correlate this information with homologous genes in humans (*Homo sapiens*) and chickens (*Gallus gallus*), the Human Genome Nomenclature Committee (HGNC) database is used to gather orthology predictions between mouse, human, and chicken [20]. We analyze the same coding information for the chicken and human genomes to see whether the patterns discovered persist in the orthologous genes in these species. As with the mouse CDS regions, the human CDS sequences are also taken from the NCBI Consensus CDS database [21], [22]. The chicken genome CDS regions are obtained from the University of California at Santa Cruz Genome Browser (http://genome.ucsc.edu/) [23]–[26].

We use several metrics to measure the significance of nucleic acid patterns in host and viral genomes. For the public expression data of pathogen stimulated mouse genes [19], we desire a single large expression value for each gene to avoid entangled data. For this value, we use the base 2 logarithm of the ratio of the mean expression level of the stimulated genes four hours after stimulation in each trial to the mean of the control set before stimulation. Thus the expression values listed later in this paper are base 2 logarithms of the average intensity in stimulated probes associated with a gene to control probes intensities associated with that same gene. As noted by Iparraguirre, *et al.*, four hours was the time at which most key antiviral cytokines were expressed. We then examine the mono- and dinucleotide frequency profile within the coding regions of most expressed genes compared to the CDS profile for the overall mouse genome. In the original experimental analysis of Ref. [19], 2-fold expression was used as the cutoff for designating a gene as significantly up-regulated. Therefore, we start with that level as a minimum cutoff for designating a gene as expressed.

Since we wish to examine whether the mono- or dinucleotide frequency is skewed in highly expressed genes, we vary the cutoff upwards by powers of two, and look at the frequency bias at each power of two until the number of genes is insignificantly small. To assess whether there is a greater abundance of expressed genes with low or high frequencies of mono- or dinucleotides compared to the overall genome, we divide the CDS frequency distribution of the overall genome for that mono- or dinucleotide into quartiles and tenths. We then use binomial statistics to evaluate, for each power of 2, the probability that the number of expressed genes that come from the highest and lowest tenths and quartiles of the overall genome frequency could have occurred randomly. In this way, we measure if the expressed genes have a significantly high or low abundance of a particular mono- or dinucleotide frequency when compared with all CDS regions of the genome. Moreover, for the case of dinucleotides, we also examine the significance of the ratio of the frequency of a dinucleotide over its expected value given nucleotide content, which we refer to as the *η* statistic, by the same procedure. This statistic, first used in this context by Karlin, *et al.*, [27], [28], was also used in our previous work [4]. It takes account of whether or not the observed dinucleotide frequency is merely due a particular nucleotide content, as could happen when a gene is located by chance in a region of the genome rich in a particular nucleotide, such as an isochore.

To look for over- or under-represented motifs across sets of genomes, such as all ssRNA viruses or all genes above a certain threshold expression level, we utilize the novel set of randomization algorithms for CDS regions employed in our previous work. This generates randomized data for each individual genome by creating a set of randomized genomes with the same amino acid composition, sequence, and codon distribution. However, for each amino acid, a codon is taken at random according to the true codon distribution of the real sequence. Nucleotide content will also be approximately preserved as a consequence. For a particular motif, one can count the number of times that motif appears in the real data and compare it to the average number of times it occurs in the randomized sequences to get a better sense of the bare pressures acting in excess of clear constraints. This average can be taken across an individual gene, across a whole genome, or across a set of genomes. For instance, if we wanted to find patterns that are over- or under-represented across all ssRNA viruses, we would concatenate the coding regions of each virus together and apply the randomization to each whole virus CDS region individually. However, we would take the averages across all of the viruses of interest and compare them to the total real count amongst all of the viruses. If we wanted to restrict ourselves to individual viruses, then we would take the average only for that virus. This method can also be applied to classes of host CDS regions, such as those that respond to an immune stimulus in a gene expression experiment. We employ this here to avoid concatenating small innate immune genes when exploring pressures beyond the dinucleotide level.

Once the real and expected value for a motif is obtained, we employ the following measure. If we let *N* denote the number of actual occurrences of a motif of a given length in a nucleotide sequence and *µ* denote the mean number of times that motif occurs in a randomized set of sequences, then the measure *ρ* is defined as 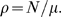 This measure is similar to the odds ratios used by Karlin, but it takes into account the protein sequence and codon bias, in addition to nucleotide content. In Ref. [4] we defined *ρ* only for dinucleotides that occur at the consecutive third and first positions across codons. The other positions of dinucleotides are generally accounted for entirely by the constraints of the algorithm: amino acid composition, nucleotide frequency, and codon bias. Hence, the above definition is a generalization of our previous one, applicable to longer oligomers. As the above is somewhat analogous to an odds ratio, one can derive confidence intervals by analyzing the motif distribution in the randomized sequences. Therefore, we can use this measure across classes of viral or host CDS regions to establish the significance of motifs. While we employ the *ρ* measure in this study, one can also use the comparison between real and expected values to construct exact p-values, as in [4], or other statistical measures that compare real and expected values, such as z-scores. All of these methods provide similar results.

## Results

**Figure 1:**
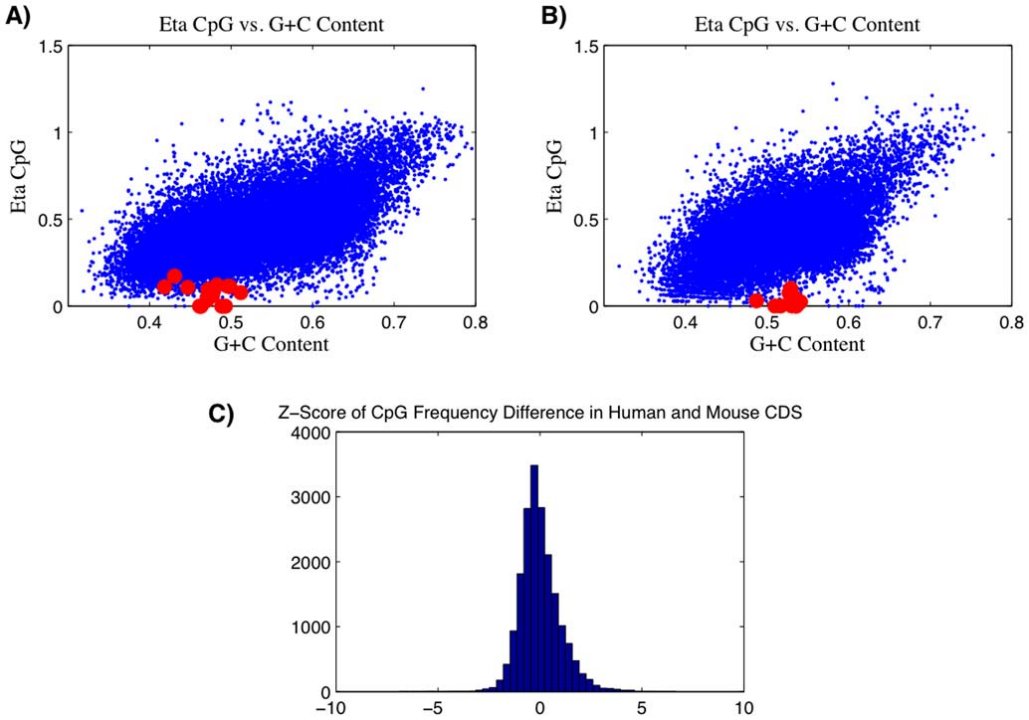
Comparison of CpG Content for human and mouse CDS regions. A) CpG odds ratio (*η*) versus C+G content for CDS regions of all human genes. The genes for Type I interferons are highlighted in red. B) CpG odds ratio (*η*) versus C+G content for CDS regions of all mouse genes. The genes for Type I interferons are highlighted in red. C) Standardized histogram of differences between CpG frequency for CDS regions in human versus mouse genes.

As a first step we compare the CpG profiles from mouse and human genes to determine if they are similar enough to warrant the use of mouse pDC expression data as the basis of this study. The CDS regions of the mouse genome follow a similar CpG dinucleotide pattern as the one reported in our previous work for human CDS regions. In Figure 1, we plot *η* as a function of GC content for both mouse and human CDS regions. Both overlap considerably and have almost identical median *η* values of approximately 0.42. The locations of the interferon alpha genes for both are highlighted in red. Like the human distribution observed in the previous paper, the mouse plot has many low CpG immune genes. Based on the similarity of these two graphs, as well as the extremely low CpG content of the interferon alpha genes, we expect that a mouse model will be comparable to a human model for determining if innate immune genes are avoiding CpG dinucleotides. Additionally, the correlation coefficient for CpG frequency between human and mouse CDS regions is 0.78. A histogram is shown of differences between CpG content in human and mouse genes, which are then standardized. The distribution is narrow and there is a small skewness (0.68) towards human genes. While most gene are not very different, some of the genes with the greatest differences are associated with HLA regions. The general trend suggests that a broad mammalian pattern may be operative here, and, in fact, we observe similar patterns to those seen in these graphs in all available genomes of the mammalian superorder Euarchontoglires, which split off from other mammals some 100 million years ago [unpublished observation by authors]. Therefore, the fact that we are using mouse expression data should not significantly constrain or bias this study.

**Figure 2:**
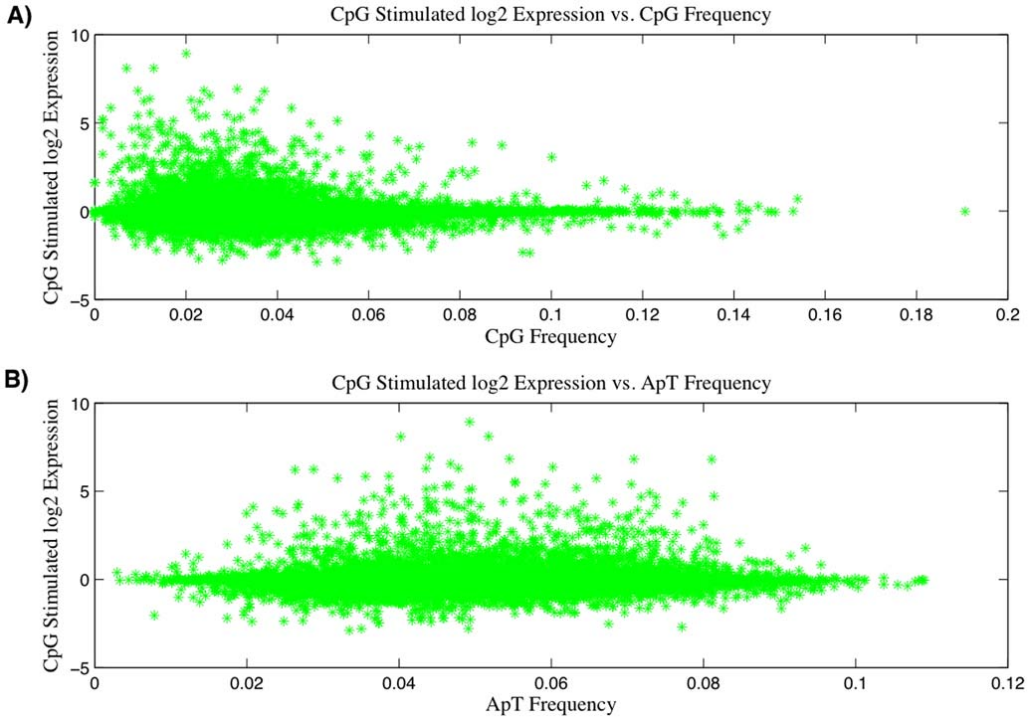
CpG bias in pDC expression vs CDS region CpG Content. A) log2 of the ratio of CpG stimulated pDC expression levels to control expression level vs. CpG frequency. B) log2 of the ratio of CpG stimulated pDC expression levels to control expression level vs. ApT frequency.

We next analyze the gene expression profile of stimulated mouse pDCs to look for dinucleotide biases in the most expressed genes. A visual examination of expression level versus CDS dinucleotide frequency gives a strong indication of low CpG bias in the most expressed genes. We show two plots in Figure 2. The first is the influenza stimulated expression level of mouse innate genes versus CpG frequency in CDS regions of those genes at the 4 hour mark. The most expressed genes are clearly shifted toward low frequency values. As a visual comparison, the second plot shows expression versus ApT dinucleotide frequency, which shows no apparant bias for influenza. Here it is visually clear that the genes come randomly from the distribution. Analogous plots of expression level versus *η* look simlar to those shown here.

These observations are statistically verified to insure that observed low CpG bias is not a result of the low CpG frequency of the genome in general. This step is performed for all dinucleotides, so that any biases are noted. We start by examining genes at the 2-fold expression level, following the original reference, as our initial cutoff for increased levels of regulation after exposure to influenza or CpG DNA [19]. For each dinucleotide, we calculate the probability that the amount of expressed genes whose dinucleotide frequencies are from the highest and lowest tenths and quartiles of all genes could have arisen randomly from the overall genome distribution for that dinucleotide frequency. We check this further at each additional two-fold expression level, as described in the methods. For clarity of presentation, Table 1 and 2 only show dinucleotide bias in the intersecting set of both CpG and influenza stimulated genes at the 2- and 4-fold levels. There is a clear pattern. At the 2-fold level, there are several genes whose frequency distribution appears skewed at the quartile level when compared to the whole genome distribution. For instance, CpG, GpC, and GG have far more expressed genes in the lowest quartile, while GA has more in the highest quartile. However, when one looks at the lowest and highest tenths, only CpG is highly significant, with a p-value of less than 10^−5^, indicating a strong pressure for the expressed genes to have a low CpG frequency, even compared to the already low CpG content of the entire genome.

**Table 1:** The probability that the amount of 2-fold expressed genes in the lowest and highest 10% and 25% could have occurred randomly given the whole genome distribution, calculated for both the frequency and *η* distributions of a dinucleotide (probabilities less than 0.00001 are listed as 0).

	Dinucleotide Frequency				*η*			
	Low 25%	High 25%	Low 10%	High 10%	Low 25%	High 25%	Low 10%	High 10%
CG	0.00014	-	0	-	0.00080	-	0	-
CT	-	-	-	-	-	0.00367	-	-
GA	-	0.00080	-	-	-	0.00585	-	0.00078
GC	0.00367	-	-	-	-	-	-	-
GG	0.00584	-	-	-	-	-	-	-
TC	-	-	-	-	-	0.00367	-	-

**Table 2:** Biases in expression data that persist at the 4-fold expression level.

	Dinucleotide Frequency				*η*			
	Low 25%	High 25%	Low 10%	High 10%	Low 25%	High 25%	Low 10%	High 10%
AA	-	0.00005	-	0.00033	-	-	-	-
AT	-	0.00693	-	-	-	-	-	-
CC	0.00477	-	-	-	-	-	-	-
CG	0.00059	-	0	-	0.00477	-	0.00002	-
GA	-	0.00693	-	-	-	0.00692	-	0.00338
GC	0.00001	-	0.00568	-	-	-	-	-
GG	0.00324	-	-	-	-	-	-	-
TC	-	-	-	-	-	0.00014	-	-

The second set of columns in these tables show dinucleotiode biases for the *η* statistic, rather than the CpG frequency, at each level of expression. As a reminder, this statistic takes nucleotide bias into account when determining dinucleotide representation. Here a more refined story emerges. Only CpG is skewed lower than in the general distribution. GA is skewed higher, though the p-value is 1,000 times larger than that of CpG. The message is clear: the most expressed set of genes has a much stronger pressure to have a low CpG content than do mouse genes in general, and this pressure cannot be explained by nucleotide frequency alone. That is, these genes are not low in CpG simply because they are located in a low GC-content region. This CpG pressure is clearly the most significant dinucleotide characteristic of the expressed genes.

This conclusion is further reinforced by the data in Table 3, where we look at the tenths and quartiles for single nucleotide content and AT and GC content at the 4-fold levels, as there is no significance at the 2-fold level. This analysis also further clarifies whether or not the dominant statistical pressure is GC bias or CpG pressure, indicating experimental error that should be accounted for in the original GCRMA normalization procedure. At the single nucleotide level, shown in Tables 4 and 5, A content is persistently abundant in the highest 10% level and G and C content are persistently scarce at the quartile level. There is also a lower amount of GC content and a higher amount of AT content, though the p-values associated with these effects are lower than the p-values of those for CpG dinucleotides, indicating that CpG is the dominant pressure. Combining these facts with the highly significant *η* values for low CpG implies that CpG pressure is the driving force behind the relatively low GC content and high A content in this location compared to the rest of the genome. In our previous work we noticed that the lowering of CpG in the H1N1 genome over 90 years of infection in human hosts appeared to be compensated for by an increase in TpA. In this case, there is a relative abundance of GA. Both cases favor an increase in A content, presumably from G to A mutations, as a byproduct of CpG avoidance. This is also reinforced by the significant A abundance without a compensating T abundance. Thus we conclude that genes encoding functions of the innate immune response avoid CpG in coding regions – as we hypothesized they should – to avoid a positive feedback loop of innate immune response.

**Table 3:** GC and AT content bias in 4-fold expressed genes, which are generally weaker and can be accounted for by the dinucleotide bias.

4-fold	Low 25%	High 25%	Low 10 %	High 10 %
AT%	-	0.00023	-	0.00061
GC%	0.00023	-	0.00061	-

**Table 4:** Nucleotide content bias in 2-fold expressed genes, which are again weaker and can be accounted for by dinucleotide bias.

2-fold	Low 25%	High 25%	Low 10%	Hi 10%
A%	-	-	-	-
C%	-	-	-	-
G%	0.00911	-	-	-
T%	-	-	-	-

**Table 5:** Nucleotide content bias in 4-fold expressed genes.

4-fold	Low 25%	High 25%	Low 10%	High 10%
A%	-	0.00059	-	0.00568
C%	0.00991	-	-	-
G%	0.00009	-	-	-
T%	-	-	-	-

We further use these data to classify important innate immune genes by combining high expression with significant CpG avoidance. In Table 6, we look at the median frequency of the 4-fold expressed genes in pDCs that are both exposed to influenza and are in the lowest tenth of CpG frequency. Next, we compare the CpG content of this group of genes to their homologs in human and avian mRNA derived from the HGNC database. The median CpG frequency of homologs of this gene group is even lower in humans than in mice. However, in chickens, the CpG frequency is much higher, suggesting that this CpG pressure is less significant in an avian influenza host. The genes in this group are listed in Table 7. The genes with the largest difference in CpG content between human and chicken orthologs from within this group are predominantly cytokines. The largest difference is found in the cytokine IL6. Its fraction of CpG as a total percentage of dinucleotides is 0.0094 in mouse and 0.016 in human, while it is 0.10 in chicken. In fact, while the *η* value for this gene is 0.22 in mouse and 0.25 in human, in chicken it is a close to the expected value of 1.03. Following closely behind IL6 are the Type I interferon genes and IL1-type cytokines. Thus a comparison of many innate immune genes expressed in mouse pDCs infected with influenza shows that these genes have significantly low CpG dinucleotide levels in mouse and human, but not in chickens. This mirrors the CpG levels in human versus chicken influenza from [4].

**Table 6:** Median values of the CpG frequency influenza induced expressed mouse genes in the lowest 10% of overall mouse genome CpG frequency, along with the median CpG frequency in orthologous avian and human genes.

	Mouse	Avian	Human
Flu	0.0112	0.0311	0.0083

**Table 7:** List of the expressed mouse genes in the lowest 10% of mouse genome CpG frequency.

Serpinb2	0.0104
Rgs1	0.0119
Ptgs2	0.0143
Ifrg15	0.0051
Xcl1	0.0116
Fcgr4	0.0134
Mnda	0.0094
Ifi205	0.0074
Il1rn	0.0146
Prg3	0.009
Il1b	0.0136
Rnf24	0.0112
Tnfsf10	0.0149
Serpini1	0.0122
Fnbp1l	0.0135
Gbp2	0.0141
Ifi44	0.0103
Cpne3	0.0125
Mup2	0.0129
Mup1	0.0129
Ifna2	0.007
Ifna5	0.0035
Ifna4	0.0018
Ifna1	0.0035
Il6	0.0094
Tlr1	0.0113
Mpa2l	0.0136
Cd33	0.0139
Phca	0.0125
Gvin1	0.0059
Casp4	0.0107
Bcl2a1a	0.0116
2700019D07Rik	0.0106
Aftph	0.0118
Rel	0.0147
Tgtp	0.012
Fbxo39	0.0145
Gpr33	0.0108
Serpinb1b	0.007
D14Ertd668e	0.0094
Bex6	0.0058
EG240327	0.0148
Iigp1	0.0097
Ms4a4c	0.0088
Ms4a4b	0.0088
Ms4a6b	0.0082
Ms4a6d	0.004
Ifit2	0.012
Ifit1	0.0129
Cenpi	0.0138
Armcx6	0.0122
Cflar	0.0124
Pyhin1	0.0111
Ifi204	0.0075
Ifi203	0.0049
Ccl4	0.0108
1100001G20Rik	0.0052
Setdb2	0.014
Kpna3	0.0147
Apol7a	0.0103
Apol7c	0.0081
BC031441	0.0025
Il1rn	0.0131
Ifna6	0.0018
Zfp800	0.0146
Klrk1	0.0129
Klrk1	0.0106
AI451617	0.006
Il15	0.0082
BC050092	0.0146

Now that a dinucleotide pattern has been established, we can focus our attention on longer over- and under-represented motifs in viral genomes and the CDS regions of innate immune expressed genes. We focus on motifs of length four, rather than six or eight, since shorter motifs afford a greater statistical accuracy and we desire sequences greater than codon length. We cannot do this with individual genes, due to the short length of several innate immune genes, so we randomize within individual genes, but count the occurrence of motifs across a whole class of highly expressed genes. To qualify a motif as over- or under-represented, we use the criteria given by Karlin in Ref. [28]. In that work, an odds ratio of greater than 1.23 is deemed over-represented and less than 0.78 is deemed under-represented. The justification given in that work is that the distribution of the log-odds ratio approaches a normal distribution. This threshold corresponds to a probability of less than 0.001 that an odds ratio value for a dinucleotide would occur randomly in a sequence greater than 5 kb.

In the innate genes, the approach of Ref. [28] yields many significant CDS motifs at the 2-, 4-, and 8-fold mRNA expression levels in the influenza stimulated genes, the CpG stimulated genes, and the intersection of these groups. There is a strong overlap between the under-represented motifs discovered. There are 64–70 motifs in each group with the difference coming from marginal cases close to the 0.78 cutoff value. Table 8 lists the ten most under-represented motifs from the intersection of all pathogen-stimulated groups. Next to them we list the ten most under-represented motifs from the ssRNA viruses, for which we performed the same search procedure. The most significant motifs in the innate immune genes and the viruses that are the targets of the innate immune response all contain CpG and have a strong overlap: six of the ten most under-represented motifs are identical. This clearly implies a specific sequence pressure common to both groups. Table 9 shows the same results for over-represented motifs. In Supplementary Tables S3, S4, S5, S6, S7, S8, we list all of the motifs that qualify as over- or under-represented from each group, as well as a table of the under- and over- represented motifs from both of the viral and expressed gene groups. The genes, while having many common motifs with the viruses, tend to have stronger pressures and more significant motifs as a consequence. They also have some non-CpG related motifs, unlike the viruses.

**Table 8:** The 10 most under-represented quadrameric motifs in stimulated pDCs and ssRNA viruses, listed in order of ascending *ρ* values.

Stimulated Genes		ssRNA Viruses	
CGAA	0.431	CGAT	0.540
TCGA	0.450	CGAA	0.542
CGAT	0.467	TTCG	0.548
GTCG	0.470	ACGA	0.548
CGTA	0.470	TCGA	0.549
GCGA	0.481	GACG	0.564
TACG	0.511	TACG	0.583
CGAC	0.515	TCGT	0.596
ACGA	0.524	GTCG	0.600
CCGA	0.544	ATCG	0.607

**Table 9:** The 10 most over-represented quadrameric motifs in stimulated pDCs and ssRNA viruses, listed in order of descending *ρ* values.

Stimulated Genes		ssRNA Viruses	
TTTG	1.35	ACCA	1.29
ATGA	1.35	CCAC	1.27
TGTG	1.34	ACAC	1.26
GATG	1.32	CACA	1.25
CCAT	1.31	TGTG	1.24
GTGG	1.29	CACC	1.24
ACCA	1.27		
CTGG	1.27		
CATC	1.27		
GCTG	1.26		

**Figure 3:**
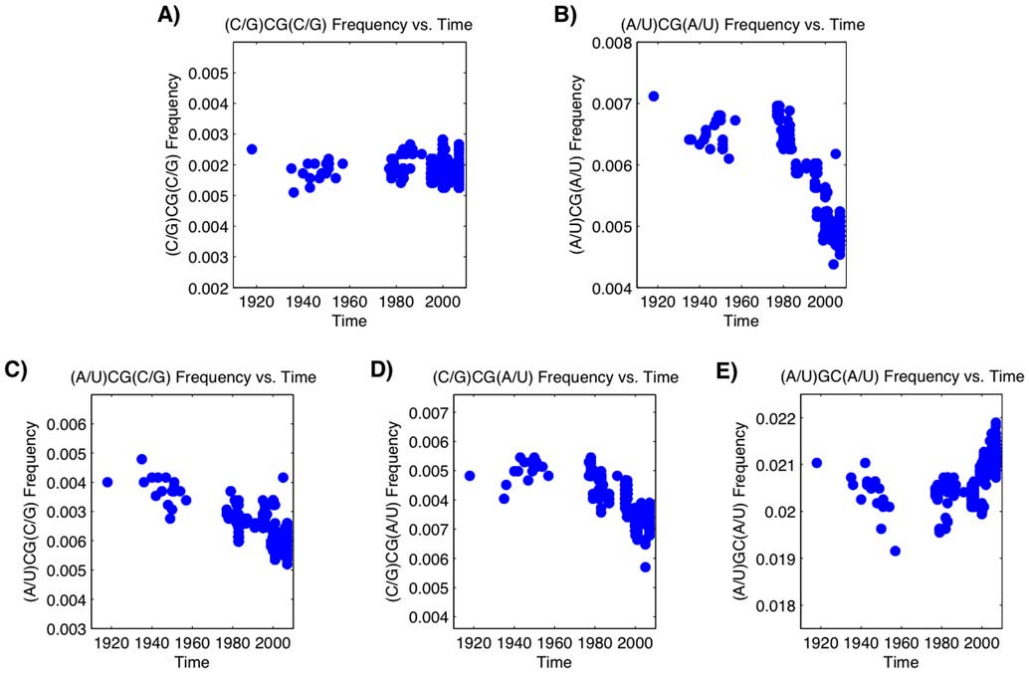
Evolution of the CpG frequency of H1N1 influenza A virus in time, binned by CpG context for the following four sets of bins: A) CpGs “protected” by Cs and Gs. B) CpGs surrounded by As and Us. C) CpGs with a C or G on the right and an A or U on the left. D) CpGs with a C or G on the left and an A or U on the right. E) In contrast, this figure shows GpCs surrounded by As and Us. There is no decline as observed in (B).

Finally, we examine the 90 year evolution of H1N1 influenza in humans by looking at the context in which CpG dinucleotides occur. The virus disappeared from the population in 1957 and reemerged in 1977. The 1977 virus was anitgenically similar to many 1950s H1N1 strains and, as a result, is thought to be the result of a frozen sample that may have escaped from a lab [29]. It is clear from Table 8 that the most under-represented motifs have CpG in a context of As and Ts. Figure 3 of Ref. [4] shows a clear decline in the CpG content of human influenza genomes. We now separate the CpGs in the time series of H1N1 genomes into four groups. The first is 4-mers of the form (C/G)CG(C/G), that is, a CpG with either a C or a G on the left and a C or a G on the right. The second is the group (A/U)CG(A/U), the third is (A/U)CG(C/G), and the fourth is (C/G)CG(A/U). Figure 3 shows clearly that, as the CpG frequency changes over time, the majority of the change comes from eliminating the CpGs surrounded by A and U nucleotides. In fact, the CpG dinucleotides surrounded by C and G nucleotides are *unselected*; there is no discernable change in their content. On the other hand, CpGs with a C or a G on one side and an A or a U on the other, show a decline, but at approximately half the rate as if there were an A or a U flanking both sides of the CpG. Hence, it is not just CpGs that are being targeted, but CpGs in a specific context. For contrast, it is also shown that (A/U)GC(A/U) does not show a similar decline as (A/U)CG(A/U), further establishing that GC content is not responsible for this effect.

The fraction of CpGs flanked by As and Us starts at 0.0071 in the 1918 H1N1 and ends with a median value of 0.0048 in the 2007 H1N1 samples. This initial value is higher than the median value of 0.0064 from the avain viruses in the database and is slightly higher than the median value of 0.0069 found in the lethal H5N1 virus, though some of those samples were slightly higher than in the 1918 flu. Comparatively, Influenza B has a value of 0.0042 and does not decline, suggesting that the recent H1N1 is now closer to equilibrium, like Influenza B, than to the more lethal variants. The expected pattern exists for the other groups, with the (C/G)CG(C/G) bin having similar values amongst all samples and the mixed motifs behaving like the (A/U)CG(A/U) bin, but more weakly. The (A/U)CG(A/U) group, however, seems to be the clearest target of possible selective pressure, as one would predict from the most under-represented motifs in Table 8.

## Discussion

From the above analysis, it is clear that there are shared di-, tetra-, and hexanucleotide sequence biases between ssRNA viruses and the mRNAs of host innate immune genes. These common evolutionary pressures could be due to common mutational processes or common selection forces. Hosts and ssRNA viruses do not share common polymerases or replication machinery suggesting that the most likely explanation is that common factors act in a parallel way in host and pathogen. Hence we conclude that the genomes of both influenza viruses and their hosts share a common evolutionary selection pressure at the nucleotide sequence level to eliminate CpGs in an AU context. By contrast, some hosts, like avian species, have a less robust innate immune response to these pressures and, therefore, tolerate influenza viruses with a greater diversity of CpG frequencies. When a virus with a very high CpG frequency jumps into the human population from the avian reservoir, the human hosts may suffer from an acute innate immune response. If this virus can still replicate under these conditions, the CpG frequency will become attenuated over time. In parallel, innate immune genes experience the same pressures to avoid confounding autoimmune signals that could trigger harmful positive feedback loops of cytokine expression. If host mRNA has evolved to avoid a set of immunostimulatory receptors, then this RNA must have a heightened level of exposure to PRRs at some points during the innate response that would trigger receptors were there PAMPs present. Hence, if an individual developed an abnormal sensitivity via an allele to stimulating motifs, this could clearly trigger a detrimental feedback loop and an associated autoimmune disease. This logic, therefore, predicts which genes to examine in some autoimmune diseases.

The evidence for the above comes from comparing the low CpG selection pressure in the innate immune genes observed here to the viral CpG selection pressure observed here and in Ref. [4]. In both cases, avoidance of CpG is the strongest signal present. While that is suggestive, most convincing is the striking similarity of underrepresented motifs of longer oligonucleotides. The nature of the common pattern is clearly CpG in an AU context. It is clear from Figure 3 that this is the CpG motif that the H1N1 virus has been most strongly selecting against. Moreover, of the shared motifs between the viruses and host genes, the 12 with lowest *ρ* values all involve CpG surrounded by As and Us, and with similar ranking. Certainly, if a known TLR that recognized CpG in RNA existed, one would conclude that it is the cause of the observed pattern. However, here we use the commonality of host and pathogen motifs to predict the existence of a PRR for CpG in RNA. The existence of such a receptor has some support, as CpG rich ssRNA has been observed to stimulate an immune response [9]. Moreover, these motifs are very similar to those for CpG DNA that stimulates TLR9, as TLR9 binds to CpG in an AT rich DNA context preferentially [30]. Thus, the notion of using overlapping host and pathogen signals for PRR prediction seems plausible as this work clearly predicts a (U/A)CG(U/A) RNA receptor or, at the very least, some other equivalent mechanism driven by a nucleic acid signal.

Viral databases of nucleotide sequences offer an additional opportunity because RNA viruses evolve rapidly on short time scales. H1N1 influenza, which is known from our previous work to have been lowering its CpG content over the past 90 years in human hosts, clearly has been doing so in a heavily biased fashion. The 1918 virus began its life at a level of CpG in an AU context most associated with avian viruses and only seen comparably in human strains in the highly pathogenic H5N1 virus. It has been evolving to lower this signal ever since, and is currently at a level closer to Influenza B, a virus in equilibrium with the human population over a comparatively long time frame. Likewise, when looking across species, the innate immune genes that most avoid theses motifs do so in human and mouse, but not in chicken. This empirically supports the speculation of the previous work that the virulence of the 1918 strain, and the cytokine storm implicated as a mechanism of high pathogenesis in this strain [31], may have been due in part to an abundance of non-self avian CpG signal that overstimulated the human innate immune system. Moreover, recent experiments suggesting that the 1918 virus had a more efficient polymerase than current circulating H1N1 strains [32] would further support the results here, since this would have caused the 1918 virus to present even more immunostimulating motifs to PRRs.

The degree to which the biases in genes identified by this study are representative of trends in the innate immune response as a whole is not clear. The public mouse expression data used in this study uncovered a set of highly expressed pDC genes for CpG in DNA and a different, but strongly overlapping, set from influenza. This raises a general question of what genes are broadly expressed when a pathogen stimulates a cell and what genes are only expressed for a specific set of pathogens and cell types. In humans, myeloid dendritic cells express TLRs 7 and 8, whereas the pDCs studied here express TLR 7 and TLR 9 [13]. Certainly, a one would like to repeat the experiments of Ref. [19] in human pDCs, as well as in other relevant human and non-human cell types. Studies of the innate immune response between cell types can differentiate the specificity of these receptors and further characterize the important non-self RNA signals. Our approach could be generalized beyond RNA to other non-self patterns in proteins or other materials.

Since different pathogens cause different gene expression patterns of innate immunity, it would be naïve to assume that we have fully probed the extent to which gene expression is pattern specific. While it is clear from this study that expressed innate genes are often drastically low in CpG, it is not clear to what extent this drives overall CDS CpG pressure. CpG is certainly low for many reasons, and in DNA, methylation and deamination are considered the primary causes [33]–[35]. However, we would hypothesize that this or some other process led to CpG in RNA, as well as the CpG in DNA detected by TLR 9, becoming a non-self signal in certain mammals. This further drove a subset of genes in these species to have an *extremely* low CpG content, even compared to the genome as a whole. The group of genes with a CpG frequency amongst the lowest 10% of CDS regions contains many other genes that were not expressed by either of the two stimulants here. A complete list of these genes is provided in Supplementary Tables S9 and S10. It will be interesting to learn what fraction of these genes have a role in innate immunity, and to what extent extremely low CpG content is driven by innate immune pressure. There are many suggestive genes with low CpG frequency, such as C-type lectin doman family and zinc finger genes, along with other cytokines, that likely have innate immune functions. However, genes such as olfactory receptors and keratin-associated proteins also have low CpG frequency and their connection to innate immunity is not clear. This paper points out a new set of criteria to classify the innate immune response. As such, new genes are identified that we have presently no reason to classify as part of the innate immune response, but nevertheless may be. Only by continuing to probe a diversity of host cell types with a diversity of pathogens, and by quantifying the unusual patterns common to both, will we discover the extent to which the self/non-self distinction has simultaneously driven both host and pathogen evolution.

## Supporting Information

Table S1 A list of all the H1N1 viruses whose CDS regions were used in this analysis.
(0.14 MB DOC)

Table S2 The ssRNA viruses whose CDS regions were used in this analysis.
(0.05 MB DOC)

Table S3 The 56 under-represented motifs of length four, listed in order of ascending p-values, that are significant in all groups of expressed genes at the 2-, 4- and 8-fold level and are taken from the CDS regions of these genes.
(0.08 MB DOC)

Table S4 The 13 over-represented motifs in expressed genes by the same criteria as Table S3, ranked in descending order.
(0.04 MB DOC)

Table S5 The genes whose CpG frequency is in the lowest 10% of the mouse genome, with the gene name and Entrez ID gene numbers both listed. B) The lowest genes bythe same criterion in the human genome.
(0.06 MB DOC)

Table S6 The lowest genes by the same criterion as Table S5, but in the human genome.
(0.03 MB DOC)

Table S7 The 33 shared under-represented motifs for the genes and viruses, ranked by the viral p-value in ascending order.
(0.05 MB DOC)

Table S8 The two shared over-represented motifs for genes and viruses, in descending order.
(0.03 MB DOC)

Table S9 The genes whose CpG frequency is in the lowest 10% of the mouse genome, with the gene name and Entrez ID gene numbers both listed.
(1.22 MB DOC)

Table S10 The lowest genes in the human genome, by the same criteria as in Table S9.
(1.05 MB DOC)
