# Supplementary material for: Patterns of Oligonucleotide Sequences in Viral and Host Cell RNA Identify Mediators of the Host Innate Immune System
Source: PLoS One. 2009 Jun 18;4(6):e5969. doi: 10.1371/journal.pone.0005969 (PMC2694999; doi:10.1371/journal.pone.0005969)
Supplement: Table S1 — A list of all the H1N1 viruses whose CDS regions were used in this analysis. (0.14 MB DOC) [file pone.0005969.s001.doc]

gb|AF333238| Influenza A virus (A/Brevig_Mission/1/18(H1N1)), complete sequence

gb|CY020449| Influenza A virus (A/Henry/1936(H1N1)), complete sequence

gb|CY016647| Influenza A virus (A/New South Wales/18/1999(H1N1)), complete sequence

gb|CY017127| Influenza A virus (A/New South Wales/24/1999(H1N1)), complete sequence

gb|CY016264| Influenza A virus (A/New South Wales/26/2000(H1N1)), complete sequence

gb|CY016567| Influenza A virus (A/New South Wales/30/2000(H1N1)), complete sequence

gb|CY016671| Influenza A virus (A/South Australia/45/2000(H1N1)), complete sequence

gb|CY016727| Influenza A virus (A/South Australia/63/2000(H1N1)), complete sequence

gb|CY016735| Influenza A virus (A/South Australia/64/2000(H1N1)), complete sequence

gb|CY017023| Influenza A virus (A/South Australia/29/2000(H1N1)), complete sequence

gb|CY017143| Influenza A virus (A/South Australia/25/2000(H1N1)), complete sequence

gb|CY017151| Influenza A virus (A/South Australia/36/2000(H1N1)), complete sequence

gb|CY017159| Influenza A virus (A/South Australia/46/2000(H1N1)), complete sequence

gb|CY017383| Influenza A virus (A/South Australia/26/2000(H1N1)), complete sequence

gb|CY017817| Influenza A virus (A/South Australia/30/2000(H1N1)), complete sequence

gb|CY017833| Influenza A virus (A/South Australia/39/2000(H1N1)), complete sequence

gb|CY018937| Influenza A virus (A/South Australia/40/2000(H1N1)), complete sequence

gb|CY021753| Influenza A virus (A/South Australia/44/2000(H1N1)), complete sequence

gb|CY019927| Influenza A virus (A/Western Australia/20/2001(H1N1)), complete sequence

gb|CY020273| Influenza A virus (A/Western Australia/18/2001(H1N1)), complete sequence

gb|CY020281| Influenza A virus (A/Western Australia/19/2001(H1N1)), complete sequence

gb|CY020433| Influenza A virus (A/Western Australia/22/2001(H1N1)), complete sequence

gb|CY016679| Influenza A virus (A/South Australia/55/2005(H1N1)), complete sequence

gb|CY016687| Influenza A virus (A/South Australia/56/2005(H1N1)), complete sequence

gb|CY016695| Influenza A virus (A/South Australia/57/2005(H1N1)), complete sequence

gb|CY016703| Influenza A virus (A/South Australia/58/2005(H1N1)), complete sequence

gb|CY020161| Influenza A virus (A/Western Australia/77/2005(H1N1)), complete sequence

gb|CY021761| Influenza A virus (A/South Australia/51/2005(H1N1)), complete sequence

gb|CY020297| Influenza A virus (A/Brazil/11/1978(H1N1)), complete sequence

gb|CY020441| Influenza A virus (A/Chile/1/1983(H1N1)), complete sequence

gb|CY013817| Influenza A virus (A/Nanchang/13/1996(H1N1)), complete sequence

gb|CY013825| Influenza A virus (A/Nanchang/15/1996(H1N1)), complete sequence

gb|CY013833| Influenza A virus (A/Nanchang/16/1996(H1N1)), complete sequence

gb|CY013841| Influenza A virus (A/Nanchang/17/1996(H1N1)), complete sequence

gb|CY013849| Influenza A virus (A/Nanchang/19/1996(H1N1)), complete sequence

gb|CY013857| Influenza A virus (A/Nanchang/26/1996(H1N1)), complete sequence

gb|CY016056| Influenza A virus (A/Nanchang/21/1996(H1N1)), complete sequence

gb|CY016232| Influenza A virus (A/Nanchang/8/1996(H1N1)), complete sequence

gb|CY016240| Influenza A virus (A/Nanchang/11/1996(H1N1)), complete sequence

gb|CY016248| Influenza A virus (A/Nanchang/23/1996(H1N1)), complete sequence

gb|CY016256| Influenza A virus (A/Nanchang/25/1996(H1N1)), complete sequence

gb|CY017007| Influenza A virus (A/Nanchang/9/1996(H1N1)), complete sequence

gb|CY017015| Influenza A virus (A/Nanchang/14/1996(H1N1)), complete sequence

gb|CY009296| Influenza A virus (A/Hong Kong/117/77(H1N1)), complete sequence

gb|CY020457| Influenza A virus (A/India/6263/1980(H1N1)), complete sequence

gb|CY019975| Influenza A virus (A/Roma/1949(H1N1)), complete sequence

gb|CY028095| Influenza A virus (A/Tennessee/UR06-0523/2007(H1N1)), complete sequence

gb|CY009344| Influenza A virus (A/Malaysia/54(H1N1)), complete sequence

gb|CY021057| Influenza A virus (A/Malaya/302/1954(H1N1)), complete sequence

gb|DQ508861| Influenza A virus (A/New Caledonia/20/1999(H1N1)), complete sequence

gb|CY020193| Influenza A virus (A/New Zealand/7/1983(H1N1)), complete sequence

gb|CY025030| Influenza A virus (A/Auckland/584/2000(H1N1)), complete sequence

gb|CY026159| Influenza A virus (A/Auckland/585/2000(H1N1)), complete sequence

gb|CY025038| Influenza A virus (A/Auckland/597/2000(H1N1)), complete sequence

gb|CY009176| Influenza A virus (A/South Canterbury/50/2000(H1N1)), complete sequence

gb|CY009184| Influenza A virus (A/Canterbury/30/2000(H1N1)), complete sequence

gb|CY009192| Influenza A virus (A/Canterbury/28/2000(H1N1)), complete sequence

gb|CY009200| Influenza A virus (A/Canterbury/36/2000(H1N1)), complete sequence

gb|CY009208| Influenza A virus (A/South Canterbury/35/2000(H1N1)), complete sequence

gb|CY009216| Influenza A virus (A/Canterbury/33/2000(H1N1)), complete sequence

gb|CY009224| Influenza A virus (A/Canterbury/32/2000(H1N1)), complete sequence

gb|CY009232| Influenza A virus (A/Canterbury/34/2000(H1N1)), complete sequence

gb|CY009544| Influenza A virus (A/Canterbury/5/2000(H1N1)), complete sequence

gb|CY009552| Influenza A virus (A/Canterbury/65/2000(H1N1)), complete sequence

gb|CY009760| Influenza A virus (A/Canterbury/23/2000(H1N1)), complete sequence

gb|CY009768| Influenza A virus (A/Canterbury/7/2000(H1N1)), complete sequence

gb|CY009776| Influenza A virus (A/South Canterbury/31/2000(H1N1)), complete sequence

gb|CY009784| Influenza A virus (A/South Canterbury/40/2000(H1N1)), complete sequence

gb|CY009792| Influenza A virus (A/Canterbury/76/2000(H1N1)), complete sequence

gb|CY009800| Influenza A virus (A/Canterbury/79/2000(H1N1)), complete sequence

gb|CY009808| Influenza A virus (A/Canterbury/87/2000(H1N1)), complete sequence

gb|CY009816| Influenza A virus (A/Canterbury/78/2000(H1N1)), complete sequence

gb|CY009824| Influenza A virus (A/Canterbury/60/2000(H1N1)), complete sequence

gb|CY009832| Influenza A virus (A/Canterbury/41/2000(H1N1)), complete sequence

gb|CY009840| Influenza A virus (A/South Canterbury/59/2000(H1N1)), complete sequence

gb|CY009848| Influenza A virus (A/Canterbury/57/2000(H1N1)), complete sequence

gb|CY009944| Influenza A virus (A/Canterbury/9/2000(H1N1)), complete sequence

gb|CY010096| Influenza A virus (A/Canterbury/43/2000(H1N1)), complete sequence

gb|CY010104| Influenza A virus (A/Canterbury/27/2000(H1N1)), complete sequence

gb|CY010112| Influenza A virus (A/Canterbury/51/2000(H1N1)), complete sequence

gb|CY010120| Influenza A virus (A/Canterbury/58/2000(H1N1)), complete sequence

gb|CY010128| Influenza A virus (A/Canterbury/54/2000(H1N1)), complete sequence

gb|CY010136| Influenza A virus (A/Canterbury/100/2000(H1N1)), complete sequence

gb|CY010144| Influenza A virus (A/Canterbury/95/2000(H1N1)), complete sequence

gb|CY010384| Influenza A virus (A/Canterbury/8/2000(H1N1)), complete sequence

gb|CY010392| Influenza A virus (A/Canterbury/63/2000(H1N1)), complete sequence

gb|CY011000| Influenza A virus (A/Wellington/2/2000(H1N1)), complete sequence

gb|CY011008| Influenza A virus (A/Wellington/3/2000(H1N1)), complete sequence

gb|CY011016| Influenza A virus (A/Wellington/5/2000(H1N1)), complete sequence

gb|CY011588| Influenza A virus (A/Dunedin/2/2000(H1N1)), complete sequence

gb|CY011780| Influenza A virus (A/Canterbury/48/2000(H1N1)), complete sequence

gb|CY011788| Influenza A virus (A/Canterbury/55/2000(H1N1)), complete sequence

gb|CY011956| Influenza A virus (A/Waikato/19/2000(H1N1)), complete sequence

gb|CY012300| Influenza A virus (A/Wellington/7/2000(H1N1)), complete sequence

gb|CY012604| Influenza A virus (A/Wellington/11/2000(H1N1)), complete sequence

gb|CY012612| Influenza A virus (A/Wellington/16/2000(H1N1)), complete sequence

gb|CY013036| Influenza A virus (A/Wellington/18/2000(H1N1)), complete sequence

gb|CY013044| Influenza A virus (A/Waikato/18/2000(H1N1)), complete sequence

gb|CY016200| Influenza A virus (A/Wellington/14/2000(H1N1)), complete sequence

gb|CY016975| Influenza A virus (A/Wellington/24/2000(H1N1)), complete sequence

gb|CY020513| Influenza A virus (A/Wellington/6/2000(H1N1)), complete sequence

gb|CY021633| Influenza A virus (A/Wellington/4/2000(H1N1)), complete sequence

gb|CY022153| Influenza A virus (A/Auckland/579/2000(H1N1)), complete sequence

gb|CY022161| Influenza A virus (A/Auckland/580/2000(H1N1)), complete sequence

gb|CY022169| Influenza A virus (A/Auckland/581/2000(H1N1)), complete sequence

gb|CY022177| Influenza A virus (A/Auckland/582/2000(H1N1)), complete sequence

gb|CY022489| Influenza A virus (A/Auckland/586/2000(H1N1)), complete sequence

gb|CY022497| Influenza A virus (A/Auckland/587/2000(H1N1)), complete sequence

gb|CY022513| Influenza A virus (A/Auckland/590/2000(H1N1)), complete sequence

gb|CY023014| Influenza A virus (A/Auckland/591/2000(H1N1)), complete sequence

gb|CY023022| Influenza A virus (A/Auckland/595/2000(H1N1)), complete sequence

gb|CY009864| Influenza A virus (A/Canterbury/08/2001(H1N1)), complete sequence

gb|CY009872| Influenza A virus (A/South Canterbury/15/2001(H1N1)), complete sequence

gb|CY009880| Influenza A virus (A/Canterbury/16/2001(H1N1)), complete sequence

gb|CY009888| Influenza A virus (A/Canterbury/41/2001(H1N1)), complete sequence

gb|CY009984| Influenza A virus (A/Canterbury/153/2001(H1N1)), complete sequence

gb|CY010152| Influenza A virus (A/Canterbury/17/2001(H1N1)), complete sequence

gb|CY010160| Influenza A virus (A/Canterbury/19/2001(H1N1)), complete sequence

gb|CY010168| Influenza A virus (A/Canterbury/21/2001(H1N1)), complete sequence

gb|CY010176| Influenza A virus (A/Canterbury/23/2001(H1N1)), complete sequence

gb|CY010184| Influenza A virus (A/Canterbury/24/2001(H1N1)), complete sequence

gb|CY010192| Influenza A virus (A/Canterbury/25/2001(H1N1)), complete sequence

gb|CY010200| Influenza A virus (A/Canterbury/27/2001(H1N1)), complete sequence

gb|CY010208| Influenza A virus (A/Canterbury/29/2001(H1N1)), complete sequence

gb|CY010216| Influenza A virus (A/Canterbury/30/2001(H1N1)), complete sequence

gb|CY010224| Influenza A virus (A/Canterbury/34/2001(H1N1)), complete sequence

gb|CY010232| Influenza A virus (A/Canterbury/35/2001(H1N1)), complete sequence

gb|CY010240| Influenza A virus (A/Canterbury/40/2001(H1N1)), complete sequence

gb|CY010248| Influenza A virus (A/Canterbury/45/2001(H1N1)), complete sequence

gb|CY010256| Influenza A virus (A/Canterbury/47/2001(H1N1)), complete sequence

gb|CY010264| Influenza A virus (A/Canterbury/48/2001(H1N1)), complete sequence

gb|CY010272| Influenza A virus (A/Canterbury/53/2001(H1N1)), complete sequence

gb|CY010280| Influenza A virus (A/Canterbury/58/2001(H1N1)), complete sequence

gb|CY010288| Influenza A virus (A/Canterbury/64/2001(H1N1)), complete sequence

gb|CY010296| Influenza A virus (A/Canterbury/72/2001(H1N1)), complete sequence

gb|CY010304| Influenza A virus (A/Canterbury/73/2001(H1N1)), complete sequence

gb|CY010312| Influenza A virus (A/Canterbury/119/2001(H1N1)), complete sequence

gb|CY010320| Influenza A virus (A/Canterbury/125/2001(H1N1)), complete sequence

gb|CY010328| Influenza A virus (A/Canterbury/126/2001(H1N1)), complete sequence

gb|CY010336| Influenza A virus (A/Canterbury/139/2001(H1N1)), complete sequence

gb|CY010344| Influenza A virus (A/Canterbury/144/2001(H1N1)), complete sequence

gb|CY010352| Influenza A virus (A/Canterbury/155/2001(H1N1)), complete sequence

gb|CY010360| Influenza A virus (A/South Canterbury/159/2001(H1N1)), complete sequence

gb|CY010400| Influenza A virus (A/Canterbury/01/2001(H1N1)), complete sequence

gb|CY010408| Influenza A virus (A/West Coast/33/2001(H1N1)), complete sequence

gb|CY010416| Influenza A virus (A/Canterbury/51/2001(H1N1)), complete sequence

gb|CY010424| Influenza A virus (A/Canterbury/63/2001(H1N1)), complete sequence

gb|CY010432| Influenza A virus (A/Canterbury/65/2001(H1N1)), complete sequence

gb|CY010440| Influenza A virus (A/Canterbury/68/2001(H1N1)), complete sequence

gb|CY010448| Influenza A virus (A/Canterbury/69/2001(H1N1)), complete sequence

gb|CY010456| Influenza A virus (A/Canterbury/70/2001(H1N1)), complete sequence

gb|CY010464| Influenza A virus (A/Canterbury/71/2001(H1N1)), complete sequence

gb|CY010472| Influenza A virus (A/Canterbury/79/2001(H1N1)), complete sequence

gb|CY010480| Influenza A virus (A/Canterbury/106/2001(H1N1)), complete sequence

gb|CY010560| Influenza A virus (A/Canterbury/54/2001(H1N1)), complete sequence

gb|CY010768| Influenza A virus (A/Canterbury/20/2001(H1N1)), complete sequence

gb|CY010776| Influenza A virus (A/Canterbury/22/2001(H1N1)), complete sequence

gb|CY010784| Influenza A virus (A/Canterbury/42/2001(H1N1)), complete sequence

gb|CY011076| Influenza A virus (A/West Coast/31/2001(H1N1)), complete sequence

gb|CY011084| Influenza A virus (A/Canterbury/60/2001(H1N1)), complete sequence

gb|CY011092| Influenza A virus (A/Canterbury/152/2001(H1N1)), complete sequence

gb|CY011156| Influenza A virus (A/Wellington/1/2001(H1N1)), complete sequence

gb|CY011164| Influenza A virus (A/Waikato/4/2001(H1N1)), complete sequence

gb|CY011172| Influenza A virus (A/Waikato/7/2001(H1N1)), complete sequence

gb|CY011180| Influenza A virus (A/Wellington/3/2001(H1N1)), complete sequence

gb|CY011188| Influenza A virus (A/Waikato/10/2001(H1N1)), complete sequence

gb|CY011196| Influenza A virus (A/Waikato/16/2001(H1N1)), complete sequence

gb|CY011204| Influenza A virus (A/Waikato/20/2001(H1N1)), complete sequence

gb|CY011212| Influenza A virus (A/Waikato/18/2001(H1N1)), complete sequence

gb|CY011220| Influenza A virus (A/Wellington/9/2001(H1N1)), complete sequence

gb|CY011228| Influenza A virus (A/Waikato/51/2001(H1N1)), complete sequence

gb|CY011236| Influenza A virus (A/Waikato/42/2001(H1N1)), complete sequence

gb|CY011244| Influenza A virus (A/Wellington/28/2001(H1N1)), complete sequence

gb|CY011396| Influenza A virus (A/Waikato/80/2001(H1N1)), complete sequence

gb|CY011412| Influenza A virus (A/Canterbury/26/2001(H1N1)), complete sequence

gb|CY011604| Influenza A virus (A/Waikato/3/2001(H1N1)), complete sequence

gb|CY011612| Influenza A virus (A/Wellington/32/2001(H1N1)), complete sequence

gb|CY012308| Influenza A virus (A/Wellington/17/2001(H1N1)), complete sequence

gb|CY016439| Influenza A virus (A/Waikato/92/2001(H1N1)), complete sequence

gb|CY019209| Influenza A virus (A/Canterbury/05/2001(H1N1)), complete sequence

gb|CY022537| Influenza A virus (A/Auckland/605/2001(H1N1)), complete sequence

gb|CY022545| Influenza A virus (A/Auckland/606/2001(H1N1)), complete sequence

gb|CY007471| Influenza A virus (A/Canterbury/106/2004(H1N1)), complete sequence

gb|CY013561| Influenza A virus (A/Wellington/10/2005(H1N1)), complete sequence

gb|CY013569| Influenza A virus (A/Wellington/12/2005(H1N1)), complete sequence

gb|CY013577| Influenza A virus (A/Otago/5/2005(H1N1)), complete sequence

gb|CY013585| Influenza A virus (A/Waikato/13/2005(H1N1)), complete sequence

gb|CY013593| Influenza A virus (A/Wellington/13/2005(H1N1)), complete sequence

gb|CY013601| Influenza A virus (A/Waikato/14/2005(H1N1)), complete sequence

gb|CY014011| Influenza A virus (A/Wellington/11/2005(H1N1)), complete sequence

gb|CY015584| Influenza A virus (A/Wellington/14/2005(H1N1)), complete sequence

gb|CY016463| Influenza A virus (A/Waikato/11/2005(H1N1)), complete sequence

gb|CY017319| Influenza A virus (A/Waikato/4/2005(H1N1)), complete sequence

gb|CY020001| Influenza A virus (A/Waikato/17/2005(H1N1)), complete sequence

gb|CY022585| Influenza A virus (A/Auckland/619/2005(H1N1)), complete sequence

gb|CY010376| Influenza A virus (A/USSR/90/77(H1N1)), complete sequence

gb|DQ508901| Influenza A virus (A/USSR/90/1977(H1N1)), complete sequence

gb|CY009288| Influenza A virus (A/USSR/92/77(H1N1)), complete sequence

gb|CY021913| Influenza A virus (A/USSR/46/1979(H1N1)), complete sequence

gb|CY020481| Influenza A virus (A/Singapore/6/1986(H1N1)), complete sequence

gb|DQ508877| Influenza A virus (A/Taiwan/01/1986(H1N1)), complete sequence

gb|DQ415360| Influenza A virus (A/TW/130/96(H1N1)), complete sequence

gb|DQ415361| Influenza A virus (A/TW/3355/97(H1N1)), complete sequence

gb|CY020473| Influenza A virus (A/Phila/1935(H1N1)), complete sequence

gb|CY013275| Influenza A virus (A/Hickox/1940(H1N1)), complete sequence

gb|CY009280| Influenza A virus (A/Bel/1942(H1N1)), complete sequence

gb|CY020289| Influenza A virus (A/AA/Marton/1943(H1N1)), complete sequence

gb|CY020465| Influenza A virus (A/Iowa/1943(H1N1)), complete sequence

gb|CY021713| Influenza A virus (A/AA/Huston/1945(H1N1)), complete sequence

gb|CY009616| Influenza A virus (A/FortMonmouth/1/47(H1N1)), complete sequence

gb|CY019951| Influenza A virus (A/Albany/4835/1948(H1N1)), complete sequence

gb|CY009336| Influenza A virus (A/Fort Worth/50(H1N1)), complete sequence

gb|CY021705| Influenza A virus (A/Albany/4836/1950(H1N1)), complete sequence

gb|CY021825| Influenza A virus (A/Albany/12/1951(H1N1)), complete sequence

gb|CY021905| Influenza A virus (A/Albany/1618/1951(H1N1)), complete sequence

gb|CY022025| Influenza A virus (A/Albany/14/1951(H1N1)), complete sequence

gb|CY022097| Influenza A virus (A/Albany/13/1951(H1N1)), complete sequence

gb|CY008992| Influenza A virus (A/Denver/57(H1N1)), complete sequence

gb|CY010872| Influenza A virus (A/Memphis/10/1978(H1N1)), complete sequence

gb|CY010880| Influenza A virus (A/Memphis/11/1978(H1N1)), complete sequence

gb|CY010888| Influenza A virus (A/Memphis/13/1978(H1N1)), complete sequence

gb|CY010896| Influenza A virus (A/Memphis/15/1978(H1N1)), complete sequence

gb|CY010904| Influenza A virus (A/Memphis/17/1978(H1N1)), complete sequence

gb|CY011300| Influenza A virus (A/Memphis/1/1978(H1N1)), complete sequence

gb|CY017367| Influenza A virus (A/Memphis/20/1978(H1N1)), complete sequence

gb|CY019967| Influenza A virus (A/Arizona/14/1978(H1N1)), complete sequence

gb|CY020169| Influenza A virus (A/Lackland/3/1978(H1N1)), complete sequence

gb|CY020177| Influenza A virus (A/Lackland/7/1978(H1N1)), complete sequence

gb|CY021801| Influenza A virus (A/Albany/20/1978(H1N1)), complete sequence

gb|CY026415| Influenza A virus (A/Albany/8/1979(H1N1)), complete sequence

gb|CY019743| Influenza A virus (A/Memphis/1/1979(H1N1)), complete sequence

gb|CY010912| Influenza A virus (A/Memphis/7/1980(H1N1)), complete sequence

gb|CY020185| Influenza A virus (A/Maryland/2/1980(H1N1)), complete sequence

gb|CY021033| Influenza A virus (A/Baylor/4052/1981(H1N1)), complete sequence

gb|CY009624| Influenza A virus (A/Baylor/11735/82(H1N1)), complete sequence

gb|CY010368| Influenza A virus (A/Baylor/11515/82(H1N1)), complete sequence

gb|CY027535| Influenza A virus (A/Memphis/44/1983(H1N1)), complete sequence

gb|CY010920| Influenza A virus (A/Memphis/3/1983(H1N1)), complete sequence

gb|CY010928| Influenza A virus (A/Memphis/4/1983(H1N1)), complete sequence

gb|CY010936| Influenza A virus (A/Memphis/7/1983(H1N1)), complete sequence

gb|CY010944| Influenza A virus (A/Memphis/8/1983(H1N1)), complete sequence

gb|CY010952| Influenza A virus (A/Memphis/12/1983(H1N1)), complete sequence

gb|CY010960| Influenza A virus (A/Memphis/15/1983(H1N1)), complete sequence

gb|CY010968| Influenza A virus (A/Memphis/16/1983(H1N1)), complete sequence

gb|CY010976| Influenza A virus (A/Memphis/17/1983(H1N1)), complete sequence

gb|CY010984| Influenza A virus (A/Memphis/18/1983(H1N1)), complete sequence

gb|CY011308| Influenza A virus (A/Memphis/11/1983(H1N1)), complete sequence

gb|CY011316| Influenza A virus (A/Memphis/13/1983(H1N1)), complete sequence

gb|CY012444| Influenza A virus (A/Memphis/19/1983(H1N1)), complete sequence

gb|CY012884| Influenza A virus (A/Memphis/1/1983(H1N1)), complete sequence

gb|CY012892| Influenza A virus (A/Memphis/2/1983(H1N1)), complete sequence

gb|CY013299| Influenza A virus (A/Memphis/14/1983(H1N1)), complete sequence

gb|CY013883| Influenza A virus (A/Memphis/10/1983(H1N1)), complete sequence

gb|CY015528| Influenza A virus (A/Memphis/6/1983(H1N1)), complete sequence

gb|CY017199| Influenza A virus (A/Memphis/20/1983(H1N1)), complete sequence

gb|CY017207| Influenza A virus (A/Memphis/23/1983(H1N1)), complete sequence

gb|CY017215| Influenza A virus (A/Memphis/24/1983(H1N1)), complete sequence

gb|CY017223| Influenza A virus (A/Memphis/27/1983(H1N1)), complete sequence

gb|CY017231| Influenza A virus (A/Memphis/28/1983(H1N1)), complete sequence

gb|CY017239| Influenza A virus (A/Memphis/29/1983(H1N1)), complete sequence

gb|CY017247| Influenza A virus (A/Memphis/30/1983(H1N1)), complete sequence

gb|CY017255| Influenza A virus (A/Memphis/32/1983(H1N1)), complete sequence

gb|CY017423| Influenza A virus (A/Memphis/21/1983(H1N1)), complete sequence

gb|CY017431| Influenza A virus (A/Memphis/22/1983(H1N1)), complete sequence

gb|CY017439| Influenza A virus (A/Memphis/26/1983(H1N1)), complete sequence

gb|CY017873| Influenza A virus (A/Memphis/25/1983(H1N1)), complete sequence

gb|CY017881| Influenza A virus (A/Memphis/35/1983(H1N1)), complete sequence

gb|CY019041| Influenza A virus (A/Memphis/38/1983(H1N1)), complete sequence

gb|CY019049| Influenza A virus (A/Memphis/40/1983(H1N1)), complete sequence

gb|CY019057| Influenza A virus (A/Memphis/41/1983(H1N1)), complete sequence

gb|CY019065| Influenza A virus (A/Memphis/42/1983(H1N1)), complete sequence

gb|CY019073| Influenza A virus (A/Memphis/46/1983(H1N1)), complete sequence

gb|CY019081| Influenza A virus (A/Memphis/48/1983(H1N1)), complete sequence

gb|CY019089| Influenza A virus (A/Memphis/50/1983(H1N1)), complete sequence

gb|CY019097| Influenza A virus (A/Memphis/51/1983(H1N1)), complete sequence

gb|CY019225| Influenza A virus (A/Memphis/31/1983(H1N1)), complete sequence

gb|CY019233| Influenza A virus (A/Memphis/47/1983(H1N1)), complete sequence

gb|CY019241| Influenza A virus (A/Memphis/53/1983(H1N1)), complete sequence

gb|CY019759| Influenza A virus (A/Memphis/49/1983(H1N1)), complete sequence

gb|CY019767| Influenza A virus (A/Memphis/54/1983(H1N1)), complete sequence

gb|CY020241| Influenza A virus (A/Memphis/39/1983(H1N1)), complete sequence

gb|CY021729| Influenza A virus (A/Memphis/1/1984(H1N1)), complete sequence

gb|CY019105| Influenza A virus (A/Memphis/12/1986(H1N1)), complete sequence

gb|CY020569| Influenza A virus (A/Texas/2922-3/1986(H1N1)), complete sequence

gb|CY021737| Influenza A virus (A/New York/2924-1/1986(H1N1)), complete sequence

gb|CY019775| Influenza A virus (A/Memphis/3/1987(H1N1)), complete sequence

gb|CY019783| Influenza A virus (A/Memphis/4/1987(H1N1)), complete sequence

gb|CY021977| Influenza A virus (A/Memphis/1/1987(H1N1)), complete sequence

gb|DQ508893| Influenza A virus (A/Texas/36/1991(H1N1)), complete sequence

gb|CY009320| Influenza A virus (A/Texas/36/91(H1N1)), complete sequence

gb|CY010488| Influenza A virus (A/New York/604/1995(H1N1)), complete sequence

gb|CY010496| Influenza A virus (A/New York/605/1995(H1N1)), complete sequence

gb|CY010504| Influenza A virus (A/New York/607/1995(H1N1)), complete sequence

gb|CY010512| Influenza A virus (A/New York/615/1995(H1N1)), complete sequence

gb|CY010528| Influenza A virus (A/New York/620/1995(H1N1)), complete sequence

gb|CY010536| Influenza A virus (A/New York/621/1995(H1N1)), complete sequence

gb|CY010544| Influenza A virus (A/New York/627/1995(H1N1)), complete sequence

gb|CY010808| Influenza A virus (A/New York/614/1995(H1N1)), complete sequence

gb|CY010824| Influenza A virus (A/New York/638/1995(H1N1)), complete sequence

gb|CY010832| Influenza A virus (A/New York/651/1995(H1N1)), complete sequence

gb|CY011276| Influenza A virus (A/New York/645/1995(H1N1)), complete sequence

gb|CY011284| Influenza A virus (A/New York/649/1995(H1N1)), complete sequence

gb|CY011804| Influenza A virus (A/New York/629/1995(H1N1)), complete sequence

gb|CY012860| Influenza A virus (A/New York/630/1995(H1N1)), complete sequence

gb|CY012868| Influenza A virus (A/New York/642/1995(H1N1)), complete sequence

gb|CY012876| Influenza A virus (A/New York/650/1995(H1N1)), complete sequence

gb|CY013283| Influenza A virus (A/New York/643/1995(H1N1)), complete sequence

gb|CY013307| Influenza A virus (A/New York/656/1995(H1N1)), complete sequence

gb|CY013875| Influenza A virus (A/New York/616/1995(H1N1)), complete sequence

gb|CY015536| Influenza A virus (A/New York/694/1995(H1N1)), complete sequence

gb|CY016967| Influenza A virus (A/New York/633/1995(H1N1)), complete sequence

gb|CY010840| Influenza A virus (A/New York/653/1996(H1N1)), complete sequence

gb|CY010848| Influenza A virus (A/New York/640/1996(H1N1)), complete sequence

gb|CY011796| Influenza A virus (A/New York/626/1996(H1N1)), complete sequence

gb|CY013291| Influenza A virus (A/New York/646/1996(H1N1)), complete sequence

gb|CY019113| Influenza A virus (A/Memphis/2/1996(H1N1)), complete sequence

gb|CY019121| Influenza A virus (A/Memphis/3/1996(H1N1)), complete sequence

gb|CY019129| Influenza A virus (A/Memphis/5/1996(H1N1)), complete sequence

gb|CY019137| Influenza A virus (A/Memphis/7/1996(H1N1)), complete sequence

gb|CY019791| Influenza A virus (A/Memphis/6/1996(H1N1)), complete sequence

gb|CY019799| Influenza A virus (A/Memphis/10/1996(H1N1)), complete sequence

gb|CY019807| Influenza A virus (A/Memphis/11/1996(H1N1)), complete sequence

gb|CY019871| Influenza A virus (A/Memphis/13/1996(H1N1)), complete sequence

gb|CY020257| Influenza A virus (A/Memphis/14/1996(H1N1)), complete sequence

gb|CY021009| Influenza A virus (A/Memphis/15/1996(H1N1)), complete sequence

gb|CY000453| Influenza A virus (A/New York/146/2000(H1N1)), complete sequence

gb|CY002652| Influenza A virus (A/New York/234/2000(H1N1)), complete sequence

gb|CY021697| Influenza A virus (A/Memphis/15/2000(H1N1)), complete sequence

gb|CY001956| Influenza A virus (A/New York/205/2001(H1N1)), complete sequence

gb|CY002396| Influenza A virus (A/New York/343/2001(H1N1)), complete sequence

gb|CY002404| Influenza A virus (A/New York/345/2001(H1N1)), complete sequence

gb|CY002572| Influenza A virus (A/New York/281/2001(H1N1)), complete sequence

gb|CY002676| Influenza A virus (A/New York/310/2001(H1N1)), complete sequence

gb|CY002700| Influenza A virus (A/New York/312/2001(H1N1)), complete sequence

gb|CY002804| Influenza A virus (A/New York/308/2001(H1N1)), complete sequence

gb|CY003004| Influenza A virus (A/New York/239/2001(H1N1)), complete sequence

gb|CY003012| Influenza A virus (A/New York/241/2001(H1N1)), complete sequence

gb|CY003020| Influenza A virus (A/New York/246/2001(H1N1)), complete sequence

gb|CY003028| Influenza A virus (A/New York/341/2001(H1N1)), complete sequence

gb|CY003292| Influenza A virus (A/New York/444/2001(H1N1)), complete sequence

gb|CY003316| Influenza A virus (A/New York/302/2001(H1N1)), complete sequence

gb|CY003324| Influenza A virus (A/New York/342/2001(H1N1)), complete sequence

gb|CY003332| Influenza A virus (A/New York/346/2001(H1N1)), complete sequence

gb|CY003396| Influenza A virus (A/New York/305/2001(H1N1)), complete sequence

gb|CY003404| Influenza A virus (A/New York/306/2001(H1N1)), complete sequence

gb|CY003468| Influenza A virus (A/New York/442/2001(H1N1)), complete sequence

gb|CY003476| Influenza A virus (A/New York/443/2001(H1N1)), complete sequence

gb|CY003484| Influenza A virus (A/New York/446/2001(H1N1)), complete sequence

gb|CY003837| Influenza A virus (A/New York/445/2001(H1N1)), complete sequence

gb|CY006359| Influenza A virus (A/New York/242/2001(H1N1)), complete sequence

gb|CY006367| Influenza A virus (A/New York/303/2001(H1N1)), complete sequence

gb|CY006423| Influenza A virus (A/New York/212/2001(H1N1)), complete sequence

gb|CY006783| Influenza A virus (A/New York/344/2001(H1N1)), complete sequence

gb|CY006879| Influenza A virus (A/New York/309/2001(H1N1)), complete sequence

gb|CY008152| Influenza A virus (A/New York/307/2001(H1N1)), complete sequence

gb|CY009240| Influenza A virus (A/New York/441/2001(H1N1)), complete sequence

gb|CY010856| Influenza A virus (A/New York/235/2001(H1N1)), complete sequence

gb|CY019879| Influenza A virus (A/Memphis/8/2001(H1N1)), complete sequence

gb|CY020145| Influenza A virus (A/Memphis/6/2001(H1N1)), complete sequence

gb|CY020153| Influenza A virus (A/Memphis/7/2001(H1N1)), complete sequence

gb|CY020265| Influenza A virus (A/Memphis/1/2001(H1N1)), complete sequence

gb|CY002532| Influenza A virus (A/New York/220/2002(H1N1)), complete sequence

gb|CY003308| Influenza A virus (A/New York/291/2002(H1N1)), complete sequence

gb|CY006679| Influenza A virus (A/New York/494/2002(H1N1)), complete sequence

gb|CY002540| Influenza A virus (A/New York/227/2003(H1N1)), complete sequence

gb|CY002628| Influenza A virus (A/New York/230/2003(H1N1)), complete sequence

gb|CY002684| Influenza A virus (A/New York/222/2003(H1N1)), complete sequence

gb|CY002692| Influenza A virus (A/New York/223/2003(H1N1)), complete sequence

gb|CY002708| Influenza A virus (A/New York/348/2003(H1N1)), complete sequence

gb|CY002812| Influenza A virus (A/New York/399/2003(H1N1)), complete sequence

gb|CY002988| Influenza A virus (A/New York/221/2003(H1N1)), complete sequence

gb|CY003300| Influenza A virus (A/New York/228/2003(H1N1)), complete sequence

gb|CY003380| Influenza A virus (A/New York/292/2003(H1N1)), complete sequence

gb|CY003388| Influenza A virus (A/New York/293/2003(H1N1)), complete sequence

gb|CY003692| Influenza A virus (A/New York/486/2003(H1N1)), complete sequence

gb|CY003708| Influenza A virus (A/New York/496/2003(H1N1)), complete sequence

gb|CY006199| Influenza A virus (A/New York/497/2003(H1N1)), complete sequence

gb|CY006431| Influenza A virus (A/New York/350/2003(H1N1)), complete sequence

gb|CY006671| Influenza A virus (A/New York/493/2003(H1N1)), complete sequence

gb|CY006919| Influenza A virus (A/New York/488/2003(H1N1)), complete sequence

gb|CY008528| Influenza A virus (A/New York/483/2003(H1N1)), complete sequence

gb|CY009000| Influenza A virus (A/New York/484/2003(H1N1)), complete sequence

gb|CY019345| Influenza A virus (A/Memphis/6/2003(H1N1)), complete sequence

gb|CY019887| Influenza A virus (A/Memphis/5/2003(H1N1)), complete sequence

gb|DQ889685| Influenza A virus (A/Iowa/CEID23/2005(H1N1)), complete sequence

gb|CY028199| Influenza A virus (A/Kentucky/UR06-0007/2006(H1N1)), complete sequence

gb|CY025233| Influenza A virus (A/Kentucky/UR06-0010/2006(H1N1)), complete sequence

gb|CY025225| Influenza A virus (A/Michigan/UR06-0015/2006(H1N1)), complete sequence

gb|CY027879| Influenza A virus (A/Mississippi/UR06-0014/2006(H1N1)), complete sequence

gb|CY027151| Influenza A virus (A/North Carolina/UR06-0011/2006(H1N1)), complete sequence

gb|CY025217| Influenza A virus (A/Texas/UR06-0012/2006(H1N1)), complete sequence

gb|CY017375| Influenza A virus (A/New York/8/2006(H1N1)), complete sequence

gb|CY025385| Influenza A virus (A/Alabama/UR06-0455/2007(H1N1)), complete sequence

gb|CY027415| Influenza A virus (A/Alabama/UR06-0536/2007(H1N1)), complete sequence

gb|CY027847| Influenza A virus (A/California/UR06-0125/2007(H1N1)), complete sequence

gb|CY026359| Influenza A virus (A/California/UR06-0232/2007(H1N1)), complete sequence

gb|CY026535| Influenza A virus (A/California/UR06-0302/2007(H1N1)), complete sequence

gb|CY026767| Influenza A virus (A/California/UR06-0321/2007(H1N1)), complete sequence

gb|CY026319| Influenza A virus (A/California/UR06-0374/2007(H1N1)), complete sequence

gb|CY026679| Influenza A virus (A/California/UR06-0375/2007(H1N1)), complete sequence

gb|CY026543| Influenza A virus (A/California/UR06-0393/2007(H1N1)), complete sequence

gb|CY025377| Influenza A virus (A/California/UR06-0435/2007(H1N1)), complete sequence

gb|CY027895| Influenza A virus (A/California/UR06-0440/2007(H1N1)), complete sequence

gb|CY028463| Influenza A virus (A/California/UR06-0442/2007(H1N1)), complete sequence

gb|CY028311| Influenza A virus (A/California/UR06-0479/2007(H1N1)), complete sequence

gb|CY028471| Influenza A virus (A/California/UR06-0552/2007(H1N1)), complete sequence

gb|CY027359| Influenza A virus (A/California/UR06-0564/2007(H1N1)), complete sequence

gb|CY027215| Influenza A virus (A/California/UR06-0585/2007(H1N1)), complete sequence

gb|CY026527| Influenza A virus (A/Colorado/UR06-0053/2007(H1N1)), complete sequence

gb|CY026583| Influenza A virus (A/Colorado/UR06-0110/2007(H1N1)), complete sequence

gb|CY025831| Influenza A virus (A/Colorado/UR06-0111/2007(H1N1)), complete sequence

gb|CY025409| Influenza A virus (A/Colorado/UR06-0207/2007(H1N1)), complete sequence

gb|CY025337| Influenza A virus (A/Colorado/UR06-0255/2007(H1N1)), complete sequence

gb|CY026783| Influenza A virus (A/Colorado/UR06-0287/2007(H1N1)), complete sequence

gb|CY026823| Influenza A virus (A/Colorado/UR06-0336/2007(H1N1)), complete sequence

gb|CY027903| Influenza A virus (A/Colorado/UR06-0496/2007(H1N1)), complete sequence

gb|CY026239| Influenza A virus (A/Colorado/UR06-0498/2007(H1N1)), complete sequence

gb|CY027231| Influenza A virus (A/Colorado/UR06-0499/2007(H1N1)), complete sequence

gb|CY027103| Influenza A virus (A/Florida/UR06-0049/2007(H1N1)), complete sequence

gb|CY027319| Influenza A virus (A/Florida/UR06-0208/2007(H1N1)), complete sequence

gb|CY028343| Influenza A virus (A/Florida/UR06-0209/2007(H1N1)), complete sequence

gb|CY025775| Influenza A virus (A/Florida/UR06-0280/2007(H1N1)), complete sequence

gb|CY026327| Influenza A virus (A/Florida/UR06-0354/2007(H1N1)), complete sequence

gb|CY025537| Influenza A virus (A/Florida/UR06-0355/2007(H1N1)), complete sequence

gb|CY025783| Influenza A virus (A/Florida/UR06-0383/2007(H1N1)), complete sequence

gb|CY026727| Influenza A virus (A/Florida/UR06-0412/2007(H1N1)), complete sequence

gb|CY027615| Influenza A virus (A/Florida/UR06-0447/2007(H1N1)), complete sequence

gb|CY026663| Influenza A virus (A/Florida/UR06-0501/2007(H1N1)), complete sequence

gb|CY026551| Influenza A virus (A/Florida/UR06-0577/2007(H1N1)), complete sequence

gb|CY027631| Influenza A virus (A/Florida/UR06-0578/2007(H1N1)), complete sequence

gb|CY025249| Influenza A virus (A/Illinois/UR006-018/2007(H1N1)), complete sequence

gb|CY025241| Influenza A virus (A/Illinois/UR06-0019/2007(H1N1)), complete sequence

gb|CY027807| Influenza A virus (A/Illinois/UR06-0032/2007(H1N1)), complete sequence

gb|CY027823| Influenza A virus (A/Illinois/UR06-0074/2007(H1N1)), complete sequence

gb|CY027671| Influenza A virus (A/Illinois/UR06-0088/2007(H1N1)), complete sequence

gb|CY028071| Influenza A virus (A/Illinois/UR06-0093/2007(H1N1)), complete sequence

gb|CY027439| Influenza A virus (A/Illinois/UR06-0094/2007(H1N1)), complete sequence

gb|CY026975| Influenza A virus (A/Illinois/UR06-0095/2007(H1N1)), complete sequence

gb|CY028351| Influenza A virus (A/Illinois/UR06-0096/2007(H1N1)), complete sequence

gb|CY027367| Influenza A virus (A/Illinois/UR06-0098/2007(H1N1)), complete sequence

gb|CY027487| Influenza A virus (A/Illinois/UR06-0115/2007(H1N1)), complete sequence

gb|CY027279| Influenza A virus (A/Illinois/UR06-0116/2007(H1N1)), complete sequence

gb|CY027703| Influenza A virus (A/Illinois/UR06-0131/2007(H1N1)), complete sequence

gb|CY027399| Influenza A virus (A/Illinois/UR06-0136/2007(H1N1)), complete sequence

gb|CY027095| Influenza A virus (A/Illinois/UR06-0137/2007(H1N1)), complete sequence

gb|CY027047| Influenza A virus (A/Illinois/UR06-0146/2007(H1N1)), complete sequence

gb|CY028423| Influenza A virus (A/Illinois/UR06-0215/2007(H1N1)), complete sequence

gb|CY026183| Influenza A virus (A/Illinois/UR06-0223/2007(H1N1)), complete sequence

gb|CY026695| Influenza A virus (A/Illinois/UR06-0224/2007(H1N1)), complete sequence

gb|CY027791| Influenza A virus (A/Illinois/UR06-0227/2007(H1N1)), complete sequence

gb|CY025575| Influenza A virus (A/Illinois/UR06-0248/2007(H1N1)), complete sequence

gb|CY027351| Influenza A virus (A/Illinois/UR06-0249/2007(H1N1)), complete sequence

gb|CY025441| Influenza A virus (A/Illinois/UR06-0333/2007(H1N1)), complete sequence

gb|CY026615| Influenza A virus (A/Illinois/UR06-0376/2007(H1N1)), complete sequence

gb|CY025329| Influenza A virus (A/Illinois/UR06-0377/2007(H1N1)), complete sequence

gb|CY025599| Influenza A virus (A/Illinois/UR06-0415/2007(H1N1)), complete sequence

gb|CY025521| Influenza A virus (A/Illinois/UR06-0456/2007(H1N1)), complete sequence

gb|CY027911| Influenza A virus (A/Illinois/UR06-0475/2007(H1N1)), complete sequence

gb|CY026799| Influenza A virus (A/Illinois/UR06-0491/2007(H1N1)), complete sequence

gb|CY027743| Influenza A virus (A/Kansas/UR06-0068/2007(H1N1)), complete sequence

gb|CY028015| Influenza A virus (A/Kansas/UR06-0084/2007(H1N1)), complete sequence

gb|CY027839| Influenza A virus (A/Kansas/UR06-0085/2007(H1N1)), complete sequence

gb|CY027039| Influenza A virus (A/Kansas/UR06-0104/2007(H1N1)), complete sequence

gb|CY025967| Influenza A virus (A/Kansas/UR06-0140/2007(H1N1)), complete sequence

gb|CY027855| Influenza A virus (A/Kansas/UR06-0143/2007(H1N1)), complete sequence

gb|CY028143| Influenza A virus (A/Kansas/UR06-0191/2007(H1N1)), complete sequence

gb|CY028367| Influenza A virus (A/Kansas/UR06-0192/2007(H1N1)), complete sequence

gb|CY027431| Influenza A virus (A/Kansas/UR06-0283/2007(H1N1)), complete sequence

gb|CY027983| Influenza A virus (A/Kansas/UR06-0284/2007(H1N1)), complete sequence

gb|CY026655| Influenza A virus (A/Kentucky/UR06-0027/2007(H1N1)), complete sequence

gb|CY026223| Influenza A virus (A/Kentucky/UR06-0028/2007(H1N1)), complete sequence

gb|CY027223| Influenza A virus (A/Kentucky/UR06-0029/2007(H1N1)), complete sequence

gb|CY025791| Influenza A virus (A/Kentucky/UR06-0033/2007(H1N1)), complete sequence

gb|CY025927| Influenza A virus (A/Kentucky/UR06-0034/2007(H1N1)), complete sequence

gb|CY025497| Influenza A virus (A/Kentucky/UR06-0042/2007(H1N1)), complete sequence

gb|CY025703| Influenza A virus (A/Kentucky/UR06-0043/2007(H1N1)), complete sequence

gb|CY028039| Influenza A virus (A/Kentucky/UR06-0046/2007(H1N1)), complete sequence

gb|CY026519| Influenza A virus (A/Kentucky/UR06-0057/2007(H1N1)), complete sequence

gb|CY026807| Influenza A virus (A/Kentucky/UR06-0058/2007(H1N1)), complete sequence

gb|CY025265| Influenza A virus (A/Kentucky/UR06-0059/2007(H1N1)), complete sequence

gb|CY026743| Influenza A virus (A/Kentucky/UR06-0061/2007(H1N1)), complete sequence

gb|CY025457| Influenza A virus (A/Kentucky/UR06-0062/2007(H1N1)), complete sequence

gb|CY025919| Influenza A virus (A/Kentucky/UR06-0069/2007(H1N1)), complete sequence

gb|CY026815| Influenza A virus (A/Kentucky/UR06-0071/2007(H1N1)), complete sequence

gb|CY025433| Influenza A virus (A/Kentucky/UR06-0072/2007(H1N1)), complete sequence

gb|CY027175| Influenza A virus (A/Kentucky/UR06-0081/2007(H1N1)), complete sequence

gb|CY025313| Influenza A virus (A/Kentucky/UR06-0082/2007(H1N1)), complete sequence

gb|CY025513| Influenza A virus (A/Kentucky/UR06-0097/2007(H1N1)), complete sequence

gb|CY026879| Influenza A virus (A/Kentucky/UR06-0123/2007(H1N1)), complete sequence

gb|CY026871| Influenza A virus (A/Kentucky/UR06-0127/2007(H1N1)), complete sequence

gb|CY025983| Influenza A virus (A/Kentucky/UR06-0128/2007(H1N1)), complete sequence

gb|CY025465| Influenza A virus (A/Kentucky/UR06-0129/2007(H1N1)), complete sequence

gb|CY027055| Influenza A virus (A/Kentucky/UR06-0154/2007(H1N1)), complete sequence

gb|CY026983| Influenza A virus (A/Kentucky/UR06-0161/2007(H1N1)), complete sequence

gb|CY028023| Influenza A virus (A/Kentucky/UR06-0162/2007(H1N1)), complete sequence

gb|CY028359| Influenza A virus (A/Kentucky/UR06-0181/2007(H1N1)), complete sequence

gb|CY028151| Influenza A virus (A/Kentucky/UR06-0182/2007(H1N1)), complete sequence

gb|CY027023| Influenza A virus (A/Kentucky/UR06-0183/2007(H1N1)), complete sequence

gb|CY027463| Influenza A virus (A/Kentucky/UR06-0184/2007(H1N1)), complete sequence

gb|CY026207| Influenza A virus (A/Kentucky/UR06-0187/2007(H1N1)), complete sequence

gb|CY025639| Influenza A virus (A/Kentucky/UR06-0188/2007(H1N1)), complete sequence

gb|CY027335| Influenza A virus (A/Kentucky/UR06-0220/2007(H1N1)), complete sequence

gb|CY025679| Influenza A virus (A/Kentucky/UR06-0240/2007(H1N1)), complete sequence

gb|CY025951| Influenza A virus (A/Kentucky/UR06-0257/2007(H1N1)), complete sequence

gb|CY028167| Influenza A virus (A/Kentucky/UR06-0258/2007(H1N1)), complete sequence

gb|CY027919| Influenza A virus (A/Kentucky/UR06-0259/2007(H1N1)), complete sequence

gb|CY027015| Influenza A virus (A/Kentucky/UR06-0327/2007(H1N1)), complete sequence

gb|CY026991| Influenza A virus (A/Kentucky/UR06-0328/2007(H1N1)), complete sequence

gb|CY027783| Influenza A virus (A/Kentucky/UR06-0339/2007(H1N1)), complete sequence

gb|CY025361| Influenza A virus (A/Kentucky/UR06-0363/2007(H1N1)), complete sequence

gb|CY027287| Influenza A virus (A/Kentucky/UR06-0371/2007(H1N1)), complete sequence

gb|CY028087| Influenza A virus (A/Kentucky/UR06-0391/2007(H1N1)), complete sequence

gb|CY028047| Influenza A virus (A/Kentucky/UR06-0401/2007(H1N1)), complete sequence

gb|CY027375| Influenza A virus (A/Kentucky/UR06-0424/2007(H1N1)), complete sequence

gb|CY028055| Influenza A virus (A/Kentucky/UR06-0425/2007(H1N1)), complete sequence

gb|CY027679| Influenza A virus (A/Kentucky/UR06-0449/2007(H1N1)), complete sequence

gb|CY026591| Influenza A virus (A/Kentucky/UR06-0476/2007(H1N1)), complete sequence

gb|CY026231| Influenza A virus (A/Kentucky/UR06-0538/2007(H1N1)), complete sequence

gb|CY025943| Influenza A virus (A/Kentucky/UR06-0539/2007(H1N1)), complete sequence

gb|CY025671| Influenza A virus (A/Kentucky/UR06-0553/2007(H1N1)), complete sequence

gb|CY027207| Influenza A virus (A/Mississippi/UR06-0047/2007(H1N1)), complete sequence

gb|CY025807| Influenza A virus (A/Mississippi/UR06-0048/2007(H1N1)), complete sequence

gb|CY025631| Influenza A virus (A/Mississippi/UR06-0086/2007(H1N1)), complete sequence

gb|CY025559| Influenza A virus (A/Mississippi/UR06-0130/2007(H1N1)), complete sequence

gb|CY025623| Influenza A virus (A/Mississippi/UR06-0142/2007(H1N1)), complete sequence

gb|CY025583| Influenza A virus (A/Mississippi/UR06-0145/2007(H1N1)), complete sequence

gb|CY025321| Influenza A virus (A/Mississippi/UR06-0242/2007(H1N1)), complete sequence

gb|CY026375| Influenza A virus (A/Mississippi/UR06-0378/2007(H1N1)), complete sequence

gb|CY025895| Influenza A virus (A/Mississippi/UR06-0537/2007(H1N1)), complete sequence

gb|CY026399| Influenza A virus (A/Mississippi/UR06-0595/2007(H1N1)), complete sequence

gb|CY026383| Influenza A virus (A/New York/UR06-0056/2007(H1N1)), complete sequence

gb|CY026735| Influenza A virus (A/New York/UR06-0134/2007(H1N1)), complete sequence

gb|CY026631| Influenza A virus (A/New York/UR06-0199/2007(H1N1)), complete sequence

gb|CY026647| Influenza A virus (A/New York/UR06-0253/2007(H1N1)), complete sequence

gb|CY027607| Influenza A virus (A/New York/UR06-0326/2007(H1N1)), complete sequence

gb|CY025289| Influenza A virus (A/New York/UR06-0386/2007(H1N1)), complete sequence

gb|CY026863| Influenza A virus (A/North Carolina/UR06-0099/2007(H1N1)), complete sequence

gb|CY026999| Influenza A virus (A/North Carolina/UR06-0364/2007(H1N1)), complete sequence

gb|CY028391| Influenza A virus (A/North Carolina/UR06-0365/2007(H1N1)), complete sequence

gb|CY026959| Influenza A virus (A/Ohio/UR06-0091/2007(H1N1)), complete sequence

gb|CY026007| Influenza A virus (A/Ohio/UR06-0100/2007(H1N1)), complete sequence

gb|CY027767| Influenza A virus (A/Ohio/UR06-0112/2007(H1N1)), complete sequence

gb|CY027447| Influenza A virus (A/Ohio/UR06-0121/2007(H1N1)), complete sequence

gb|CY026015| Influenza A virus (A/Ohio/UR06-0122/2007(H1N1)), complete sequence

gb|CY027407| Influenza A virus (A/Ohio/UR06-0166/2007(H1N1)), complete sequence

gb|CY027143| Influenza A virus (A/Ohio/UR06-0177/2007(H1N1)), complete sequence

gb|CY027391| Influenza A virus (A/Ohio/UR06-0233/2007(H1N1)), complete sequence

gb|CY025975| Influenza A virus (A/Ohio/UR06-0325/2007(H1N1)), complete sequence

gb|CY026855| Influenza A virus (A/Ohio/UR06-0341/2007(H1N1)), complete sequence

gb|CY028127| Influenza A virus (A/Ohio/UR06-0353/2007(H1N1)), complete sequence

gb|CY027471| Influenza A virus (A/Ohio/UR06-0394/2007(H1N1)), complete sequence

gb|CY027991| Influenza A virus (A/Ohio/UR06-0411/2007(H1N1)), complete sequence

gb|CY028407| Influenza A virus (A/Ohio/UR06-0429/2007(H1N1)), complete sequence

gb|CY027071| Influenza A virus (A/Ohio/UR06-0443/2007(H1N1)), complete sequence

gb|CY027007| Influenza A virus (A/Ohio/UR06-0465/2007(H1N1)), complete sequence

gb|CY026951| Influenza A virus (A/Ohio/UR06-0518/2007(H1N1)), complete sequence

gb|CY027263| Influenza A virus (A/Ohio/UR06-0521/2007(H1N1)), complete sequence

gb|CY028031| Influenza A virus (A/Ohio/UR06-0522/2007(H1N1)), complete sequence

gb|CY027775| Influenza A virus (A/Oklahoma/UR06-0063/2007(H1N1)), complete sequence

gb|CY027967| Influenza A virus (A/Oklahoma/UR06-0241/2007(H1N1)), complete sequence

gb|CY028135| Influenza A virus (A/Oklahoma/UR06-0519/2007(H1N1)), complete sequence

gb|CY027695| Influenza A virus (A/Oregon/UR06-0179/2007(H1N1)), complete sequence

gb|CY027959| Influenza A virus (A/Oregon/UR06-0185/2007(H1N1)), complete sequence

gb|CY027927| Influenza A virus (A/Oregon/UR06-0186/2007(H1N1)), complete sequence

gb|CY028215| Influenza A virus (A/Oregon/UR06-0219/2007(H1N1)), complete sequence

gb|CY027087| Influenza A virus (A/Oregon/UR06-0230/2007(H1N1)), complete sequence

gb|CY026903| Influenza A virus (A/Oregon/UR06-0231/2007(H1N1)), complete sequence

gb|CY027935| Influenza A virus (A/Oregon/UR06-0291/2007(H1N1)), complete sequence

gb|CY026703| Influenza A virus (A/Tennessee/UR06-0045/2007(H1N1)), complete sequence

gb|CY028207| Influenza A virus (A/Tennessee/UR06-0055/2007(H1N1)), complete sequence

gb|CY028455| Influenza A virus (A/Tennessee/UR06-0073/2007(H1N1)), complete sequence

gb|CY028111| Influenza A virus (A/Tennessee/UR06-0076/2007(H1N1)), complete sequence

gb|CY026895| Influenza A virus (A/Tennessee/UR06-0078/2007(H1N1)), complete sequence

gb|CY028079| Influenza A virus (A/Tennessee/UR06-0080/2007(H1N1)), complete sequence

gb|CY026511| Influenza A virus (A/Tennessee/UR06-0087/2007(H1N1)), complete sequence

gb|CY027727| Influenza A virus (A/Tennessee/UR06-0113/2007(H1N1)), complete sequence

gb|CY027383| Influenza A virus (A/Tennessee/UR06-0119/2007(H1N1)), complete sequence

gb|CY027135| Influenza A virus (A/Tennessee/UR06-0120/2007(H1N1)), complete sequence

gb|CY027247| Influenza A virus (A/Tennessee/UR06-0124/2007(H1N1)), complete sequence

gb|CY027063| Influenza A virus (A/Tennessee/UR06-0151/2007(H1N1)), complete sequence

gb|CY027119| Influenza A virus (A/Tennessee/UR06-0152/2007(H1N1)), complete sequence

gb|CY027479| Influenza A virus (A/Tennessee/UR06-0234/2007(H1N1)), complete sequence

gb|CY028063| Influenza A virus (A/Tennessee/UR06-0238/2007(H1N1)), complete sequence

gb|CY027271| Influenza A virus (A/Tennessee/UR06-0239/2007(H1N1)), complete sequence

gb|CY027423| Influenza A virus (A/Tennessee/UR06-0262/2007(H1N1)), complete sequence

gb|CY028399| Influenza A virus (A/Tennessee/UR06-0277/2007(H1N1)), complete sequence

gb|CY027255| Influenza A virus (A/Tennessee/UR06-0294/2007(H1N1)), complete sequence

gb|CY028007| Influenza A virus (A/Tennessee/UR06-0312/2007(H1N1)), complete sequence

gb|CY027639| Influenza A virus (A/Tennessee/UR06-0388/2007(H1N1)), complete sequence

gb|CY027455| Influenza A virus (A/Tennessee/UR06-0414/2007(H1N1)), complete sequence

gb|CY027751| Influenza A virus (A/Tennessee/UR06-0459/2007(H1N1)), complete sequence

gb|CY027495| Influenza A virus (A/Tennessee/UR06-0473/2007(H1N1)), complete sequence

gb|CY027735| Influenza A virus (A/Tennessee/UR06-0508/2007(H1N1)), complete sequence

gb|CY028119| Influenza A virus (A/Tennessee/UR06-0509/2007(H1N1)), complete sequence

gb|CY025369| Influenza A virus (A/Texas/UR06-0025/2007(H1N1)), complete sequence

gb|CY028327| Influenza A virus (A/Texas/UR06-0026/2007(H1N1)), complete sequence

gb|CY028295| Influenza A virus (A/Texas/UR06-0038/2007(H1N1)), complete sequence

gb|CY027815| Influenza A virus (A/Texas/UR06-0039/2007(H1N1)), complete sequence

gb|CY027663| Influenza A virus (A/Texas/UR06-0133/2007(H1N1)), complete sequence

gb|CY026967| Influenza A virus (A/Texas/UR06-0157/2007(H1N1)), complete sequence

gb|CY025959| Influenza A virus (A/Texas/UR06-0174/2007(H1N1)), complete sequence

gb|CY026367| Influenza A virus (A/Texas/UR06-0175/2007(H1N1)), complete sequence

gb|CY027183| Influenza A virus (A/Texas/UR06-0176/2007(H1N1)), complete sequence

gb|CY025687| Influenza A virus (A/Texas/UR06-0193/2007(H1N1)), complete sequence

gb|CY026215| Influenza A virus (A/Texas/UR06-0195/2007(H1N1)), complete sequence

gb|CY025529| Influenza A virus (A/Texas/UR06-0196/2007(H1N1)), complete sequence

gb|CY025401| Influenza A virus (A/Texas/UR06-0203/2007(H1N1)), complete sequence

gb|CY027559| Influenza A virus (A/Texas/UR06-0204/2007(H1N1)), complete sequence

gb|CY027623| Influenza A virus (A/Texas/UR06-0216/2007(H1N1)), complete sequence

gb|CY028319| Influenza A virus (A/Texas/UR06-0217/2007(H1N1)), complete sequence

gb|CY028335| Influenza A virus (A/Texas/UR06-0250/2007(H1N1)), complete sequence

gb|CY026407| Influenza A virus (A/Texas/UR06-0270/2007(H1N1)), complete sequence

gb|CY026351| Influenza A virus (A/Texas/UR06-0271/2007(H1N1)), complete sequence

gb|CY026191| Influenza A virus (A/Texas/UR06-0303/2007(H1N1)), complete sequence

gb|CY027167| Influenza A virus (A/Texas/UR06-0305/2007(H1N1)), complete sequence

gb|CY026271| Influenza A virus (A/Texas/UR06-0306/2007(H1N1)), complete sequence

gb|CY026639| Influenza A virus (A/Texas/UR06-0308/2007(H1N1)), complete sequence

gb|CY026599| Influenza A virus (A/Texas/UR06-0309/2007(H1N1)), complete sequence

gb|CY027327| Influenza A virus (A/Texas/UR06-0342/2007(H1N1)), complete sequence

gb|CY027343| Influenza A virus (A/Texas/UR06-0357/2007(H1N1)), complete sequence

gb|CY025695| Influenza A virus (A/Texas/UR06-0359/2007(H1N1)), complete sequence

gb|CY025297| Influenza A virus (A/Texas/UR06-0380/2007(H1N1)), complete sequence

gb|CY026567| Influenza A virus (A/Texas/UR06-0397/2007(H1N1)), complete sequence

gb|CY025393| Influenza A virus (A/Texas/UR06-0398/2007(H1N1)), complete sequence

gb|CY028223| Influenza A virus (A/Texas/UR06-0420/2007(H1N1)), complete sequence

gb|CY026391| Influenza A virus (A/Texas/UR06-0444/2007(H1N1)), complete sequence

gb|CY027599| Influenza A virus (A/Texas/UR06-0445/2007(H1N1)), complete sequence

gb|CY025567| Influenza A virus (A/Texas/UR06-0461/2007(H1N1)), complete sequence

gb|CY025449| Influenza A virus (A/Texas/UR06-0467/2007(H1N1)), complete sequence

gb|CY026503| Influenza A virus (A/Texas/UR06-0468/2007(H1N1)), complete sequence

gb|CY025273| Influenza A virus (A/Texas/UR06-0502/2007(H1N1)), complete sequence

gb|CY027887| Influenza A virus (A/Texas/UR06-0503/2007(H1N1)), complete sequence

gb|CY025591| Influenza A virus (A/Texas/UR06-0526/2007(H1N1)), complete sequence

gb|CY026335| Influenza A virus (A/Texas/UR06-0540/2007(H1N1)), complete sequence

gb|CY026343| Influenza A virus (A/Texas/UR06-0542/2007(H1N1)), complete sequence

gb|CY025551| Influenza A virus (A/Texas/UR06-0563/2007(H1N1)), complete sequence

gb|CY026719| Influenza A virus (A/Texas/UR06-0582/2007(H1N1)), complete sequence

gb|CY026687| Influenza A virus (A/Vermont/UR06-0035/2007(H1N1)), complete sequence

gb|CY025305| Influenza A virus (A/Vermont/UR06-0050/2007(H1N1)), complete sequence

gb|CY025815| Influenza A virus (A/Vermont/UR06-0051/2007(H1N1)), complete sequence

gb|CY025799| Influenza A virus (A/Vermont/UR06-0089/2007(H1N1)), complete sequence

gb|CY025655| Influenza A virus (A/Vermont/UR06-0090/2007(H1N1)), complete sequence

gb|CY025663| Influenza A virus (A/Vermont/UR06-0301/2007(H1N1)), complete sequence

gb|CY027191| Influenza A virus (A/Vermont/UR06-0472/2007(H1N1)), complete sequence

gb|CY025767| Influenza A virus (A/Vermont/UR06-0485/2007(H1N1)), complete sequence

gb|CY026623| Influenza A virus (A/Vermont/UR06-0511/2007(H1N1)), complete sequence

gb|CY026759| Influenza A virus (A/Vermont/UR06-0556/2007(H1N1)), complete sequence

gb|CY026575| Influenza A virus (A/Vermont/UR06-0573/2007(H1N1)), complete sequence

gb|CY025823| Influenza A virus (A/Vermont/UR06-0574/2007(H1N1)), complete sequence

gb|CY026175| Influenza A virus (A/Vermont/UR06-0575/2007(H1N1)), complete sequence

gb|CY025473| Influenza A virus (A/Vermont/UR06-0576/2007(H1N1)), complete sequence

gb|CY027831| Influenza A virus (A/Virginia/UR06-0075/2007(H1N1)), complete sequence

gb|CY025999| Influenza A virus (A/Virginia/UR06-0109/2007(H1N1)), complete sequence

gb|CY027759| Influenza A virus (A/Virginia/UR06-0114/2007(H1N1)), complete sequence

gb|CY027239| Influenza A virus (A/Virginia/UR06-0117/2007(H1N1)), complete sequence

gb|CY026839| Influenza A virus (A/Virginia/UR06-0139/2007(H1N1)), complete sequence

gb|CY026943| Influenza A virus (A/Virginia/UR06-0164/2007(H1N1)), complete sequence

gb|CY027031| Influenza A virus (A/Virginia/UR06-0244/2007(H1N1)), complete sequence

gb|CY025991| Influenza A virus (A/Virginia/UR06-0245/2007(H1N1)), complete sequence

gb|CY026911| Influenza A virus (A/Virginia/UR06-0254/2007(H1N1)), complete sequence

gb|CY027975| Influenza A virus (A/Virginia/UR06-0266/2007(H1N1)), complete sequence

gb|CY026935| Influenza A virus (A/Virginia/UR06-0267/2007(H1N1)), complete sequence

gb|CY027943| Influenza A virus (A/Virginia/UR06-0295/2007(H1N1)), complete sequence

gb|CY027647| Influenza A virus (A/Virginia/UR06-0297/2007(H1N1)), complete sequence

gb|CY028383| Influenza A virus (A/Virginia/UR06-0332/2007(H1N1)), complete sequence

gb|CY028415| Influenza A virus (A/Virginia/UR06-0346/2007(H1N1)), complete sequence

gb|CY028159| Influenza A virus (A/Virginia/UR06-0351/2007(H1N1)), complete sequence

gb|CY027863| Influenza A virus (A/Virginia/UR06-0360/2007(H1N1)), complete sequence

gb|CY026919| Influenza A virus (A/Virginia/UR06-0384/2007(H1N1)), complete sequence

gb|CY028103| Influenza A virus (A/Virginia/UR06-0387/2007(H1N1)), complete sequence

gb|CY027711| Influenza A virus (A/Virginia/UR06-0549/2007(H1N1)), complete sequence

gb|CY027655| Influenza A virus (A/Virginia/UR06-0562/2007(H1N1)), complete sequence

gb|CY027951| Influenza A virus (A/Virginia/UR06-0594/2007(H1N1)), complete sequence

gb|CY021041| Influenza A virus (A/Christ's Hospital/157/1982(H1N1)), complete sequence
