# Supplementary material for: Patterns of Oligonucleotide Sequences in Viral and Host Cell RNA Identify Mediators of the Host Innate Immune System
Source: PLoS One. 2009 Jun 18;4(6):e5969. doi: 10.1371/journal.pone.0005969 (PMC2694999; doi:10.1371/journal.pone.0005969)
Supplement: Table S2 — The ssRNA viruses whose CDS regions were used in this analysis. (0.05 MB DOC) [file pone.0005969.s002.doc]

ref|NC_001943|Human astrovirus

ref|NC_002640|Dengue virus 4

ref|NC_001477|Dengue virus 1

ref|NC_001475|Dengue virus 3

ref|NC_001474|Dengue virus 2

ref|NC_002058|Poliovirus

ref|NC_009827|Hepatitis C virus genotype 6

ref|NC_009826|Hepatitis C virus genotype 5

ref|NC_009824|Hepatitis C virus genotype 3

ref|NC_009823|Hepatitis C virus genotype 2

ref|NC_004102|Hepatitis C virus

ref|NC_001959|Norwalk virus

ref|NC_010414|Simian picornavirus 13

ref|NC_001612|Human enterovirus A

ref|NC_001472|Human enterovirus B

ref|NC_001428|Human enterovirus C

ref|NC_003988|Simian enterovirus A

ref|NC_010413|Simian enterovirus SV43

ref|NC_010412|Simian enterovirus SV19

ref|NC_009887|Human enterovirus 100

ref|NC_001489|Hepatitis A

ref|NC_009996|Human rhinovirus C

ref|NC_001617|Human rhinovirus 89

ref|NC_001490|Human rhinovirus B

ref|NC_002645|Human coronavirus 229E

ref|NC_005831|Human coronavirus NL63

ref|NC_006577|Human coronavirus HKU1

ref|NC_005147|Human coronavirus OC43

ref|NC_001897|Human parechovirus

ref|NC_010411|Simian picornavirus strain 17

ref|NC_010384|Simian picornavirus strain N125

ref|NC_004451|Simian picornavirus 1

ref|NC_001545|Rubella virus

ref|NC_004294|Lymphocytic choriomeningitis virus

ref|NC_002020|Influenza A virus (A/Puerto Rico/8/34(H1N1))

ref|NC_007373|Influenza A virus (A/New York/392/2004(H3N2))

ref|NC_002211|Influenza B virus

ref|NC_006306|Influenza C virus

ref|NC_004148|Human metapneumovirus

ref|NC_001498|Measles virus

ref|NC_003461|Human parainfluenza virus 1 strain Washington/1964

ref|NC_001796|Human parainfluenza virus 3

ref|NC_003443|Human parainfluenza virus 2

ref|NC_006430|Simian parainfluenza virus 5

ref|NC_001781|Human respiratory syncytial virus

ref|NC_006428|Simian Influenza Virus 41

ref|NC_002200|Mumps virus
