# Supplementary material for: Patterns of Oligonucleotide Sequences in Viral and Host Cell RNA Identify Mediators of the Host Innate Immune System
Source: PLoS One. 2009 Jun 18;4(6):e5969. doi: 10.1371/journal.pone.0005969 (PMC2694999; doi:10.1371/journal.pone.0005969)
Supplement: Table S3 — The 56 under-represented motifs of length four, listed in order of ascending p-values, that are significant in all groups of expressed genes at the 2-, 4- and 8-fold level and are taken from the CDS regions of these genes. (0.08 MB DOC) [file pone.0005969.s003.doc]

| CGAA | 0.4306 |
| --- | --- |
| TCGA | 0.4498 |
| CGAT | 0.4671 |
| GTCG | 0.4695 |
| CGTA | 0.4699 |
| GCGA | 0.4813 |
| TACG | 0.5111 |
| CGAC | 0.5147 |
| ACGA | 0.5242 |
| CCGA | 0.544 |
| AACG | 0.5502 |
| GACG | 0.5644 |
| CGAG | 0.5661 |
| TCGT | 0.5689 |
| ATCG | 0.5721 |
| CGCG | 0.5967 |
| TTCG | 0.5976 |
| CGTT | 0.6003 |
| TCGC | 0.6026 |
| GCCG | 0.6168 |
| TAAG | 0.6183 |
| ACCG | 0.6263 |
| TAGT | 0.6277 |
| CGCA | 0.6281 |
| CTCG | 0.6307 |
| GCGT | 0.6331 |
| TTAG | 0.6364 |
| GGCG | 0.6459 |
| TAGC | 0.6489 |
| ATTA | 0.6495 |
| AGCG | 0.6496 |
| ACGT | 0.6525 |
| ACGC | 0.6538 |
| CGTC | 0.6556 |
| CGCC | 0.6575 |
| CCGT | 0.666 |
| GTAA | 0.6693 |
| TATA | 0.6766 |
| CGTG | 0.6799 |
| CTAA | 0.6835 |
| GATA | 0.6857 |
| TAGG | 0.6922 |
| TATC | 0.6943 |
| CCGC | 0.6944 |
| CCCG | 0.6973 |
| TCCG | 0.6974 |
| TAAC | 0.702 |
| TGCG | 0.7048 |
| ACGG | 0.7124 |
| TAAT | 0.7147 |
| ATAC | 0.7182 |
| CGGT | 0.7185 |
| TTTA | 0.7193 |
| CGGC | 0.72 |
| TTAT | 0.7305 |
| CCGG | 0.7545 |
