# Supplementary material for: Patterns of Oligonucleotide Sequences in Viral and Host Cell RNA Identify Mediators of the Host Innate Immune System
Source: PLoS One. 2009 Jun 18;4(6):e5969. doi: 10.1371/journal.pone.0005969 (PMC2694999; doi:10.1371/journal.pone.0005969)
Supplement: Table S4 — The 13 over-represented motifs in expressed genes by the same criteria as Table S3, ranked in descending order. (0.04 MB DOC) [file pone.0005969.s004.doc]

| TTTG | 1.3476 |
| --- | --- |
| ATGA | 1.3456 |
| TGTG | 1.3442 |
| GATG | 1.3165 |
| CCAT | 1.3079 |
| GTGG | 1.2937 |
| ACCA | 1.2706 |
| CTGG | 1.2679 |
| CATC | 1.2651 |
| GCTG | 1.2622 |
| CAGC | 1.2513 |
| CAGA | 1.2507 |
| TGGA | 1.2418 |
