# Supplementary material for: Patterns of Oligonucleotide Sequences in Viral and Host Cell RNA Identify Mediators of the Host Innate Immune System
Source: PLoS One. 2009 Jun 18;4(6):e5969. doi: 10.1371/journal.pone.0005969 (PMC2694999; doi:10.1371/journal.pone.0005969)
Supplement: Table S5 — The genes whose CpG frequency is in the lowest 10% of the mouse genome, with the gene name and Entrez ID gene numbers both listed. B) The lowest genes bythe same criterion in the human genome. (0.06 MB DOC) [file pone.0005969.s005.doc]

| CGAT | 0.5402 |
| --- | --- |
| CGAA | 0.5422 |
| TTCG | 0.5478 |
| ACGA | 0.5479 |
| TCGA | 0.5488 |
| GACG | 0.5639 |
| TACG | 0.5834 |
| TCGT | 0.5958 |
| GTCG | 0.6004 |
| ATCG | 0.6066 |
| CGTT | 0.6145 |
| AACG | 0.615 |
| TCCG | 0.6353 |
| CGCT | 0.6374 |
| TCGC | 0.646 |
| GCGA | 0.649 |
| CGTA | 0.6541 |
| CGGA | 0.6571 |
| CGAC | 0.6617 |
| ACCG | 0.6654 |
| CGGT | 0.6731 |
| CGAG | 0.6732 |
| CCGA | 0.6756 |
| CGCA | 0.678 |
| ACGG | 0.6855 |
| ACGC | 0.686 |
| TCGG | 0.7056 |
| CCGT | 0.7061 |
| CTCG | 0.7188 |
| CCGG | 0.7214 |
| CGTC | 0.7234 |
| GGCG | 0.7295 |
| ACGT | 0.73 |
| CCGC | 0.749 |
| AGCG | 0.7573 |
| GCCG | 0.7592 |
