# Supplementary material for: Patterns of Oligonucleotide Sequences in Viral and Host Cell RNA Identify Mediators of the Host Innate Immune System
Source: PLoS One. 2009 Jun 18;4(6):e5969. doi: 10.1371/journal.pone.0005969 (PMC2694999; doi:10.1371/journal.pone.0005969)
Supplement: Table S6 — The lowest genes by the same criterion as Table S5, but in the human genome. (0.03 MB DOC) [file pone.0005969.s006.doc]

| TGTG | 1.2408 |
| --- | --- |
| CCAC | 1.2741 |
| CACC | 1.2378 |
| CACA | 1.2482 |
| ACCA | 1.2905 |
| ACAC | 1.2615 |
