# Supplementary material for: Patterns of Oligonucleotide Sequences in Viral and Host Cell RNA Identify Mediators of the Host Innate Immune System
Source: PLoS One. 2009 Jun 18;4(6):e5969. doi: 10.1371/journal.pone.0005969 (PMC2694999; doi:10.1371/journal.pone.0005969)
Supplement: Table S7 — The 33 shared under-represented motifs for the genes and viruses, ranked by the viral p-value in ascending order. (0.05 MB DOC) [file pone.0005969.s007.doc]

| CGAT |
| --- |
| CGAA |
| TTCG |
| ACGA |
| TCGA |
| GACG |
| TACG |
| TCGT |
| GTCG |
| ATCG |
| CGTT |
| AACG |
| TCCG |
| TCGC |
| GCGA |
| CGTA |
| CGAC |
| ACCG |
| CGGT |
| CGAG |
| CCGA |
| CGCA |
| ACGG |
| ACGC |
| CCGT |
| CTCG |
| CCGG |
| CGTC |
| GGCG |
| ACGT |
| CCGC |
| AGCG |
| GCCG |
