# Supplementary material for: Patterns of Oligonucleotide Sequences in Viral and Host Cell RNA Identify Mediators of the Host Innate Immune System
Source: PLoS One. 2009 Jun 18;4(6):e5969. doi: 10.1371/journal.pone.0005969 (PMC2694999; doi:10.1371/journal.pone.0005969)
Supplement: Table S8 — The two shared over-represented motifs for genes and viruses, in descending order. (0.03 MB DOC) [file pone.0005969.s008.doc]

| ACCA |
| --- |
| TGTG |
