# Supplementary material for: Patterns of Oligonucleotide Sequences in Viral and Host Cell RNA Identify Mediators of the Host Innate Immune System
Source: PLoS One. 2009 Jun 18;4(6):e5969. doi: 10.1371/journal.pone.0005969 (PMC2694999; doi:10.1371/journal.pone.0005969)
Supplement: Table S9 — The genes whose CpG frequency is in the lowest 10% of the mouse genome, with the gene name and Entrez ID gene numbers both listed. (1.22 MB DOC) [file pone.0005969.s009.doc]

| Mouse Gene Name | Mouse Entrez ID |
| --- | --- |
| 0610010F05RIK | 71675 |
| 0610037D15RIK | 68394 |
| 1100001G20RIK | 66107 |
| 1110006O17RIK | 68545 |
| 1110012L19RIK | 68618 |
| 1110028C15RIK | 68691 |
| 1200016B10RIK | 66875 |
| 1700008G05RIK | 75497 |
| 1700008I05RIK | 71841 |
| 1700012A03RIK | 76382 |
| 1700016D06RIK | 76413 |
| 1700016G05RIK | 67690 |
| 1700019G17RIK | 75541 |
| 1700022C21RIK | 76416 |
| 1700025D03RIK | 67944 |
| 1700034K16RIK | 73314 |
| 1700034O15RIK | 76606 |
| 1700049G17RIK | 73430 |
| 1700049K14RIK | 73382 |
| 1700065O13RIK | 73451 |
| 1700074P13RIK | 73481 |
| 1700109H08RIK | 77036 |
| 1700129I04RIK | 330577 |
| 1810008K16RIK | 67366 |
| 1810009J06RIK | 73626 |
| 1810023F06RIK | 217845 |
| 1810030J14RIK | 66289 |
| 1810049H19RIK | 435889 |
| 2010002M12RIK | 112419 |
| 2010003J03RIK | 69860 |
| 2010005H15RIK | 76770 |
| 2010106E10RIK | 67715 |
| 2010107E04RIK | 70257 |
| 2010311D03RIK | 109129 |
| 2210010B09RIK | 244721 |
| 2210010C04RIK | 67373 |
| 2210407C18RIK | 78354 |
| 2310008H04RIK | 224008 |
| 2310038E17RIK | 71903 |
| 2310051M13RIK | 70202 |
| 2310057J18RIK | 67719 |
| 2310057N15RIK | 69696 |
| 2410004F06RIK | 71981 |
| 2410042D21RIK | 72425 |
| 2610008E11RIK | 72128 |
| 2610020H08RIK | 434234 |
| 2610034M16RIK | 69239 |
| 2610207I05RIK | 233789 |
| 2700019D07RIK | 72580 |
| 2810002O09RIK | 72345 |
| 2810007J24RIK | 76971 |
| 2810426N06RIK | 67607 |
| 2810474O19RIK | 67246 |
| 2810485I05RIK | 72826 |
| 2810487A22RIK | 72807 |
| 2900006K08RIK | 72873 |
| 2900024C23RIK | 67266 |
| 2900042B11RIK | 72938 |
| 2900092C05RIK | 73090 |
| 3110001A13RIK | 66540 |
| 3110001K24RIK | 75698 |
| 3110031B13RIK | 67288 |
| 3632451O06RIK | 67419 |
| 3830403N18RIK | 70691 |
| 3830406C13RIK | 218734 |
| 3830417A13RIK | 70696 |
| 4632404H22RIK | 78755 |
| 4632417K18RIK | 107373 |
| 4632434I11RIK | 74041 |
| 4732418C07RIK | 230648 |
| 4732429D16RIK | 217305 |
| 4732471D19RIK | 319719 |
| 4732496O08RIK | 242736 |
| 4733401H21RIK | 70831 |
| 4832428D23RIK | 403183 |
| 4833413O15RIK | 242286 |
| 4833442J19RIK | 320204 |
| 4921501E09RIK | 74042 |
| 4921504E06RIK | 70909 |
| 4921513D23RIK | 223989 |
| 4930418G15RIK | 69312 |
| 4930425N13RIK | 73893 |
| 4930430A15RIK | 67575 |
| 4930486L24RIK | 214639 |
| 4930503E14RIK | 74954 |
| 4930521A18RIK | 74708 |
| 4930522H14RIK | 67646 |
| 4930523C11RIK | 238405 |
| 4930542N07RIK | 75185 |
| 4930547C10RIK | 68274 |
| 4930548H24RIK | 67656 |
| 4930550C14RIK | 75311 |
| 4930550L24RIK | 75352 |
| 4930563P21RIK | 75275 |
| 4930579C15RIK | 67753 |
| 4931406B18RIK | 74054 |
| 4931406C07RIK | 70984 |
| 4931407G18RIK | 70977 |
| 4932438H23RIK | 74387 |
| 4933421E11RIK | 321000 |
| 4933436I01RIK | 66780 |
| 5033413D22RIK | 75973 |
| 5430402E10RIK | 71351 |
| 5730403B10RIK | 66626 |
| 5730494M16RIK | 66648 |
| 5730577I03RIK | 66662 |
| 5730596K20RIK | 109168 |
| 5830417C01RIK | 78825 |
| 5830443L24RIK | 76074 |
| 5830483C08RIK | 209334 |
| 6030408C04RIK | 217558 |
| 6030498E09RIK | 77883 |
| 6230416J20RIK | 230376 |
| 8030411F24RIK | 78609 |
| 8430426H19RIK | 71508 |
| 39880 | 71779 |
| 9230104L09RIK | 77705 |
| 9330134C04RIK | 432766 |
| 9430020K01RIK | 240185 |
| 9530002B09RIK | 77432 |
| 9930021J03RIK | 240613 |
| A030001H23RIK | 230613 |
| A030004J04RIK | 109314 |
| A130090K04RIK | 320495 |
| A530054K11RIK | 212281 |
| A530088H08RIK | 193003 |
| A630095E13RIK | 235973 |
| A830080D01RIK | 382252 |
| A930001N09RIK | 77128 |
| A930038C07RIK | 68169 |
| AADAT | 23923 |
| ABCA13 | 268379 |
| ABCA14 | 67928 |
| ABCG3 | 27405 |
| ABPA | 11354 |
| ABPD | 494519 |
| ACE2 | 70008 |
| ACOT10 | 64833 |
| ACSM3 | 20216 |
| ACTRT1 | 73360 |
| ACVR2A | 11480 |
| ADAL | 75894 |
| ADAM21 | 56622 |
| ADAM24 | 13526 |
| ADAM26A | 13525 |
| ADAM28 | 13522 |
| ADAM34 | 252866 |
| ADAM4 | 11498 |
| ADAM6 | 238406 |
| ADAM7 | 11500 |
| ADAMTS6 | 108154 |
| AFF2 | 14266 |
| AFTPH | 216549 |
| AGR3 | 403205 |
| AGTR2 | 11609 |
| AHNAK | 66395 |
| AHRR | 11624 |
| AI314180 | 230249 |
| AI449175 | 234362 |
| AI451617 | 209387 |
| AI597468 | 103266 |
| AI747448 | 99709 |
| AI747699 | 381236 |
| AI842396 | 103844 |
| AI987662 | 101202 |
| AI987944 | 233168 |
| AIM2 | 383619 |
| AK129302 | 245522 |
| AKAP4 | 11643 |
| AKR1B7 | 11997 |
| AKR1C12 | 622402 |
| AKR1C13 | 27384 |
| AKR1C18 | 105349 |
| AKR1C19 | 432720 |
| AKR1C21 | 77337 |
| AKR1E1 | 56043 |
| ALDH1A7 | 26358 |
| ALKBH8 | 67667 |
| ALMS1 | 236266 |
| AMELX | 11704 |
| AMY1 | 11722 |
| ANAPC10 | 68999 |
| ANGPTL3 | 30924 |
| ANKRD12 | 106585 |
| ANKRD32 | 105377 |
| ANKRD7 | 75196 |
| ANKS4B | 72074 |
| AP3S1 | 11777 |
| APCS | 20219 |
| APOBEC4 | 71281 |
| APOC4 | 11425 |
| APOL7A | 75761 |
| APOL7B | 278679 |
| APOL7C | 108956 |
| APOOL | 68117 |
| ARFGEF1 | 211673 |
| ARHGAP5 | 11855 |
| ARID4B | 94246 |
| ARL1 | 104303 |
| ARL6 | 56297 |
| ARL6IP1 | 54208 |
| ARMCX1 | 78248 |
| ARMCX6 | 278097 |
| ART2B | 11872 |
| ARTS1 | 80898 |
| ASB3 | 65257 |
| ASNSD1 | 70396 |
| ASPA | 11484 |
| ASPN | 66695 |
| ASXL2 | 75302 |
| ATAD5 | 237877 |
| ATF2 | 11909 |
| ATG3 | 67841 |
| ATP11B | 76295 |
| ATP11C | 320940 |
| ATP5H | 71679 |
| ATP5J | 11957 |
| ATRX | 22589 |
| AV249152 | 216560 |
| AW061290 | 381110 |
| AW551984 | 244810 |
| AY761184 | 382000 |
| AZGP1 | 12007 |
| AZI2 | 27215 |
| AZIN1 | 54375 |
| B020006M18RIK | 546118 |
| B230218L05RIK | 330998 |
| B3GNT5 | 108105 |
| BAAT | 12012 |
| BANK1 | 242248 |
| BBS12 | 241950 |
| BBS4 | 102774 |
| BC003331 | 226499 |
| BC011426 | 224893 |
| BC011487 | 232748 |
| BC014805 | 236149 |
| BC016495 | 225994 |
| BC023829 | 236848 |
| BC023882 | 231123 |
| BC026439 | 210463 |
| BC026782 | 545366 |
| BC031441 | 240120 |
| BC037393 | 381695 |
| BC049807 | 381066 |
| BC050092 | 235048 |
| BC050789 | 213234 |
| BCL2A1A | 12044 |
| BET1 | 12068 |
| BEX6 | 328660 |
| BHLHB9 | 70237 |
| BIRC1F | 17952 |
| BIVM | 246229 |
| BMI1 | 12151 |
| BMX | 12169 |
| BRCA2 | 12190 |
| BRCC3 | 210766 |
| BRE | 107976 |
| BTLA | 208154 |
| BZRPL1 | 70026 |
| C030039L03RIK | 112415 |
| C130079G13RIK | 229333 |
| C1D | 57316 |
| C1GALT1 | 94192 |
| C4BP | 12269 |
| C6 | 12274 |
| C730048C13RIK | 319800 |
| C79407 | 217653 |
| CALCA | 12310 |
| CANX | 12330 |
| CAPRIN2 | 232560 |
| CAR1 | 12346 |
| CARF | 241066 |
| CASC5 | 76464 |
| CASP1 | 12362 |
| CASP12 | 12364 |
| CASP4 | 12363 |
| CASP8AP2 | 26885 |
| CAST | 12380 |
| CBWD1 | 226043 |
| CBX1 | 12412 |
| CCDC122 | 108811 |
| CCDC18 | 73254 |
| CCDC54 | 69339 |
| CCDC7 | 74703 |
| CCDC73 | 211936 |
| CCDC79 | 320022 |
| CCDC88A | 108686 |
| CCL1 | 20290 |
| CCL11 | 20292 |
| CCL2 | 20296 |
| CCL4 | 20303 |
| CCL8 | 20307 |
| CCL9 | 20308 |
| CCNB3 | 209091 |
| CCNC | 51813 |
| CCNE2 | 12448 |
| CCNI | 12453 |
| CCR1 | 12768 |
| CCR2 | 12772 |
| CCR3 | 12771 |
| CCR5 | 12774 |
| CCR9 | 12769 |
| CD160 | 54215 |
| CD180 | 17079 |
| CD200R1 | 57781 |
| CD200R4 | 239849 |
| CD209A | 170786 |
| CD209C | 170776 |
| CD209D | 170779 |
| CD209E | 170780 |
| CD226 | 225825 |
| CD244 | 18106 |
| CD300A | 217303 |
| CD300D | 140497 |
| CD33 | 12489 |
| CD36 | 12491 |
| CD46 | 17221 |
| CD53 | 12508 |
| CD59A | 12509 |
| CD84 | 12523 |
| CD96 | 84544 |
| CDC27 | 217232 |
| CDC42 | 12540 |
| CDH19 | 227485 |
| CEACAM11 | 66996 |
| CEACAM12 | 67315 |
| CEACAM13 | 69785 |
| CEACAM14 | 67084 |
| CENPF | 108000 |
| CENPI | 102920 |
| CENPK | 60411 |
| CENPL | 70454 |
| CENPQ | 83815 |
| CEP27 | 66296 |
| CEP68 | 216543 |
| CEP70 | 68121 |
| CEPT1 | 99712 |
| CES1 | 12623 |
| CES2 | 234671 |
| CES3 | 104158 |
| CES5 | 234673 |
| CES6 | 102022 |
| CETN2 | 26370 |
| CFHR1 | 50702 |
| CFLAR | 12633 |
| CGA | 12640 |
| CHI3L3 | 12655 |
| CHI3L4 | 104183 |
| CHIA | 81600 |
| CHML | 12663 |
| CHORDC1 | 66917 |
| CHST9 | 71367 |
| CKAP5 | 75786 |
| CLCA6 | 99663 |
| CLEC1A | 243653 |
| CLEC2E | 232409 |
| CLEC2H | 94071 |
| CLEC4A2 | 26888 |
| CLEC4A3 | 73149 |
| CLEC4B1 | 69810 |
| CLEC4E | 56619 |
| CLEC4N | 56620 |
| CLOCK | 12753 |
| CMA1 | 17228 |
| CMA2 | 545055 |
| CML2 | 93673 |
| CML5 | 69049 |
| CNGB3 | 30952 |
| CNKSR2 | 245684 |
| CNOT6 | 104625 |
| CNOT6L | 231464 |
| CNTN6 | 53870 |
| COL4A5 | 12830 |
| COL4A6 | 94216 |
| COL5A2 | 12832 |
| COLEC10 | 239447 |
| COLEC12 | 140792 |
| COPS2 | 12848 |
| COPS7B | 26895 |
| COX7B2 | 78174 |
| CP | 12870 |
| CPNE3 | 70568 |
| CR2 | 12902 |
| CRB1 | 170788 |
| CREB1 | 12912 |
| CRISP1 | 11571 |
| CRISP2 | 22024 |
| CRISP3 | 11572 |
| CRISP4 | 78081 |
| CRISPLD1 | 83691 |
| CRRY | 12946 |
| CSE1L | 110750 |
| CSF3R | 12986 |
| CSN1S2A | 12993 |
| CSN1S2B | 12992 |
| CSN2 | 12991 |
| CSN3 | 12994 |
| CST10 | 58214 |
| CST13 | 69294 |
| CST7 | 13011 |
| CST8 | 13012 |
| CST9 | 13013 |
| CSTAD | 78617 |
| CTS3 | 117066 |
| CTS6 | 58518 |
| CTS7 | 56092 |
| CTS8 | 56094 |
| CTSM | 64139 |
| CTSQ | 104002 |
| CTSR | 56835 |
| CUGBP1 | 13046 |
| CWF19L2 | 244672 |
| CXCL15 | 20309 |
| CXCR6 | 80901 |
| CYB5D2 | 192986 |
| CYCS | 13063 |
| CYLC1 | 67407 |
| CYLD | 74256 |
| CYP2C29 | 13095 |
| CYP2C37 | 13096 |
| CYP2C38 | 13097 |
| CYP2C39 | 13098 |
| CYP2C50 | 107141 |
| CYP2C54 | 404195 |
| CYP2C55 | 72082 |
| CYP2C65 | 72303 |
| CYP2C68 | 433247 |
| CYP2C70 | 226105 |
| CYP2J11 | 100066 |
| CYP2J5 | 13109 |
| CYP2J6 | 13110 |
| CYP39A1 | 56050 |
| CYP3A11 | 13112 |
| CYP3A13 | 13113 |
| CYP3A16 | 13114 |
| CYP3A25 | 56388 |
| CYP3A41A | 53973 |
| CYP3A44 | 337924 |
| CYP4A10 | 13117 |
| CYP4A12A | 277753 |
| CYP4A14 | 13119 |
| CYSLTR1 | 58861 |
| CYSLTR2 | 70086 |
| D12ERTD647E | 52668 |
| D14ERTD668E | 219132 |
| D19ERTD386E | 52013 |
| D19ERTD737E | 76539 |
| D3ERTD751E | 73852 |
| D630002G06RIK | 236293 |
| D730048I06RIK | 68171 |
| DARC | 13349 |
| DAZAP2 | 23994 |
| DAZL | 13164 |
| DBIL5 | 13168 |
| DBPHT2 | 386753 |
| DCUN1D1 | 114893 |
| DEFB1 | 13214 |
| DEFB10 | 246085 |
| DEFB13 | 246083 |
| DEFB15 | 246082 |
| DEFB2 | 13215 |
| DEFB21 | 403172 |
| DEFB3 | 27358 |
| DEFB5 | 81007 |
| DEFB9 | 246079 |
| DEFCR23 | 497114 |
| DEFCR24 | 503491 |
| DEFCR5 | 13239 |
| DEPDC1A | 76131 |
| DMD | 13405 |
| DMXL1 | 240283 |
| DNASE1L1 | 69537 |
| DOCK11 | 75974 |
| DPH4 | 99349 |
| DPP10 | 269109 |
| DSC1 | 13505 |
| DSC3 | 13507 |
| DSEL | 319901 |
| DUB1 | 13531 |
| DUB2 | 13532 |
| DUB2A | 384701 |
| DZIP3 | 224170 |
| E230025N22RIK | 240216 |
| E330009P21RIK | 320082 |
| E430004N04RIK | 210757 |
| EAF2 | 106389 |
| EAR1 | 13586 |
| EAR2 | 13587 |
| ECM2 | 407800 |
| EDA2R | 245527 |
| EFCAB1 | 66793 |
| EG214321 | 214321 |
| EG214403 | 214403 |
| EG240327 | 240327 |
| EG380907 | 380907 |
| EG381936 | 381936 |
| EG382156 | 382156 |
| EG434179 | 434179 |
| EG434674 | 434674 |
| EG436523 | 436523 |
| EG547109 | 547109 |
| EG622139 | 622139 |
| EG622976 | 622976 |
| EG624866 | 624866 |
| EG627927 | 627927 |
| EG630579 | 630579 |
| EG636104 | 636104 |
| EIF1A | 13664 |
| EIF2A | 229317 |
| ELF4 | 56501 |
| ENAM | 13801 |
| ENSMUSG00000053178 | 208595 |
| EPS15 | 13858 |
| ERBB2IP | 59079 |
| ERCC6L | 236930 |
| ERGIC2 | 67456 |
| ES1 | 13884 |
| ES22 | 13897 |
| ESCO1 | 77805 |
| ESCO2 | 71988 |
| ESF1 | 66580 |
| EVI2B | 216984 |
| EXOD1 | 71151 |
| F730047E07RIK | 212377 |
| F8 | 14069 |
| F9 | 14071 |
| FAHD2A | 68126 |
| FANCL | 67030 |
| FAP | 14089 |
| FASL | 14103 |
| FASTKD2 | 75619 |
| FBXO39 | 327959 |
| FBXO43 | 78803 |
| FBXO8 | 50753 |
| FBXW16 | 320083 |
| FBXW19 | 235612 |
| FBXW2 | 30050 |
| FCER1A | 14125 |
| FCGR4 | 246256 |
| FCNB | 14134 |
| FGD4 | 224014 |
| FGF14 | 14169 |
| FHL4 | 14202 |
| FIGNL1 | 60530 |
| FLYWCH2 | 76917 |
| FNBP1L | 214459 |
| FNIP1 | 216742 |
| FOLH1 | 53320 |
| FOXN2 | 14236 |
| FPGT | 75540 |
| FPR1 | 14293 |
| FPRL1 | 14294 |
| FPR-RS2 | 14289 |
| FRG1 | 14300 |
| FRMD3 | 242506 |
| FSTL5 | 213262 |
| FUNDC2 | 67391 |
| FUT9 | 14348 |
| FXYD4 | 108017 |
| GAB3 | 210710 |
| GABRA3 | 14396 |
| GABRA6 | 14399 |
| GABRG2 | 14406 |
| GALNT5 | 241391 |
| GBP1 | 14468 |
| GBP2 | 14469 |
| GC | 14473 |
| GCC2 | 70297 |
| GEMIN4 | 276919 |
| GGPS1 | 14593 |
| GHITM | 66092 |
| GHR | 14600 |
| GHRL | 58991 |
| GIMAP4 | 107526 |
| GIMAP5 | 317757 |
| GIMAP8 | 243374 |
| GJE1 | 118446 |
| GLCE | 93683 |
| GLMN | 170823 |
| GLRA2 | 237213 |
| GLRP1 | 14659 |
| GLT6D1 | 71103 |
| GLT8D1 | 76485 |
| GLYAT | 107146 |
| GLYCAM1 | 14663 |
| GM732 | 213450 |
| GM784 | 333564 |
| GM97 | 225923 |
| GMCL1L | 71847 |
| GMPS | 229363 |
| GNG13 | 64337 |
| GNGT1 | 14699 |
| GNGT2 | 14710 |
| GNL3 | 30877 |
| GOLGA1 | 76899 |
| GP1BA | 14723 |
| GP2 | 67133 |
| GP49A | 14727 |
| GPR1 | 241070 |
| GPR171 | 229323 |
| GPR174 | 213439 |
| GPR177 | 68151 |
| GPR23 | 78134 |
| GPR33 | 14762 |
| GPR34 | 23890 |
| GPR75 | 237716 |
| GPRASP1 | 67298 |
| GPRC2A-RS5 | 75835 |
| GRK4 | 14772 |
| GSBS | 19051 |
| GSDMC1 | 83492 |
| GSTA1 | 14857 |
| GSTA2 | 14858 |
| GT4-1 | 107459 |
| GTF2H2 | 23894 |
| GVIN1 | 74558 |
| GZME | 14942 |
| GZMF | 14943 |
| GZMN | 245839 |
| H2-M10.1 | 14985 |
| H2-M9 | 14997 |
| H2-OB | 15002 |
| HADHB | 231086 |
| HAMP | 84506 |
| HAMP2 | 66438 |
| HAO3 | 56185 |
| HAVCR2 | 171285 |
| HBB-B1 | 15129 |
| HBB-BH1 | 15132 |
| HBB-Y | 15135 |
| HBP1 | 73389 |
| HEMGN | 93966 |
| HEMT1 | 15202 |
| HEPH | 15203 |
| HERC4 | 67345 |
| HMGB1 | 15289 |
| HMGB3 | 15354 |
| HMMR | 15366 |
| HMOX2 | 15369 |
| HNMT | 140483 |
| HOMER1 | 26556 |
| HORMAD1 | 67981 |
| HSD3B1 | 15492 |
| HSD3B2 | 15493 |
| HSD3B3 | 15494 |
| HSD3B4 | 15495 |
| HSD3B5 | 15496 |
| HSD3B6 | 15497 |
| HSF2 | 15500 |
| HTATSF1 | 72459 |
| HYAL5 | 74468 |
| IDI2 | 320581 |
| IFI202B | 26388 |
| IFI203 | 15950 |
| IFI204 | 15951 |
| IFI205 | 226695 |
| IFI44 | 99899 |
| IFIT1 | 15957 |
| IFIT2 | 15958 |
| IFNA1 | 15962 |
| IFNA11 | 15964 |
| IFNA12 | 242519 |
| IFNA14 | 404549 |
| IFNA2 | 15965 |
| IFNA4 | 15967 |
| IFNA5 | 15968 |
| IFNA6 | 15969 |
| IFNA7 | 15970 |
| IFNA9 | 15972 |
| IFNAB | 15974 |
| IFRG15 | 64164 |
| IGSF1 | 209268 |
| IIGP1 | 60440 |
| IL13RA2 | 16165 |
| IL15 | 16168 |
| IL15RA | 16169 |
| IL18 | 16173 |
| IL18BP | 16068 |
| IL18RAP | 16174 |
| IL1B | 16176 |
| IL1F6 | 54448 |
| IL1F8 | 69677 |
| IL1RAPL2 | 60367 |
| IL1RN | 16181 |
| IL2 | 16183 |
| IL20 | 58181 |
| IL21 | 60505 |
| IL24 | 93672 |
| IL5RA | 16192 |
| IL6 | 16193 |
| ILF2 | 67781 |
| INSL5 | 23919 |
| INTS12 | 71793 |
| INTS6 | 18130 |
| IPO7 | 233726 |
| IQCB1 | 320299 |
| IREB2 | 64602 |
| ISX | 71597 |
| ITGB1BP2 | 26549 |
| ITGB3BP | 67733 |
| IVL | 16447 |
| IVNS1ABP | 117198 |
| JARID1D | 20592 |
| JMJD1A | 104263 |
| JMJD2C | 76804 |
| JOSD3 | 75316 |
| KAP | 16483 |
| KBTBD3 | 69149 |
| KEG1 | 64697 |
| KERA | 16545 |
| KIF18A | 228421 |
| KLK1 | 16612 |
| KLK1B16 | 16615 |
| KLK1B21 | 16616 |
| KLK1B22 | 13646 |
| KLK1B26 | 16618 |
| KLK1B3 | 18050 |
| KLK1B4 | 18048 |
| KLK1B8 | 16624 |
| KLRA1 | 16627 |
| KLRA17 | 170733 |
| KLRA2 | 16633 |
| KLRA3 | 16634 |
| KLRA5 | 16636 |
| KLRA7 | 16638 |
| KLRB1A | 17057 |
| KLRB1C | 17059 |
| KLRB1F | 232408 |
| KLRC1 | 16641 |
| KLRC3 | 58179 |
| KLRE1 | 243655 |
| KLRK1 | 27007 |
| KPNA1 | 16646 |
| KPNA3 | 16648 |
| KPNA4 | 16649 |
| KRCC1 | 57896 |
| KRTAP12-1 | 16694 |
| KRTAP13-1 | 268905 |
| KRTAP3-2 | 66708 |
| KRTAP3-3 | 66380 |
| KRTAP5-4 | 50775 |
| KRTAP5-5 | 114666 |
| KRTAP9-1 | 16705 |
| KTN1 | 16709 |
| L7RN6 | 67669 |
| LAIR1 | 52855 |
| LAMP2 | 16784 |
| LAMP3 | 239739 |
| LCE1A1 | 67127 |
| LCE1A2 | 73722 |
| LCE1B | 68720 |
| LCE1C | 73719 |
| LCE1D | 69611 |
| LCE1F | 67828 |
| LCE1G | 66195 |
| LCE1H | 67718 |
| LCE1I | 76585 |
| LCE1L | 73730 |
| LCE3A | 545548 |
| LCE3B | 66344 |
| LCE3C | 94060 |
| LCE3F | 69520 |
| LCN12 | 77701 |
| LCN3 | 16820 |
| LCN4 | 16821 |
| LCORL | 209707 |
| LEP | 16846 |
| LILRB3 | 18733 |
| LILRB4 | 14728 |
| LIME1 | 72699 |
| LIPF | 67717 |
| LNP | 69605 |
| LNPEP | 240028 |
| LOH11CR2A | 67776 |
| LPXN | 107321 |
| LRRC19 | 100061 |
| LRRC3B | 218763 |
| LRRC44 | 74435 |
| LRRCC1 | 71710 |
| LRTM1 | 319476 |
| LUZP2 | 233271 |
| LY6A | 110454 |
| LY6F | 17071 |
| LY6I | 57248 |
| LY86 | 17084 |
| LYZL1 | 67328 |
| LZTFL1 | 93730 |
| MACROD2 | 72899 |
| MAGEA1 | 17137 |
| MAGEA2 | 17138 |
| MAGEA3 | 17139 |
| MAGEA4 | 17140 |
| MAGEA5 | 17141 |
| MAGEA6 | 17142 |
| MAGEA8 | 17144 |
| MAGEB1 | 17145 |
| MAGEB3 | 17147 |
| MANEA | 242362 |
| MAOA | 17161 |
| MAOB | 109731 |
| MAP3K7IP3 | 66724 |
| MAPRE1 | 13589 |
| MASTL | 67121 |
| MATR3 | 17184 |
| MBD3L1 | 73503 |
| MBD3L2 | 234988 |
| MBD5 | 109241 |
| MBL1 | 17194 |
| MC2R | 17200 |
| MCART6 | 67062 |
| MCF2 | 109904 |
| MCPT1 | 17224 |
| MCPT2 | 17225 |
| MCPT9 | 17232 |
| MCTP1 | 78771 |
| MCTS1 | 68995 |
| MDC1 | 240087 |
| MED21 | 108098 |
| MED7 | 66213 |
| METTL4 | 76781 |
| METTL5 | 75422 |
| MGA | 29808 |
| MIS12 | 67139 |
| MKKS | 59030 |
| MLL3 | 231051 |
| MME | 17380 |
| MMP10 | 17384 |
| MMP12 | 17381 |
| MMP13 | 17386 |
| MMP1B | 83996 |
| MMP3 | 17392 |
| MMP8 | 17394 |
| MNDA | 381308 |
| MOBKL1B | 232157 |
| MOBKL3 | 19070 |
| MORN2 | 378462 |
| MPA2L | 100702 |
| MPEG1 | 17476 |
| MPHOSPH1 | 240641 |
| MRGPRA1 | 233221 |
| MRGPRA2 | 235712 |
| MS4A1 | 12482 |
| MS4A13 | 73466 |
| MS4A3 | 170813 |
| MS4A4B | 60361 |
| MS4A4C | 64380 |
| MS4A4D | 66607 |
| MS4A6B | 69774 |
| MS4A6D | 68774 |
| MS4A7 | 109225 |
| MTCP1 | 17763 |
| MTF2 | 17765 |
| MTIF2 | 76784 |
| MTPN | 14489 |
| MTX2 | 53375 |
| MUC1 | 17829 |
| MUG1 | 17836 |
| MUG2 | 17837 |
| MUP1 | 17840 |
| MUP2 | 17841 |
| MUP3 | 17842 |
| MUP4 | 17843 |
| MUP5 | 17844 |
| MYBL1 | 17864 |
| NAIP1 | 17940 |
| NAIP2 | 17948 |
| NAIP5 | 17951 |
| NANOG | 71950 |
| NAP1L3 | 54561 |
| NARG1 | 74838 |
| NARG1L | 66897 |
| NARG2 | 93697 |
| NASP | 50927 |
| NAT1 | 17960 |
| NAT3 | 17962 |
| NCAPG2 | 76044 |
| NCL | 17975 |
| NCOA4 | 27057 |
| NCOA6 | 56406 |
| NCR1 | 17086 |
| NDG1 | 368204 |
| NDUFAF1 | 69702 |
| NEK1 | 18004 |
| NEUROD4 | 11923 |
| NFAT5 | 54446 |
| NFATC3 | 18021 |
| NIPA2 | 93790 |
| NKG7 | 72310 |
| NLRP14 | 76858 |
| NLRP4A | 243880 |
| NLRP4B | 210045 |
| NLRP4E | 446099 |
| NLRP5 | 23968 |
| NLRP9A | 233001 |
| NLRP9B | 243874 |
| NLRP9C | 330490 |
| NMD3 | 97112 |
| NNMT | 18113 |
| NOL5 | 55989 |
| NOL8 | 70930 |
| NOSTRIN | 329416 |
| NOX4 | 50490 |
| NPAT | 244879 |
| NPFF | 54615 |
| NPM1 | 18148 |
| NPY6R | 18169 |
| NQO2 | 18105 |
| NSBP1 | 50887 |
| NUFIP2 | 68564 |
| NUP153 | 218210 |
| NUP98 | 269966 |
| NUSAP1 | 108907 |
| NXF3 | 245610 |
| NXF7 | 170722 |
| NXPH2 | 18232 |
| NXT2 | 237082 |
| OBOX3 | 246791 |
| OBOX5 | 252829 |
| OBP1A | 18249 |
| OC90 | 18256 |
| OCIAD2 | 433904 |
| ODAM | 69592 |
| ODF4 | 252868 |
| OGN | 18295 |
| OLFM3 | 229759 |
| OLFR112 | 258096 |
| OLFR140 | 57272 |
| OLFR1417 | 258938 |
| OLFR1507 | 57269 |
| OLFR1508 | 57270 |
| OLFR154 | 27216 |
| OLFR16 | 18313 |
| OLFR160 | 80706 |
| OLFR480 | 56861 |
| OLFR49 | 18348 |
| OLFR558 | 259097 |
| OLFR64 | 18366 |
| OLFR658 | 259051 |
| OLFR66 | 18367 |
| OLFR67 | 18368 |
| OLFR672 | 258755 |
| OLFR68 | 18369 |
| OLFR69 | 18370 |
| OLFR701 | 66786 |
| OLFR73 | 117004 |
| OLFR74 | 117005 |
| OLFR870 | 57251 |
| OLFR976 | 258364 |
| OLR1 | 108078 |
| OMD | 27047 |
| OMG | 18377 |
| OOG2 | 381570 |
| OOG4 | 242737 |
| ORC2L | 18393 |
| ORC3L | 50793 |
| ORC4L | 26428 |
| ORC5L | 26429 |
| OSTB | 330962 |
| OTC | 18416 |
| OTOS | 260301 |
| OTT | 18422 |
| OTTMUSG00000000720 | 435350 |
| OTTMUSG00000000971 | 1E+08 |
| OTTMUSG00000007655 | 242517 |
| OTTMUSG00000008911 | 545677 |
| OTTMUSG00000010009 | 194227 |
| OTTMUSG00000010207 | 545693 |
| OTTMUSG00000010328 | 381569 |
| OTTMUSG00000010433 | 194225 |
| OTTMUSG00000010657 | 666532 |
| OTTMUSG00000011275 | 230398 |
| OTTMUSG00000016406 | 668958 |
| OTTMUSG00000016453 | 434725 |
| OVGP1 | 12659 |
| P2RY10 | 78826 |
| PAIP2 | 67869 |
| PAIP2B | 232164 |
| PAP | 18489 |
| PAPD4 | 100715 |
| PAPOLG | 216578 |
| PBK | 52033 |
| PBSN | 54192 |
| PCM1 | 18536 |
| PCSK1 | 18548 |
| PDCD10 | 56426 |
| PDCL2 | 79455 |
| PDIA2 | 69191 |
| PDS5B | 100710 |
| PET2 | 18630 |
| PGCP | 54381 |
| PGK1 | 18655 |
| PGK2 | 18663 |
| PHCA | 66190 |
| PHEX | 18675 |
| PHF14 | 75725 |
| PHF16 | 382207 |
| PHOSPHO2 | 73373 |
| PIGN | 27392 |
| PILRA | 231805 |
| PIN4 | 69713 |
| PIP | 18716 |
| PIRA2 | 18725 |
| PIRA3 | 18726 |
| PKHD1 | 241035 |
| PKIA | 18767 |
| PLA2G4C | 232889 |
| PLSCR2 | 18828 |
| PLUNC | 18843 |
| PMP2 | 18857 |
| PNMA5 | 385377 |
| PNRC2 | 52830 |
| POF1B | 69693 |
| POLA1 | 18968 |
| POLN | 272158 |
| PPIL3 | 70225 |
| PPM2C | 381511 |
| PPP4R2 | 232314 |
| PPYR1 | 19065 |
| PRAMEL1 | 83491 |
| PRAMEL3 | 83565 |
| PRAMEL4 | 347710 |
| PRAMEL6 | 347711 |
| PRAMEL7 | 347712 |
| PRB1 | 381833 |
| PREI4 | 74182 |
| PRG3 | 53856 |
| PRKDC | 19090 |
| PRL2A1 | 56635 |
| PRL2B1 | 66392 |
| PRL2C2 | 18811 |
| PRL3A1 | 67000 |
| PRL3B1 | 18776 |
| PRL3C1 | 27372 |
| PRL4A1 | 19110 |
| PRL5A1 | 28078 |
| PRL6A1 | 19111 |
| PRL7A1 | 19113 |
| PRL7A2 | 19114 |
| PRL7B1 | 75596 |
| PRL7C1 | 67505 |
| PRL7D1 | 18814 |
| PRL8A1 | 73244 |
| PRL8A2 | 13529 |
| PRL8A6 | 19112 |
| PRL8A8 | 74188 |
| PRL8A9 | 67310 |
| PRLR | 19116 |
| PRPF39 | 328110 |
| PRPMP5 | 381832 |
| PRRG4 | 228413 |
| PRSS1 | 114228 |
| PRSS2 | 22072 |
| PRSS3 | 22073 |
| PRSS7 | 19146 |
| PSG21 | 72242 |
| PSG23 | 56868 |
| PSG28 | 114871 |
| PSMA5 | 26442 |
| PSMD10 | 53380 |
| PSMD14 | 59029 |
| PSME4 | 103554 |
| PSP | 19194 |
| PTEN | 19211 |
| PTGS2 | 19225 |
| PTH | 19226 |
| PTPN4 | 19258 |
| PTPRC | 19264 |
| PUM2 | 80913 |
| PUS3 | 67049 |
| PXMP3 | 19302 |
| PYHIN1 | 236312 |
| R3HDM1 | 226412 |
| RAB5A | 271457 |
| RABGAP1 | 227800 |
| RABGAP1L | 29809 |
| RAD23B | 19359 |
| RAET1D | 56554 |
| RAET1E | 379043 |
| RAG2 | 19374 |
| RANBP2 | 19386 |
| RB1CC1 | 12421 |
| RBBP7 | 245688 |
| RBBP8 | 225182 |
| RBM18 | 67889 |
| RBM43 | 71684 |
| RBM44 | 329207 |
| RBMY1A1 | 19657 |
| RDH7 | 54150 |
| REG1 | 19692 |
| REG3A | 19694 |
| REG3D | 30053 |
| REG3G | 19695 |
| REL | 19696 |
| RFC4 | 106344 |
| RFESD | 218341 |
| RFXDC1 | 320995 |
| RFXDC2 | 319758 |
| RG9MTD1 | 52575 |
| RGN | 19733 |
| RGS1 | 50778 |
| RGS13 | 246709 |
| RGS18 | 64214 |
| RGS5 | 19737 |
| RHAG | 19743 |
| RHBDD3 | 279766 |
| RHOX4A | 664609 |
| RHOX4B | 57737 |
| RHOX4C | 434759 |
| RHOX4D | 664610 |
| RHOX4E | 194856 |
| RHOX4G | 664608 |
| RHOX4H | 636177 |
| RMI1 | 74386 |
| RNASE1 | 19752 |
| RNASE10 | 75019 |
| RNF17 | 30054 |
| RNF185 | 193670 |
| RNF24 | 51902 |
| RNF32 | 56874 |
| ROS1 | 19886 |
| RP1H | 19888 |
| RP23-24J10.7 | 242285 |
| RP23-336F11.32 | 213765 |
| RP23-357I14.1 | 381417 |
| RP23-442I10.2 | 668039 |
| RP2H | 19889 |
| RPE65 | 19892 |
| RPS27L | 67941 |
| RPTN | 20129 |
| RRBP1 | 81910 |
| RTN4 | 68585 |
| RTTN | 246102 |
| S100A1 | 20193 |
| S100A4 | 20198 |
| S100A5 | 20199 |
| S100A6 | 20200 |
| S100A9 | 20202 |
| S100PBP | 74648 |
| SAT1 | 20229 |
| SCGB1A1 | 22287 |
| SCGB3A2 | 117158 |
| SCLT1 | 67161 |
| SCRN3 | 74616 |
| SCYL2 | 213326 |
| SEBOX | 18292 |
| SELL | 20343 |
| SENP5 | 320213 |
| SEPP1 | 20363 |
| SERF1 | 20365 |
| SERPINA10 | 217847 |
| SERPINA3C | 16625 |
| SERPINA3K | 20714 |
| SERPINA3M | 20717 |
| SERPINA3N | 20716 |
| SERPINA6 | 12401 |
| SERPINB11 | 66957 |
| SERPINB12 | 71869 |
| SERPINB1A | 66222 |
| SERPINB1B | 282663 |
| SERPINB2 | 18788 |
| SERPINB3A | 20248 |
| SERPINB3B | 383548 |
| SERPINB5 | 20724 |
| SERPINB6A | 20719 |
| SERPINB6B | 20708 |
| SERPINB9 | 20723 |
| SERPINB9B | 20706 |
| SERPINB9C | 20707 |
| SERPINB9D | 20726 |
| SERPINB9E | 20710 |
| SERPINB9F | 20709 |
| SERPINB9G | 93806 |
| SERPING1 | 12258 |
| SERPINI1 | 20713 |
| SERPINI2 | 67931 |
| SETD2 | 235626 |
| SETDB2 | 239122 |
| SETX | 269254 |
| SFTPD | 20390 |
| SGOL2 | 68549 |
| SH3BGR | 50795 |
| SHCBP1 | 20419 |
| SIGLECH | 233274 |
| SIRPB1 | 320832 |
| SIX6OS1 | 75801 |
| SKP1A | 21402 |
| SLAMF6 | 30925 |
| SLC10A2 | 20494 |
| SLC10A3 | 214601 |
| SLC13A1 | 55961 |
| SLC16A4 | 229699 |
| SLC17A1 | 20504 |
| SLC17A3 | 105355 |
| SLC18A1 | 110877 |
| SLC22A9 | 207151 |
| SLC25A40 | 319653 |
| SLC28A2 | 269346 |
| SLC31A2 | 20530 |
| SLC35A3 | 229782 |
| SLC35A5 | 74102 |
| SLC39A12 | 277468 |
| SLC40A1 | 53945 |
| SLC5A12 | 241612 |
| SLC5A7 | 63993 |
| SLC6A14 | 56774 |
| SLC7A12 | 140918 |
| SLCO1A1 | 28248 |
| SLCO1A4 | 28250 |
| SLCO1A6 | 28254 |
| SLCO1B2 | 28253 |
| SLCO6B1 | 67854 |
| SLCO6C1 | 74441 |
| SLFN1 | 20555 |
| SLFN10 | 237887 |
| SLFN8 | 276950 |
| SLFN9 | 237886 |
| SLITRK4 | 245446 |
| SLITRK6 | 239250 |
| SMC1B | 140557 |
| SMC2 | 14211 |
| SMGC | 223809 |
| SMR1 | 20599 |
| SMR2 | 20600 |
| SNAP23 | 20619 |
| SNCA | 20617 |
| SNRPB2 | 20639 |
| SNRPN | 20646 |
| SNUPN | 66069 |
| SNX16 | 74718 |
| SP1 | 20683 |
| SPATA1 | 70951 |
| SPC25 | 66442 |
| SPEER3 | 71026 |
| SPEER4F | 70935 |
| SPINK10 | 328971 |
| SPINK12 | 78242 |
| SPINK4 | 20731 |
| SPOPL | 76857 |
| SPRR1A | 20753 |
| SPRR1B | 20754 |
| SPRR2D | 20758 |
| SPRR2H | 20762 |
| SPRR2I | 20763 |
| SPRR2K | 20765 |
| SPRR3 | 20766 |
| SRD5A2L2 | 243078 |
| SRP54A | 24067 |
| SRR | 27364 |
| SRY | 21674 |
| SSB | 20823 |
| ST18 | 240690 |
| STAM | 20844 |
| STAT4 | 20849 |
| STFA2L1 | 268885 |
| STFA3 | 20863 |
| STK31 | 77485 |
| STRBP | 20744 |
| STYK1 | 243659 |
| SUCNR1 | 84112 |
| SUHW4 | 235469 |
| SULT1B1 | 56362 |
| SULT1C1 | 20888 |
| SULT1C2 | 69083 |
| SULT1D1 | 53315 |
| SULT1E1 | 20860 |
| SULT3A1 | 57430 |
| SVA | 20939 |
| SVAL1 | 71578 |
| SVAL2 | 84543 |
| SVS3A | 64335 |
| SVS3B | 329557 |
| SYCP1 | 20957 |
| SYCP2 | 320558 |
| SYCP3 | 20962 |
| SYTL2 | 83671 |
| TAS2R105 | 57252 |
| TAS2R108 | 57253 |
| TATDN1 | 69694 |
| TBC1D8B | 245638 |
| TBP | 21374 |
| TBX22 | 245572 |
| TC2N | 74413 |
| TCEA1 | 21399 |
| TCEB1 | 67923 |
| TCF20 | 21411 |
| TCHHL1 | 71325 |
| TCL1B1 | 27379 |
| TCL1B4 | 27380 |
| TEX10 | 269536 |
| TEX11 | 83558 |
| TEX12 | 66654 |
| TEX15 | 104271 |
| TEX16 | 83556 |
| TEX19 | 73679 |
| TEX24 | 541463 |
| TFRC | 22042 |
| TG | 21819 |
| TGS1 | 116940 |
| TGTP | 21822 |
| THEM5 | 66198 |
| THOC1 | 225160 |
| THOC2 | 331401 |
| THUMPD3 | 14911 |
| TIMM9 | 30056 |
| TIPARP | 99929 |
| TIPIN | 66131 |
| TLR1 | 21897 |
| TLR13 | 279572 |
| TLR4 | 21898 |
| TLR6 | 21899 |
| TLR7 | 170743 |
| TLR8 | 170744 |
| TM4SF20 | 66261 |
| TMCO5 | 67356 |
| TMEM126A | 66271 |
| TMEM14A | 75712 |
| TMEM161B | 72745 |
| TMEM177 | 66343 |
| TMEM68 | 72098 |
| TMEM71 | 213068 |
| TMEM77 | 67171 |
| TMEM80 | 71448 |
| TMEM86B | 68255 |
| TNFSF10 | 22035 |
| TOP2B | 21974 |
| TOX4 | 268741 |
| TPBPA | 21984 |
| TPBPB | 116913 |
| TPRKB | 69786 |
| TRAF3IP3 | 215243 |
| TREM1 | 58217 |
| TRHR | 22045 |
| TRIM12 | 76681 |
| TRIM13 | 66597 |
| TRIM23 | 81003 |
| TRIM30 | 20128 |
| TRIM40 | 195359 |
| TRIM59 | 66949 |
| TRNT1 | 70047 |
| TRO | 56191 |
| TRP53INP1 | 60599 |
| TRPM7 | 58800 |
| TRY10 | 436522 |
| TRY4 | 22074 |
| TSGA13 | 116732 |
| TTBK2 | 140810 |
| TXNDC8 | 67402 |
| UBE1L2 | 231380 |
| UBE2D3 | 66105 |
| UBE2E3 | 22193 |
| UBE2T | 67196 |
| UBE2U | 381534 |
| UBE2V2 | 70620 |
| UBE3A | 22215 |
| UBLCP1 | 79560 |
| UBOX5 | 140629 |
| UBQLN3 | 244178 |
| UBQLNL | 244179 |
| UBXD4 | 217379 |
| UCHL5 | 56207 |
| UGP2 | 216558 |
| UGT2A1 | 94215 |
| UGT2A2 | 552899 |
| UGT2B1 | 71773 |
| UGT2B34 | 100727 |
| UGT2B35 | 243085 |
| UGT2B36 | 231396 |
| UGT2B37 | 112417 |
| UGT2B38 | 100559 |
| UGT2B5 | 22238 |
| UGT3A1 | 105887 |
| UGT3A2 | 223337 |
| UGT8A | 22239 |
| UIMC1 | 20184 |
| USMG5 | 66477 |
| USP14 | 59025 |
| USP26 | 83563 |
| USP29 | 57775 |
| USP32 | 237898 |
| USP37 | 319651 |
| USP40 | 227334 |
| USP9X | 22284 |
| USP9Y | 107868 |
| USPL1 | 231915 |
| UTP14A | 72554 |
| UTP14B | 195434 |
| UTX | 22289 |
| UTY | 22290 |
| V1RA1 | 22296 |
| V1RA2 | 22297 |
| V1RA3 | 113845 |
| V1RA4 | 113846 |
| V1RA9 | 113851 |
| V1RB1 | 113852 |
| V1RB10 | 113867 |
| V1RB2 | 24112 |
| V1RB3 | 113853 |
| V1RB4 | 113854 |
| V1RB7 | 113855 |
| V1RB8 | 113856 |
| V1RC1 | 113858 |
| V1RC2 | 113859 |
| V1RC3 | 113860 |
| V1RC5 | 113862 |
| V1RC6 | 113863 |
| V1RC7 | 113864 |
| V1RC8 | 113865 |
| V1RD1 | 81017 |
| V1RD14 | 81011 |
| V1RD3 | 81015 |
| V1RD4 | 81014 |
| V1RD6 | 81013 |
| V1RD7 | 81012 |
| V1RD9 | 81010 |
| VCAN | 13003 |
| VDAC3 | 22335 |
| VMN2R1 | 56544 |
| VMN2R26 | 56552 |
| VMN2R42 | 22310 |
| VMN2R49 | 625605 |
| VMN2R84 | 625068 |
| VPS13A | 271564 |
| VPS35 | 65114 |
| VPS54 | 245944 |
| VRK2 | 69922 |
| VSIG1 | 78789 |
| VSIG4 | 278180 |
| WAC | 225131 |
| WAPAL | 218914 |
| WDR44 | 72404 |
| WDR61 | 66317 |
| WEE2 | 381759 |
| WFDC6A | 209351 |
| WWP1 | 107568 |
| XCL1 | 16963 |
| XCR1 | 23832 |
| XLR | 22441 |
| XLR3B | 574437 |
| XLR3C | 22446 |
| XLR4A | 434794 |
| XLR4B | 27083 |
| XLR4C | 72891 |
| XLR5A | 574438 |
| XMR | 382277 |
| XPO1 | 103573 |
| XRCC4 | 108138 |
| YES1 | 22612 |
| YIPF7 | 75581 |
| YTHDF3 | 229096 |
| ZBTB26 | 320633 |
| ZBTB33 | 56805 |
| ZCCHC11 | 230594 |
| ZFAND5 | 22682 |
| ZFML | 18139 |
| ZFP101 | 22643 |
| ZFP105 | 22646 |
| ZFP110 | 65020 |
| ZFP113 | 56314 |
| ZFP119 | 104349 |
| ZFP120 | 104348 |
| ZFP160 | 224585 |
| ZFP182 | 319535 |
| ZFP192 | 93681 |
| ZFP2 | 22678 |
| ZFP235 | 56525 |
| ZFP26 | 22688 |
| ZFP318 | 57908 |
| ZFP322A | 218100 |
| ZFP329 | 67230 |
| ZFP35 | 22694 |
| ZFP352 | 236537 |
| ZFP353 | 234203 |
| ZFP369 | 170936 |
| ZFP386 | 56220 |
| ZFP397 | 69256 |
| ZFP40 | 22700 |
| ZFP418 | 232854 |
| ZFP426 | 235028 |
| ZFP438 | 240186 |
| ZFP449 | 78619 |
| ZFP458 | 238690 |
| ZFP51 | 22709 |
| ZFP518 | 72672 |
| ZFP52 | 22710 |
| ZFP53 | 24132 |
| ZFP54 | 22712 |
| ZFP560 | 434377 |
| ZFP583 | 213011 |
| ZFP59 | 22717 |
| ZFP595 | 218314 |
| ZFP597 | 71063 |
| ZFP606 | 67370 |
| ZFP62 | 22720 |
| ZFP655 | 72611 |
| ZFP664 | 269704 |
| ZFP677 | 210503 |
| ZFP68 | 24135 |
| ZFP706 | 68036 |
| ZFP708 | 432769 |
| ZFP711 | 245595 |
| ZFP715 | 69930 |
| ZFP748 | 212276 |
| ZFP75 | 244713 |
| ZFP758 | 224598 |
| ZFP760 | 240034 |
| ZFP770 | 228491 |
| ZFP78 | 330463 |
| ZFP800 | 627049 |
| ZFP804A | 241514 |
| ZFP81 | 224694 |
| ZFP817 | 238693 |
| ZFP85-RS1 | 22746 |
| ZFY1 | 22767 |
| ZFY2 | 22768 |
| ZFYVE16 | 218441 |
| ZFYVE9 | 230597 |
| ZHX1 | 22770 |
| ZHX3 | 320799 |
| ZIK1 | 22775 |
| ZKSCAN6 | 52712 |
| ZMAT1 | 215693 |
| ZP2 | 22787 |
| ZWILCH | 68014 |
