# Supplementary material for: Patterns of Oligonucleotide Sequences in Viral and Host Cell RNA Identify Mediators of the Host Innate Immune System
Source: PLoS One. 2009 Jun 18;4(6):e5969. doi: 10.1371/journal.pone.0005969 (PMC2694999; doi:10.1371/journal.pone.0005969)
Supplement: Table S10 — The lowest genes in the human genome, by the same criteria as in Table S9. (1.05 MB DOC) [file pone.0005969.s010.doc]

| Human Gene Name | Human Entrez ID |
| --- | --- |
| AADAT | 51166 |
| AASDH | 132949 |
| ABCA10 | 10349 |
| ABCA5 | 23461 |
| ABCA6 | 23460 |
| ABHD13 | 84945 |
| ACAT2 | 39 |
| ACCN5 | 51802 |
| ACE2 | 59272 |
| ACOT6 | 641372 |
| ACRV1 | 56 |
| ACSL4 | 2182 |
| ACVR2A | 92 |
| ADAD1 | 132612 |
| ADAL | 161823 |
| ADAM18 | 8749 |
| ADAM2 | 2515 |
| ADAM20 | 8748 |
| ADAM21 | 8747 |
| ADAM28 | 10863 |
| ADH4 | 127 |
| ADK | 132 |
| AFM | 173 |
| AFP | 174 |
| AFTPH | 54812 |
| AGL | 178 |
| AGR2 | 10551 |
| AGR3 | 155465 |
| AHNAK | 79026 |
| AHSA2 | 130872 |
| AKAP4 | 8852 |
| AKAP7 | 9465 |
| AKAP9 | 10142 |
| AKT3 | 10000 |
| ALG10B | 144245 |
| ALG6 | 29929 |
| ALKBH8 | 91801 |
| ALMS1 | 7840 |
| ALS2CR2 | 55437 |
| ALS2CR8 | 79800 |
| AMELY | 266 |
| ANAPC10 | 10393 |
| ANAPC13 | 25847 |
| ANGPTL1 | 9068 |
| ANGPTL3 | 27329 |
| ANKRA2 | 57763 |
| ANKRD12 | 23253 |
| ANKRD26 | 22852 |
| ANKRD30A | 91074 |
| ANKRD32 | 84250 |
| ANKRD34B | 340120 |
| ANXA5 | 308 |
| AP3S1 | 1176 |
| AP4E1 | 23431 |
| APAF1 | 317 |
| APC | 324 |
| APCS | 325 |
| APOBEC4 | 403314 |
| ARF4 | 378 |
| ARFGEF1 | 10565 |
| ARFIP1 | 27236 |
| ARHGAP11A | 9824 |
| ARHGAP29 | 9411 |
| ARHGAP5 | 394 |
| ARID4A | 5926 |
| ARID4B | 51742 |
| ARL17 | 641522 |
| ARL4A | 10124 |
| ARL5A | 26225 |
| ARL5B | 221079 |
| ARL6 | 84100 |
| ARNTL2 | 56938 |
| ARPC3 | 10094 |
| ART4 | 420 |
| ASB17 | 127247 |
| ASPH | 444 |
| ASPN | 54829 |
| ASTE1 | 28990 |
| ATAD5 | 79915 |
| ATG3 | 64422 |
| ATG4C | 84938 |
| ATG5 | 9474 |
| ATM | 472 |
| ATP11B | 23200 |
| ATP11C | 286410 |
| ATP2C1 | 27032 |
| ATP5H | 10476 |
| ATP6V1C1 | 528 |
| ATP7A | 538 |
| ATPBD4 | 89978 |
| ATRX | 546 |
| AURKA | 6790 |
| AZI2 | 64343 |
| AZIN1 | 51582 |
| B3GALNT1 | 8706 |
| BANK1 | 55024 |
| BAZ2B | 29994 |
| BBS12 | 166379 |
| BBS4 | 585 |
| BBS7 | 55212 |
| BCAP29 | 55973 |
| BCHE | 590 |
| BCL2L11 | 10018 |
| BCL2L14 | 79370 |
| BET1 | 10282 |
| BHLHB9 | 80823 |
| BIRC2 | 329 |
| BMI1 | 648 |
| BNIP2 | 663 |
| BRCA1 | 672 |
| BRCA2 | 675 |
| BRDT | 676 |
| BRIP1 | 83990 |
| BTF3L4 | 91408 |
| BTLA | 151888 |
| BZRPL1 | 222642 |
| C10ORF107 | 219621 |
| C10ORF118 | 55088 |
| C10ORF28 | 27291 |
| C10ORF4 | 118924 |
| C10ORF6 | 55719 |
| C10ORF68 | 79741 |
| C10ORF78 | 119392 |
| C10ORF81 | 79949 |
| C10ORF84 | 63877 |
| C10ORF96 | 374355 |
| C11ORF46 | 120534 |
| C11ORF54 | 28970 |
| C11ORF57 | 55216 |
| C11ORF65 | 160140 |
| C11ORF67 | 28971 |
| C11ORF73 | 51501 |
| C11ORF74 | 119710 |
| C11ORF82 | 220042 |
| C12ORF23 | 90488 |
| C12ORF29 | 91298 |
| C12ORF31 | 84298 |
| C12ORF35 | 55196 |
| C12ORF4 | 57102 |
| C12ORF48 | 55010 |
| C13ORF31 | 144811 |
| C13ORF34 | 79866 |
| C14ORF105 | 55195 |
| C14ORF106 | 55320 |
| C14ORF108 | 55745 |
| C14ORF39 | 317761 |
| C14ORF45 | 80127 |
| C15ORF29 | 79768 |
| C16ORF65 | 255762 |
| C1D | 10438 |
| C1GALT1 | 56913 |
| C1GALT1C1 | 29071 |
| C1ORF103 | 55791 |
| C1ORF112 | 55732 |
| C1ORF125 | 126859 |
| C1ORF156 | 92342 |
| C1ORF176 | 64789 |
| C1ORF182 | 128229 |
| C1ORF192 | 257177 |
| C1ORF212 | 113444 |
| C1ORF218 | 54530 |
| C1ORF26 | 54823 |
| C1ORF41 | 51668 |
| C1ORF74 | 148304 |
| C21ORF51 | 54065 |
| C21ORF91 | 54149 |
| C2ORF25 | 27249 |
| C2ORF44 | 80304 |
| C2ORF67 | 151050 |
| C3ORF14 | 57415 |
| C3ORF17 | 25871 |
| C3ORF23 | 285343 |
| C4ORF15 | 79441 |
| C4ORF28 | 133015 |
| C4ORF33 | 132321 |
| C4ORF34 | 201895 |
| C4ORF35 | 85438 |
| C5 | 727 |
| C5ORF3 | 10827 |
| C5ORF32 | 84418 |
| C5ORF41 | 153222 |
| C5ORF5 | 51306 |
| C6ORF10 | 10665 |
| C6ORF167 | 253714 |
| C6ORF182 | 285753 |
| C6ORF211 | 79624 |
| C6ORF58 | 352999 |
| C6ORF60 | 79632 |
| C7ORF25 | 79020 |
| C8ORF34 | 116328 |
| C8ORF70 | 51101 |
| C9ORF11 | 54586 |
| C9ORF128 | 392307 |
| C9ORF152 | 401546 |
| C9ORF153 | 389766 |
| C9ORF46 | 55848 |
| C9ORF72 | 203228 |
| C9ORF95 | 54981 |
| CA1 | 759 |
| CABYR | 26256 |
| CACYBP | 27101 |
| CALCRL | 10203 |
| CALM2 | 805 |
| CAPRIN2 | 65981 |
| CAPS2 | 84698 |
| CASC5 | 57082 |
| CASP8 | 841 |
| CBWD2 | 150472 |
| CBWD3 | 445571 |
| CBX3 | 11335 |
| CCBL2 | 56267 |
| CCDC104 | 112942 |
| CCDC109B | 55013 |
| CCDC110 | 256309 |
| CCDC111 | 201973 |
| CCDC122 | 160857 |
| CCDC125 | 202243 |
| CCDC132 | 55610 |
| CCDC23 | 374969 |
| CCDC29 | 728788 |
| CCDC38 | 120935 |
| CCDC46 | 201134 |
| CCDC54 | 84692 |
| CCDC55 | 84081 |
| CCDC66 | 285331 |
| CCDC68 | 80323 |
| CCDC7 | 221016 |
| CCDC73 | 493860 |
| CCDC75 | 253635 |
| CCDC83 | 220047 |
| CCDC88A | 55704 |
| CCDC91 | 55297 |
| CCL13 | 6357 |
| CCL24 | 6369 |
| CCL7 | 6354 |
| CCL8 | 6355 |
| CCNB3 | 85417 |
| CCNC | 892 |
| CCNE2 | 9134 |
| CCNG1 | 900 |
| CCNG2 | 901 |
| CCNI | 10983 |
| CCPG1 | 9236 |
| CCR8 | 1237 |
| CCRL1 | 51554 |
| CD109 | 135228 |
| CD160 | 11126 |
| CD180 | 4064 |
| CD274 | 29126 |
| CD36 | 948 |
| CD69 | 969 |
| CD96 | 10225 |
| CDC2 | 983 |
| CDC27 | 996 |
| CDC6 | 990 |
| CDC7 | 8317 |
| CDKL2 | 8999 |
| CENPF | 1063 |
| CENPI | 2491 |
| CENPK | 64105 |
| CENPL | 91687 |
| CENPQ | 55166 |
| CEP152 | 22995 |
| CEP63 | 80254 |
| CEP70 | 80321 |
| CETN3 | 1070 |
| CFH | 3075 |
| CFHR2 | 3080 |
| CFHR3 | 10878 |
| CFHR4 | 10877 |
| CHD1 | 1105 |
| CHEK1 | 1111 |
| CHI3L2 | 1117 |
| CHML | 1122 |
| CHORDC1 | 26973 |
| CHST9 | 83539 |
| CLCA2 | 9635 |
| CLCA3 | 9629 |
| CLCA4 | 22802 |
| CLDN2 | 9075 |
| CLEC2D | 29121 |
| CLEC4A | 50856 |
| CLEC4E | 26253 |
| CLEC6A | 93978 |
| CLGN | 1047 |
| CLOCK | 9575 |
| CLRN1 | 7401 |
| CLSPN | 63967 |
| CNOT10 | 25904 |
| CNOT8 | 9337 |
| CNTN6 | 27255 |
| COL10A1 | 1300 |
| COL24A1 | 255631 |
| COL4A5 | 1287 |
| COPB2 | 9276 |
| COPS2 | 9318 |
| COX7B2 | 170712 |
| CP110 | 9738 |
| CPLX4 | 339302 |
| CPNE3 | 8895 |
| CPSF2 | 53981 |
| CRB1 | 23418 |
| CREM | 1390 |
| CRIPT | 9419 |
| CRISP2 | 7180 |
| CRISP3 | 10321 |
| CRYZL1 | 9946 |
| CSE1L | 1434 |
| CSN2 | 1447 |
| CST9L | 128821 |
| CTAGE5 | 4253 |
| CTSK | 1513 |
| CTSL1 | 1514 |
| CTSL3 | 392360 |
| CWF19L1 | 55280 |
| CWF19L2 | 143884 |
| CXCL10 | 3627 |
| CXCR6 | 10663 |
| CXORF21 | 80231 |
| CXORF41 | 139212 |
| CYB5R4 | 51167 |
| CYLC1 | 1538 |
| CYORF15B | 84663 |
| CYP2C8 | 1558 |
| CYP2C9 | 1559 |
| CYP39A1 | 51302 |
| CYP3A4 | 1576 |
| CYP3A43 | 64816 |
| CYSLTR1 | 10800 |
| CYSLTR2 | 57105 |
| DARC | 2532 |
| DAZ2 | 57055 |
| DAZL | 1618 |
| DBF4 | 10926 |
| DCLRE1A | 9937 |
| DCLRE1C | 64421 |
| DCUN1D5 | 84259 |
| DDX1 | 1653 |
| DDX52 | 11056 |
| DDX58 | 23586 |
| DDX59 | 83479 |
| DEFA6 | 1671 |
| DEFB1 | 1672 |
| DEFB107A | 245910 |
| DEFB116 | 245930 |
| DEFB121 | 245934 |
| DEFB4 | 1673 |
| DEPDC7 | 91614 |
| DKFZP667G2110 | 131544 |
| DLC1 | 10395 |
| DLD | 1738 |
| DLG7 | 9787 |
| DMXL1 | 1657 |
| DNAJB7 | 150353 |
| DNAJB9 | 4189 |
| DNAJC10 | 54431 |
| DPH4 | 120526 |
| DPH5 | 51611 |
| DPP4 | 1803 |
| DSEL | 92126 |
| DST | 667 |
| DSTN | 11034 |
| DTWD1 | 56986 |
| DUS4L | 11062 |
| DUSP19 | 142679 |
| DYDC2 | 84332 |
| DZIP3 | 9666 |
| EBAG9 | 9166 |
| ECHDC1 | 55862 |
| ECT2 | 1894 |
| EDEM3 | 80267 |
| EEA1 | 8411 |
| EFCAB1 | 79645 |
| EFCAB3 | 146779 |
| EFTUD1 | 79631 |
| EIF1AY | 9086 |
| EIF5B | 9669 |
| ELF2 | 1998 |
| ELK4 | 2005 |
| ELMOD2 | 255520 |
| ELP2 | 55250 |
| ELTD1 | 64123 |
| EMCN | 51705 |
| ENAM | 10117 |
| ENPP3 | 5169 |
| ENPP4 | 22875 |
| ENTPD5 | 957 |
| EPGN | 255324 |
| EPS15 | 2060 |
| ERAP2 | 64167 |
| ERBB2IP | 55914 |
| ERCC6L | 54821 |
| ERCC8 | 1161 |
| ERGIC2 | 51290 |
| ERMN | 57471 |
| ESCO1 | 114799 |
| ESCO2 | 157570 |
| ESD | 2098 |
| ESF1 | 51575 |
| ETFDH | 2110 |
| EVI2B | 2124 |
| EXDL1 | 161829 |
| EXOC1 | 55763 |
| EXOC6 | 54536 |
| EXPH5 | 23086 |
| EXTL2 | 2135 |
| EYA3 | 2140 |
| EYA4 | 2070 |
| F2RL2 | 2151 |
| F5 | 2153 |
| F8 | 2157 |
| FABP5 | 2171 |
| FABP7 | 2173 |
| FAM111A | 63901 |
| FAM126A | 84668 |
| FAM134B | 54463 |
| FAM135A | 57579 |
| FAM35A | 54537 |
| FAM3C | 10447 |
| FAM48A | 55578 |
| FAM49B | 51571 |
| FAM55D | 54827 |
| FAM60A | 58516 |
| FAM72A | 729533 |
| FAM72B | 653820 |
| FAM82A | 151393 |
| FAM9C | 171484 |
| FANCD2 | 2177 |
| FANCI | 55215 |
| FAS | 355 |
| FASTKD2 | 22868 |
| FBXL4 | 26235 |
| FBXL5 | 26234 |
| FBXO11 | 80204 |
| FBXO30 | 84085 |
| FBXO43 | 286151 |
| FCER1A | 2205 |
| FCGR1B | 2210 |
| FCRL2 | 79368 |
| FER | 2241 |
| FGD4 | 121512 |
| FGF7 | 2252 |
| FGFBP1 | 9982 |
| FGG | 2266 |
| FIGNL1 | 63979 |
| FLJ11171 | 55783 |
| FLJ11506 | 79719 |
| FLJ20184 | 54848 |
| FLJ21511 | 80157 |
| FLJ35767 | 400629 |
| FMO4 | 2329 |
| FMO5 | 2330 |
| FNDC3A | 22862 |
| FOXN2 | 3344 |
| FPGT | 8790 |
| FRRS1 | 391059 |
| FSHR | 2492 |
| FSIP1 | 161835 |
| FSTL5 | 56884 |
| FXYD8 | 406875 |
| GABPB2 | 2553 |
| GABRA2 | 2555 |
| GABRG1 | 2565 |
| GALNT1 | 2589 |
| GANC | 2595 |
| GARNL1 | 253959 |
| GART | 2618 |
| GBP7 | 388646 |
| GC | 2638 |
| GCC2 | 9648 |
| GCET2 | 257144 |
| GEN1 | 348654 |
| GFM2 | 84340 |
| GGPS1 | 9453 |
| GHR | 2690 |
| GIMAP2 | 26157 |
| GIN1 | 54826 |
| GJA10 | 84694 |
| GJA9 | 81025 |
| GJB7 | 375519 |
| GKAP1 | 80318 |
| GKN2 | 200504 |
| GLCE | 26035 |
| GLMN | 11146 |
| GLRB | 2743 |
| GLYAT | 10249 |
| GLYATL2 | 219970 |
| GMEB1 | 10691 |
| GMNN | 51053 |
| GNG12 | 55970 |
| GNL3 | 26354 |
| GNPDA2 | 132789 |
| GNPNAT1 | 64841 |
| GNRHR | 2798 |
| GOLGA4 | 2803 |
| GPR1 | 2825 |
| GPR110 | 266977 |
| GPR155 | 151556 |
| GPR22 | 2845 |
| GPRASP1 | 9737 |
| GPRC6A | 222545 |
| GPX6 | 257202 |
| GSTA1 | 2938 |
| GSTA2 | 2939 |
| GSTA5 | 221357 |
| GSTCD | 79807 |
| GTF2A1 | 2957 |
| GTF2H1 | 2965 |
| GTPBP10 | 85865 |
| GUCA1C | 9626 |
| HBB | 3043 |
| HBG1 | 3047 |
| HBG2 | 3048 |
| HBP1 | 26959 |
| HEMGN | 55363 |
| HERC3 | 8916 |
| HERC4 | 26091 |
| HIBCH | 26275 |
| HIG2 | 29923 |
| HIPK3 | 10114 |
| HLTF | 6596 |
| HMG1L1 | 10357 |
| HMGN2 | 3151 |
| HMMR | 3161 |
| HNMT | 3176 |
| HORMAD1 | 84072 |
| HPS5 | 11234 |
| HRH4 | 59340 |
| HSD11B1 | 3290 |
| HSDL2 | 84263 |
| HSF2 | 3298 |
| HSFY1 | 86614 |
| HSFY2 | 159119 |
| HSPD1 | 3329 |
| HTR1F | 3355 |
| IBTK | 25998 |
| ICA1L | 130026 |
| ICOS | 29851 |
| IFI16 | 3428 |
| IFIT1 | 3434 |
| IFIT1L | 439996 |
| IFIT3 | 3437 |
| IFNA1 | 3439 |
| IFNA10 | 3446 |
| IFNA13 | 3447 |
| IFNA14 | 3448 |
| IFNA16 | 3449 |
| IFNA17 | 3451 |
| IFNA2 | 3440 |
| IFNA21 | 3452 |
| IFNA4 | 3441 |
| IFNA5 | 3442 |
| IFNA6 | 3443 |
| IFNA7 | 3444 |
| IFNA8 | 3445 |
| IFNB1 | 3456 |
| IFT80 | 57560 |
| IFT81 | 28981 |
| IFT88 | 8100 |
| IGSF1 | 3547 |
| IL12RB2 | 3595 |
| IL15 | 3600 |
| IL1F8 | 27177 |
| IL1R1 | 3554 |
| IL1RAPL2 | 26280 |
| IL2 | 3558 |
| IL21 | 59067 |
| IL23A | 51561 |
| IL33 | 90865 |
| IL6ST | 3572 |
| IMPA1 | 3612 |
| INTS12 | 57117 |
| INTS7 | 25896 |
| IPO11 | 51194 |
| IPO7 | 10527 |
| IQCB1 | 9657 |
| IQUB | 154865 |
| IRAK4 | 51135 |
| ITGB3BP | 23421 |
| IVL | 3713 |
| IVNS1ABP | 10625 |
| JAK2 | 3717 |
| JOSD3 | 79101 |
| KBTBD3 | 143879 |
| KERA | 11081 |
| KIAA0256 | 9728 |
| KIAA0319L | 79932 |
| KIAA0372 | 9652 |
| KIAA1128 | 54462 |
| KIAA1324L | 222223 |
| KIAA1333 | 55632 |
| KIAA1377 | 57562 |
| KIAA1432 | 57589 |
| KIAA1586 | 57691 |
| KIAA1712 | 80817 |
| KIAA1715 | 80856 |
| KIAA1797 | 54914 |
| KIAA2018 | 205717 |
| KIF11 | 3832 |
| KIF15 | 56992 |
| KIFAP3 | 22920 |
| KITLG | 4254 |
| KLHL23 | 151230 |
| KLRA1 | 10748 |
| KLRB1 | 3820 |
| KLRC1 | 3821 |
| KLRC3 | 3823 |
| KLRC4 | 8302 |
| KLRG1 | 10219 |
| KPNA1 | 3836 |
| KPNA3 | 3839 |
| KPNA4 | 3840 |
| KPNA5 | 3841 |
| KRAS | 3845 |
| KRIT1 | 889 |
| KRTAP13-3 | 337960 |
| KRTAP5-8 | 57830 |
| KRTAP9-2 | 83899 |
| KRTAP9-3 | 83900 |
| KRTAP9-8 | 83901 |
| KTN1 | 3895 |
| LALBA | 3906 |
| LAMP2 | 3920 |
| LARP4 | 113251 |
| LARP7 | 51574 |
| LCA5L | 150082 |
| LCE1B | 353132 |
| LCE1E | 353135 |
| LCE5A | 254910 |
| LDHC | 3948 |
| LEPR | 3953 |
| LIG4 | 3981 |
| LIN54 | 132660 |
| LINS1 | 55180 |
| LIPF | 8513 |
| LIPI | 149998 |
| LIPJ | 142910 |
| LIPT1 | 51601 |
| LNPEP | 4012 |
| LOC162993 | 162993 |
| LOC253012 | 253012 |
| LOC286187 | 286187 |
| LOC440087 | 440087 |
| LOC645843 | 645843 |
| LOC728957 | 728957 |
| LPAR4 | 2846 |
| LPGAT1 | 9926 |
| LRRC19 | 64922 |
| LRRC39 | 127495 |
| LRRC44 | 127255 |
| LRRCC1 | 85444 |
| LRRK2 | 120892 |
| LRRN3 | 54674 |
| LTB | 4050 |
| LTV1 | 84946 |
| LUM | 4060 |
| LY96 | 23643 |
| LYRM7 | 90624 |
| LZIC | 84328 |
| LZTFL1 | 54585 |
| MAGEA10 | 4109 |
| MAGEA11 | 4110 |
| MAP9 | 79884 |
| MAPK6 | 5597 |
| MAT2B | 27430 |
| MBL2 | 4153 |
| MCF2 | 4168 |
| MCFD2 | 90411 |
| MCOLN3 | 55283 |
| MCTS1 | 28985 |
| MDM2 | 4193 |
| MED1 | 5469 |
| MED13 | 9969 |
| MELK | 9833 |
| METT5D1 | 196074 |
| METTL4 | 64863 |
| METTL6 | 131965 |
| MGAT4C | 25834 |
| MGC13057 | 84281 |
| MGC16169 | 93627 |
| MGC50559 | 254013 |
| MIA2 | 117153 |
| MIER1 | 57708 |
| MKKS | 8195 |
| MLH3 | 27030 |
| MLZE | 56169 |
| MMP10 | 4319 |
| MOBKL1A | 92597 |
| MOBKL3 | 25843 |
| MON2 | 23041 |
| MORC1 | 27136 |
| MPHOSPH1 | 9585 |
| MPHOSPH6 | 10200 |
| MPP6 | 51678 |
| MRPL30 | 51263 |
| MRPS36 | 92259 |
| MS4A1 | 931 |
| MS4A2 | 2206 |
| MS4A3 | 932 |
| MS4A4A | 51338 |
| MS4A6A | 64231 |
| MS4A6E | 245802 |
| MS4A7 | 58475 |
| MSH4 | 4438 |
| MT1A | 4489 |
| MT4 | 84560 |
| MTBP | 27085 |
| MTERFD1 | 51001 |
| MTERFD3 | 80298 |
| MTF2 | 22823 |
| MTIF2 | 4528 |
| MTMR8 | 55613 |
| MTPN | 136319 |
| MTX2 | 10651 |
| MYL1 | 4632 |
| MYOZ2 | 51778 |
| N4BP2 | 55728 |
| N4BP2L2 | 10443 |
| NAMPT | 10135 |
| NANOG | 79923 |
| NAP1L1 | 4673 |
| NARG1 | 80155 |
| NARG1L | 79612 |
| NARG2 | 79664 |
| NASP | 4678 |
| NAT1 | 9 |
| NAT13 | 80218 |
| NAT2 | 10 |
| NBN | 4683 |
| NCK1 | 4690 |
| NCOA4 | 8031 |
| NDC80 | 10403 |
| NDST3 | 9348 |
| NDST4 | 64579 |
| NDUFB3 | 4709 |
| NDUFS1 | 4719 |
| NEDD1 | 121441 |
| NEUROD4 | 58158 |
| NFAT5 | 10725 |
| NHEDC1 | 150159 |
| NHEDC2 | 133308 |
| NLGN1 | 22871 |
| NLRP14 | 338323 |
| NME5 | 8382 |
| NOC3L | 64318 |
| NOSTRIN | 115677 |
| NPAL1 | 152519 |
| NPAT | 4863 |
| NPFFR2 | 10886 |
| NPVF | 64111 |
| NPY1R | 4886 |
| NPY5R | 4889 |
| NRBF2 | 29982 |
| NRIP1 | 8204 |
| NSBP1 | 79366 |
| NSUN3 | 63899 |
| NSUN6 | 221078 |
| NT5C3 | 51251 |
| NTS | 4922 |
| NUCB2 | 4925 |
| NUDT12 | 83594 |
| NUFIP2 | 57532 |
| NUP153 | 9972 |
| NUP155 | 9631 |
| NUP62CL | 54830 |
| NUP98 | 4928 |
| NUPL2 | 11097 |
| OCIAD1 | 54940 |
| OCIAD2 | 132299 |
| ODAM | 54959 |
| ODF2L | 57489 |
| OLA1 | 29789 |
| OLAH | 55301 |
| OMA1 | 115209 |
| OMD | 4958 |
| OMG | 4974 |
| OR10J5 | 127385 |
| OR14A16 | 284532 |
| OR2AG1 | 144125 |
| OR2AG2 | 338755 |
| OR4C3 | 256144 |
| OR51B2 | 79345 |
| OR51B6 | 390058 |
| OR52A1 | 23538 |
| OR52A5 | 390054 |
| OR5P3 | 120066 |
| OR6A2 | 8590 |
| ORC2L | 4999 |
| ORC4L | 5000 |
| OSBPL9 | 114883 |
| OSMR | 9180 |
| OSTBETA | 123264 |
| OXR1 | 55074 |
| OXSM | 54995 |
| P2RY13 | 53829 |
| P2RY5 | 10161 |
| PAIP1 | 10605 |
| PALB2 | 79728 |
| PALMD | 54873 |
| PARP9 | 83666 |
| PBK | 55872 |
| PCMTD1 | 115294 |
| PDC | 5132 |
| PDCD1LG2 | 80380 |
| PDE5A | 8654 |
| PDS5B | 23047 |
| PDZK1 | 5174 |
| PEX12 | 5193 |
| PEX3 | 8504 |
| PGDS | 27306 |
| PGK2 | 5232 |
| PGM3 | 5238 |
| PHF11 | 51131 |
| PHF3 | 23469 |
| PHF6 | 84295 |
| PHOSPHO2 | 493911 |
| PIGF | 5281 |
| PIH1D2 | 120379 |
| PJA2 | 9867 |
| PKD2L2 | 27039 |
| PKIA | 5569 |
| PLA2G4A | 5321 |
| PLA2G7 | 7941 |
| PLAA | 9373 |
| PLCZ1 | 89869 |
| PLEK | 5341 |
| PLEKHA8 | 84725 |
| PLGLB2 | 5342 |
| PLK4 | 10733 |
| PLS1 | 5357 |
| PLS3 | 5358 |
| PLSCR1 | 5359 |
| PLSCR2 | 57047 |
| PMCH | 5367 |
| PMS1 | 5378 |
| PNLIPRP3 | 119548 |
| PNPLA8 | 50640 |
| PNRC2 | 55629 |
| POF1B | 79983 |
| POLK | 51426 |
| POLR3G | 10622 |
| POMP | 51371 |
| POT1 | 25913 |
| PPIL3 | 53938 |
| PPP1R3A | 5506 |
| PPP1R9A | 55607 |
| PPP2R3C | 55012 |
| PRB1 | 5542 |
| PRB2 | 653247 |
| PRDM4 | 11108 |
| PRDX1 | 5052 |
| PRKACB | 5567 |
| PRLR | 5618 |
| PRR13 | 54458 |
| PSIP1 | 11168 |
| PSMA4 | 5685 |
| PSMA5 | 5686 |
| PSMAL | 219595 |
| PSME4 | 23198 |
| PTH | 5741 |
| PTPLAD2 | 401494 |
| PTPN12 | 5782 |
| PTPN22 | 26191 |
| PTPRC | 5788 |
| PTPRZ1 | 5803 |
| PTTG1 | 9232 |
| PUS10 | 150962 |
| PUS7L | 83448 |
| PXMP3 | 5828 |
| PXT1 | 222659 |
| R3HDM1 | 23518 |
| RAB23 | 51715 |
| RAD1 | 5810 |
| RAD17 | 5884 |
| RAD23B | 5887 |
| RAD51AP1 | 10635 |
| RAD54B | 25788 |
| RAD9B | 144715 |
| RAG2 | 5897 |
| RALGPS2 | 55103 |
| RANBP2 | 5903 |
| RANBP6 | 26953 |
| RASSF6 | 166824 |
| RB1CC1 | 9821 |
| RBAK | 57786 |
| RBM43 | 375287 |
| REG3G | 130120 |
| REP15 | 387849 |
| REST | 5978 |
| REV3L | 5980 |
| RFESD | 317671 |
| RG9MTD1 | 54931 |
| RGPD2 | 729857 |
| RGS1 | 5996 |
| RGS13 | 6003 |
| RGS17 | 26575 |
| RGS18 | 64407 |
| RGS4 | 5999 |
| RHBDL2 | 54933 |
| RMI1 | 80010 |
| RNASE10 | 338879 |
| RNASE6 | 6039 |
| RNASE8 | 122665 |
| RNASEH2B | 79621 |
| RNF133 | 168433 |
| RNF14 | 9604 |
| RNF17 | 56163 |
| RNF180 | 285671 |
| RNF219 | 79596 |
| RNF24 | 11237 |
| RNFT1 | 51136 |
| RNMT | 8731 |
| ROS1 | 6098 |
| RP1 | 6101 |
| RP4-692D3.1 | 728621 |
| RPAP3 | 79657 |
| RPE | 6120 |
| RPGR | 6103 |
| RPP14 | 11102 |
| RPS6KC1 | 26750 |
| RPTN | 126638 |
| RTKN2 | 219790 |
| RTN4 | 57142 |
| RWDD1 | 51389 |
| RWDD2A | 112611 |
| RXFP1 | 59350 |
| S100A10 | 6281 |
| S100G | 795 |
| S100PBP | 64766 |
| SACS | 26278 |
| SAMSN1 | 64092 |
| SASS6 | 163786 |
| SC4MOL | 6307 |
| SCEL | 8796 |
| SCFD1 | 23256 |
| SCG3 | 29106 |
| SCGB3A2 | 117156 |
| SCLT1 | 132320 |
| SCOC | 60592 |
| SCP2 | 6342 |
| SCRN3 | 79634 |
| SCYE1 | 9255 |
| SCYL2 | 55681 |
| SEC23A | 10484 |
| SEC62 | 7095 |
| SELE | 6401 |
| SENP8 | 123228 |
| SEPP1 | 6414 |
| SEPSECS | 51091 |
| SERINC4 | 619189 |
| SERPINB5 | 5268 |
| SERPINI1 | 5274 |
| SERPINI2 | 5276 |
| SETD2 | 29072 |
| SETDB2 | 83852 |
| SFRS12IP1 | 285672 |
| SFRS2IP | 9169 |
| SGK3 | 23678 |
| SGOL1 | 151648 |
| SGOL2 | 151246 |
| SGTB | 54557 |
| SH3BGR | 6450 |
| SHOC2 | 8036 |
| SKAP2 | 8935 |
| SKIL | 6498 |
| SKP1 | 6500 |
| SLAMF9 | 89886 |
| SLC15A2 | 6565 |
| SLC16A4 | 9122 |
| SLC16A9 | 220963 |
| SLC17A3 | 10786 |
| SLC23A3 | 151295 |
| SLC25A40 | 55972 |
| SLC26A2 | 1836 |
| SLC26A7 | 115111 |
| SLC28A2 | 9153 |
| SLC31A1 | 1317 |
| SLC35A1 | 10559 |
| SLC35A3 | 23443 |
| SLC35A5 | 55032 |
| SLC35B3 | 51000 |
| SLC38A9 | 153129 |
| SLC39A10 | 57181 |
| SLC39A12 | 221074 |
| SLC41A2 | 84102 |
| SLC6A14 | 11254 |
| SLC6A15 | 55117 |
| SLC7A13 | 157724 |
| SLCO1A2 | 6579 |
| SLCO1B1 | 10599 |
| SLCO1B3 | 28234 |
| SLCO1C1 | 53919 |
| SLFN12 | 55106 |
| SLITRK6 | 84189 |
| SLPI | 6590 |
| SLU7 | 10569 |
| SMARCAD1 | 56916 |
| SMC2 | 10592 |
| SMNDC1 | 10285 |
| SMR3A | 26952 |
| SNAP23 | 8773 |
| SNCA | 6622 |
| SNRPB2 | 6629 |
| SNX14 | 57231 |
| SNX16 | 64089 |
| SP1 | 6667 |
| SPAM1 | 6677 |
| SPARCL1 | 8404 |
| SPATA16 | 83893 |
| SPATA9 | 83890 |
| SPDYA | 245711 |
| SPPL2A | 84888 |
| SPRR1A | 6698 |
| SPRR1B | 6699 |
| SPRR2D | 6703 |
| SPRR2E | 6704 |
| SPRR2G | 6706 |
| SPRR3 | 6707 |
| SPRY1 | 10252 |
| SPRYD5 | 84767 |
| SPZ1 | 84654 |
| SRD5A2L2 | 253017 |
| SRP54 | 6729 |
| SRR | 63826 |
| SSB | 6741 |
| SSX2IP | 117178 |
| STAG2 | 10735 |
| STAM2 | 10254 |
| STEAP1 | 26872 |
| STEAP2 | 261729 |
| STK17B | 9262 |
| STK31 | 56164 |
| STK38L | 23012 |
| STX17 | 55014 |
| STX19 | 415117 |
| STX7 | 8417 |
| STXBP4 | 252983 |
| STYX | 6815 |
| SUB1 | 10923 |
| SULT1B1 | 27284 |
| SULT1E1 | 6783 |
| SULT2A1 | 6822 |
| SUMO1 | 7341 |
| SYCP1 | 6847 |
| SYCP2 | 10388 |
| SYCP3 | 50511 |
| SYT4 | 6860 |
| SYTL2 | 54843 |
| TAAR1 | 134864 |
| TAF2 | 6873 |
| TAF9 | 6880 |
| TAS2R10 | 50839 |
| TAS2R4 | 50832 |
| TATDN1 | 83940 |
| TAX1BP1 | 8887 |
| TBC1D15 | 64786 |
| TBC1D19 | 55296 |
| TBC1D8B | 54885 |
| TC2N | 123036 |
| TCEAL4 | 79921 |
| TCHHL1 | 126637 |
| TCP1 | 6950 |
| TCP11 | 6954 |
| TCTN3 | 26123 |
| TDO2 | 6999 |
| TDRD1 | 56165 |
| TEX11 | 56159 |
| TEX12 | 56158 |
| TEX15 | 56154 |
| TFEC | 22797 |
| TGS1 | 96764 |
| THAP5 | 168451 |
| THNSL1 | 79896 |
| THOC2 | 57187 |
| TIMM9 | 26520 |
| TIPARP | 25976 |
| TIPIN | 54962 |
| TLR1 | 7096 |
| TLR10 | 81793 |
| TLR2 | 7097 |
| TLR3 | 7098 |
| TLR4 | 7099 |
| TLR5 | 7100 |
| TLR6 | 10333 |
| TLR7 | 51284 |
| TM4SF20 | 79853 |
| TMCO2 | 127391 |
| TMCO5 | 145942 |
| TMEM106B | 54664 |
| TMEM126A | 84233 |
| TMEM126B | 55863 |
| TMEM128 | 85013 |
| TMEM135 | 65084 |
| TMEM144 | 55314 |
| TMEM161B | 153396 |
| TMEM167 | 153339 |
| TMEM27 | 57393 |
| TMEM34 | 55751 |
| TMEM45A | 55076 |
| TMEM60 | 85025 |
| TMEM67 | 91147 |
| TMEM68 | 137695 |
| TMEM77 | 128338 |
| TMPRSS11F | 389208 |
| TMSL8 | 11013 |
| TMTC3 | 160418 |
| TNFSF4 | 7292 |
| TOR1AIP2 | 163590 |
| TOX4 | 9878 |
| TP53INP1 | 94241 |
| TPRKB | 51002 |
| TREML4 | 285852 |
| TRHR | 7201 |
| TRIM13 | 10206 |
| TRIM36 | 55521 |
| TRIM59 | 286827 |
| TRIM60 | 166655 |
| TRIM69 | 140691 |
| TRMT5 | 57570 |
| TRNT1 | 51095 |
| TROVE2 | 6738 |
| TRPM7 | 54822 |
| TSHB | 7252 |
| TSNAX | 7257 |
| TSPAN12 | 23554 |
| TTC33 | 23548 |
| TUBD1 | 51174 |
| TXNDC10 | 54495 |
| TXNDC16 | 57544 |
| TXNDC9 | 10190 |
| TXNL4B | 54957 |
| UACA | 55075 |
| UBA5 | 79876 |
| UBA6 | 55236 |
| UBE2T | 29089 |
| UBE2U | 148581 |
| UBLCP1 | 134510 |
| UBQLNL | 143630 |
| UBR1 | 197131 |
| UEVLD | 55293 |
| UGT2A1 | 10941 |
| UGT2A3 | 79799 |
| UGT2B11 | 10720 |
| UGT2B15 | 7366 |
| UGT2B17 | 7367 |
| UGT2B28 | 54490 |
| UGT2B4 | 7363 |
| UGT2B7 | 7364 |
| UGT3A1 | 133688 |
| UGT8 | 7368 |
| UHRF1BP1L | 23074 |
| USH2A | 7399 |
| USMG5 | 84833 |
| USP1 | 7398 |
| USP16 | 10600 |
| USP26 | 83844 |
| USP29 | 57663 |
| USP33 | 23032 |
| USP37 | 57695 |
| USP45 | 85015 |
| USP53 | 54532 |
| USP9Y | 8287 |
| USPL1 | 10208 |
| UTP14C | 9724 |
| VAMP4 | 8674 |
| VCAN | 1462 |
| VDAC3 | 7419 |
| VEPH1 | 79674 |
| VIP | 7432 |
| VNN2 | 8875 |
| VNN3 | 55350 |
| VPS13A | 23230 |
| VPS13C | 54832 |
| VPS26A | 9559 |
| VPS29 | 51699 |
| VPS37A | 137492 |
| VPS54 | 51542 |
| VRK2 | 7444 |
| WBP4 | 11193 |
| WBP5 | 51186 |
| WDR12 | 55759 |
| WDR17 | 116966 |
| WDR78 | 79819 |
| XIAP | 331 |
| XPO1 | 7514 |
| XRCC4 | 7518 |
| XRN1 | 54464 |
| YIPF4 | 84272 |
| YIPF5 | 81555 |
| ZADH1 | 145482 |
| ZBBX | 79740 |
| ZBTB1 | 22890 |
| ZBTB25 | 7597 |
| ZBTB26 | 57684 |
| ZBTB6 | 10773 |
| ZC3H15 | 55854 |
| ZCCHC10 | 54819 |
| ZCCHC17 | 51538 |
| ZCCHC5 | 203430 |
| ZCCHC6 | 79670 |
| ZCRB1 | 85437 |
| ZFAND5 | 7763 |
| ZFAND6 | 54469 |
| ZFP14 | 57677 |
| ZFP2 | 80108 |
| ZFP3 | 124961 |
| ZFP30 | 22835 |
| ZFP90 | 146198 |
| ZFY | 7544 |
| ZFYVE16 | 9765 |
| ZFYVE9 | 9372 |
| ZHX1 | 11244 |
| ZIK1 | 284307 |
| ZMAT1 | 84460 |
| ZMYM1 | 79830 |
| ZMYM6 | 9204 |
| ZNF107 | 51427 |
| ZNF121 | 7675 |
| ZNF140 | 7699 |
| ZNF148 | 7707 |
| ZNF155 | 7711 |
| ZNF17 | 7565 |
| ZNF175 | 7728 |
| ZNF18 | 7566 |
| ZNF181 | 339318 |
| ZNF182 | 7569 |
| ZNF184 | 7738 |
| ZNF189 | 7743 |
| ZNF223 | 7766 |
| ZNF228 | 7771 |
| ZNF23 | 7571 |
| ZNF232 | 7775 |
| ZNF233 | 353355 |
| ZNF235 | 9310 |
| ZNF256 | 10172 |
| ZNF260 | 339324 |
| ZNF266 | 10781 |
| ZNF271 | 10778 |
| ZNF28 | 7576 |
| ZNF280C | 55609 |
| ZNF280D | 54816 |
| ZNF285A | 26974 |
| ZNF286A | 57335 |
| ZNF287 | 57336 |
| ZNF294 | 26046 |
| ZNF320 | 162967 |
| ZNF334 | 55713 |
| ZNF347 | 84671 |
| ZNF35 | 7584 |
| ZNF354B | 117608 |
| ZNF354C | 30832 |
| ZNF383 | 163087 |
| ZNF415 | 55786 |
| ZNF426 | 79088 |
| ZNF43 | 7594 |
| ZNF438 | 220929 |
| ZNF449 | 203523 |
| ZNF45 | 7596 |
| ZNF451 | 26036 |
| ZNF452 | 114821 |
| ZNF454 | 285676 |
| ZNF470 | 388566 |
| ZNF471 | 57573 |
| ZNF480 | 147657 |
| ZNF484 | 83744 |
| ZNF493 | 284443 |
| ZNF528 | 84436 |
| ZNF545 | 284406 |
| ZNF559 | 84527 |
| ZNF560 | 147741 |
| ZNF561 | 93134 |
| ZNF562 | 54811 |
| ZNF566 | 84924 |
| ZNF568 | 374900 |
| ZNF569 | 148266 |
| ZNF570 | 148268 |
| ZNF571 | 51276 |
| ZNF577 | 84765 |
| ZNF582 | 147948 |
| ZNF583 | 147949 |
| ZNF585A | 199704 |
| ZNF585B | 92285 |
| ZNF606 | 80095 |
| ZNF611 | 81856 |
| ZNF638 | 27332 |
| ZNF639 | 51193 |
| ZNF642 | 339559 |
| ZNF643 | 65243 |
| ZNF644 | 84146 |
| ZNF655 | 79027 |
| ZNF658 | 26149 |
| ZNF664 | 144348 |
| ZNF670 | 93474 |
| ZNF677 | 342926 |
| ZNF678 | 339500 |
| ZNF699 | 374879 |
| ZNF700 | 90592 |
| ZNF706 | 51123 |
| ZNF708 | 7562 |
| ZNF709 | 163051 |
| ZNF711 | 7552 |
| ZNF770 | 54989 |
| ZNF776 | 284309 |
| ZNF781 | 163115 |
| ZNF782 | 158431 |
| ZNF790 | 388536 |
| ZNF804A | 91752 |
| ZNF816A | 125893 |
| ZNF828 | 283489 |
| ZNF829 | 374899 |
| ZNF85 | 7639 |
| ZNF91 | 7644 |
| ZSWIM2 | 151112 |
| ZUFSP | 221302 |
| ZWILCH | 55055 |
| ZZZ3 | 26009 |
